# Supplementary material for: Dimerization/Elimination of β-Styrylmalonates under Action of TiCl4
Source: Molecules. 2022 Dec 29;28(1):270. doi: 10.3390/molecules28010270 (PMC9822291; doi:10.3390/molecules28010270)
Supplement: Supplementary file 1 [file molecules-28-00270-s001.zip › molecules-2118593-supplementary.pdf]

Dimerization/elimination of β-styrylmalonates under action of $TiCl_4$

D. D. Borisov, G. R. Chermashentsev, K. V. Potapov, R. A. Novikov, and Yu.V. Tomilov*

N. D. Zelinsky Institute of Organic Chemistry, Russian Academy of Sciences, 47 Leninsky prosp., 119991 Moscow, Russian Federation

* *E-mail*: *novikovffff@bk.ru, tom@ioc.ac.ru*

SUPPORTING INFORMATION

Table of contents:

Entry	Description	Page
**1.**	Experimental section	**S2**
**1.1.**	General experimental details	**S2**
**1.2.**	General synthetic procedure and spectroscopic data for trimethyl 2-(arylmethyl)-4-hydroxy-[1,1'-biaryl]-3,3,5(2*H*)-tricarboxylates (**2**)	**S3**
**1.3.**	Cross-reaction of β-styrylmalonates **1a,f** with benzylidenemalonate	**S7**
**1.4.**	General synthetic procedure and spectroscopic data for trimethyl 7-hydroxy-11-aryl-9,10-dihydro-5,9-methanobenzo[8]annulene-6,8,8(5*H*)-tricarboxylates (**3**)	**S8**
**2.**	References	**S10**
**3.**	NMR spectra for the new compounds	**S11**

1. Experimental section

1.1. General experimental details

All reagents and solvents were used commercial grade chemicals without additional purification. All operations with $TiCl_4$ were carried out under dry argon atmosphere. TLC analysis was performed on Silufol chromatographic plates. For preparative chromatography, silica gel 60 (0.040–0.063 mm) was used. ^{1}H, ^{13}C, ^{19}F NMR spectra were recorded on a Bruker AVANCE II 300 MHz (300.1, 75.5 MHz and 282.4 MHz respectively) and a Bruker AMX III 400 MHz (400.1, 100.6 MHz and 376.5 MHz respectively) spectrometers in $CDCl_3$, containing 0.05% Me_4Si as the internal standard. Determinations of structures and stereochemistry of obtained compounds and assignments of ^{1}H and ^{13}C signals were made with the aid of 1D and 2D DEPT, COSY, TOCSY, NOESY, HSQC, HMBC spectra. IR spectra were obtained on a FT-IR spectrometer in $CHCl_3$ solution (1%). Mass spectra were recorded using electron impact ionization (EI, 70 eV, direct inlet probe). High resolution mass spectra were obtained using simultaneous electospray (ESI TOF). The melting points were determined on Kofler hot-stage microscope. Starting β–styrylmalonates **1a–g** were synthesized from the corresponding donor–acceptor cyclopropanes through isomerisation reactions.[S1-2]

1.2. General synthetic procedure and spectroscopic data for trimethyl 2-(arylmethyl)-4-hydroxy-[1,1´-biaryl]-3,3,5(2*H*)-tricarboxylates (2)

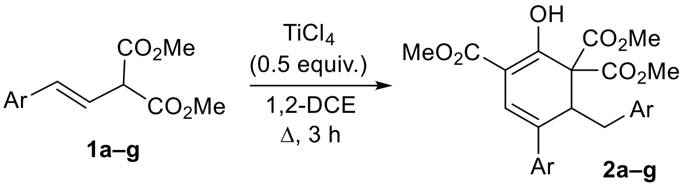

All operations were performed under dry argon atmosphere. A solution of styrylmalonate **1a–g** (0.34 mmol, 1 equiv.) and $TiCl_4$ (0.17 mmol, 0.5 equiv.) in dry 1,2-DCE (3 mL) was stirred at reflux for 3 h. Then an aqueous solution of HCl (10%) was added at room temperature and the reaction mixture was extracted with CH_2Cl_2 (3×10 mL). The organic layer washed the brine and dried over $MgSO_4$. The solvent was removed *in vacuo*. The products **2a–e** were obtained without any purification. The compounds **2f,g** were purified by column chromatography on silica gel (eluenf: benzene to benzene–EtOAc, 20:1).

The resulting compounds can be additionally purified on a Silufol chromatographic plate (20×20 cm) using hexane–acetone (4:1) as eluent to afford the pure products.

Trimethyl 2-benzyl-4-hydroxy-[1,1´-biphenyl]-3,3,5(2H)-tricarboxylate (2a)

The compound **2a** was prepared in yield 69 mg (93%). Colorless thick oil. IR ($CHCl_3$): $\tilde{\nu}$ 3174 (OH), 3067, 3055, 3039, 3032, 3016, 1740 br (C=O), 1656, 1590, 1497, 1437, 1269, 12382 cm^{-1}.

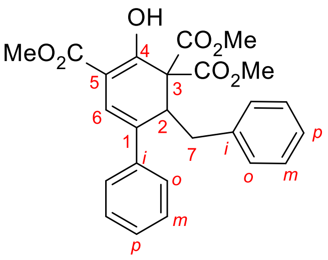

^{1}H NMR (400.1 MHz, $CDCl_3$): δ 2.88 (dd, 1H, H_a(7), ^2J = 14.2 Hz, ^3J = 6.6 Hz), 3.08 (dd, 1H, H_b(7), ^2J = 14.2 Hz, ^3J = 6.6 Hz), 3.62, 3.75 and 3.88 (all s, 3 × 3H, 3 CO_2Me), 4.17 (t, 1H, H(2), ^3J = 6.6 Hz), 6.45 (s, 1H, H(6)), 6.90–7.54 (m, 10H, 4 H(*o*), 4 H(*m*) and 2 H(*p*)), 12.86 (s, 1H, OH) ppm. ^{13}C NMR (100.6 MHz, $CDCl_3$): δ 34.1 (C(7)), 43.4 (C(2)), 52.1, 52.8 and 53.5 (3 OMe), 63.6 (C(3)), 100.9 (C(5)), 116.0 (C(6)), 125.8 (C(*p*)), 126.3 (2 C(*o*)), 126.9 (C(*p*)), 127.5 (2 C(*m*)), 128.1 (2 C(*m*)), 129.4 (2 C(*o*)), 134.1 (C(1)), 138.1 (C(*i*)), 139.2 (C(*i*)), 165.8 (C(4)), 166.5, 167.8 and 170.7 (3 COO). MS (*m/z*, %): 436 (1, M$^+$), 384 (1), 345 (2), 328 (2), 313 (5), 286 (5), 269 (7), 254 (7), 226 (7), 215 (15), 196 (16), 178 (7), 165 (10), 152 (9), 139 (27), 127 (9), 115 (28), 105 (75), 91 (100), 77 (16), 59 (10), 44 (8). HRMS calcd for $C_{25}H_{24}O_7$ (*M*): *M+Na*, 459.1414. Found: *m/z* 459.1336.

Trimethyl 3´-bromo-2-(3-bromobenzyl)-4-hydroxy-[1,1´-biphenyl]-3,3,5(2H)-tricarboxylate (2b)

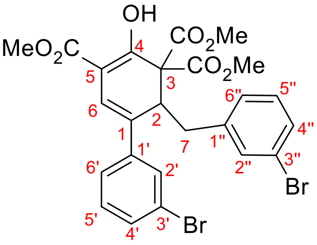

The compound **2b** was prepared in yield 84 mg (83%). Colorless thick oil. IR ($CHCl_3$): $\tilde{\nu}$ 3169 (OH), 3088, 3066, 3050, 3026, 1739 br (C=O), 1655, 1587, 1474, 1435, 1266, 1246, 1182 cm^{-1}.

^{1}H NMR (300.1 MHz, $CDCl_3$): δ 2.82 (dd, 1H, H_a(7), ^2J = 13.6 Hz, ^3J

= 9.1 Hz), 3.05 (dd, 1H, H$_b$(7), ^2J = 13.6 Hz, ^3J = 5.4 Hz), 3.76, 3.82 and 3.91 (all s, 3 × 3H, 3 CO_2Me), 4.00 (dd, 1H, H(2), ^3J = 9.1 Hz, ^3J = 5.4 Hz), 6.46 (s, 1H, H(6)), 6.78–7.53 (m, 8H, 8 H$_{Ar}$), 12.90 (s, 1H, OH) ppm. ^{13}C NMR (75.5 MHz, $CDCl_3$): δ 34.0 (C(7)), 43.5 (C(2)), 52.2, 53.1 and 53.6 (3 OMe), 63.8 (C(3)), 100.5 (C(5)), 117.5 (C(6)), 121.7 and 122.3 (C(3´) and C(3´´)), 124.8 and 129.8 (C(6´) and C(6´´)), 128.8 and 129.0 (C(5´) and C(5´´)), 129.1 and 132.7 (C(2´) and C(2´´)), 129.1 and 132.7 (C(4´) and C(4´´)), 132.3 (C(1)), 140.1 and 141.5 (C(1´) and C(1´´)), 166.3 (C(4)), 166.5, 167.6 and 170.5 (3 COO). MS (*m/z*, for ^{79}Br, %): 594 (1, M^+), 503 (1), 391 (11), 379 (3), 347 (25), 301 (16), 274 (21), 246 (3), 234 (3), 219 (20), 202 (10), 171 (61), 159 (14), 139 (15), 115 (42), 85 (100), 71 (50), 59 (21). HRMS calcd for $C_{25}H_{22}Br_2O_7$ (*M*, for ^{79}Br): *M+Na*, 614.9624. Found: *m/z* 614.9513.

Trimethyl 4-hydroxy-4´-nitro-2-(4-nitrobenzyl)-[1,1´-biphenyl]-3,3,5(2H)-tricarboxylate (2c)

The compound was prepared in yield 79 mg (88%). Red thick oil. IR ($CHCl_3$): $\tilde{\nu}$ 3170 (OH), 3053, 3028, 3023, 2957, 2928, 2855, 1738 br (C=O), 1658, 1581, 1523 br (N=O), 1447, 1347 br (N=O), 1258, 1182 cm^{-1}.

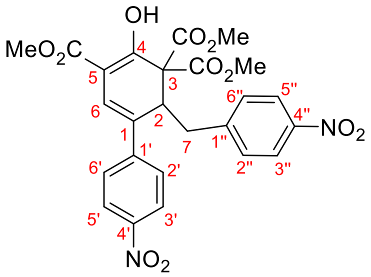

^{1}H NMR (300.1 MHz, $CDCl_3$): δ 3.04 (dd, 1H, H(7), ^2J = 13.6 Hz, ^3J = 8.6 Hz), 3.19 (dd, 1H, H(7), ^2J = 13.6 Hz, ^3J = 5.3 Hz), 3.74, 3.84 and 3.87 (all s, 3 × 3H, 3 CO_2Me), 4.13 (dd, 1H, H(2), ^3J = 8.6 Hz, ^3J = 5.3 Hz), 6.62 (s, 1H, H(6)), 7.18 (d, 2H, H(2´´) and H(6´´), ^3J = 8.6 Hz), 7.43 (d, 2H, H(2´) and H(6´), ^3J = 8.9 Hz), 7.87 (d, 2H, H(3´´) and H(5´´), ^3J = 8.6 Hz), 8.02 (d, 2H, H(3´) and H(5´), ^3J = 8.9 Hz), 13.02 (s, 1H, OH) ppm. ^{13}C NMR (75.5 MHz, $CDCl_3$): δ 34.2 (C(7)), 42.7 (C(2)), 52.4, 53.4 and 53.9 (3 OMe), 63.6 (C(3)), 100.5 (C(5)), 120.5 (C(6)), 122.7 (C(3´´) and C(5´´)), 123.6 (C(3´) and C(5´)), 126.4 (C(2´) and C(6´)), 130.4 (C(2´´) and C(6´´)), 130.8 (C(1)), 145.2 and 145.6 (C(1´) and C(1´´)), 146.4 and 146.5 (C(4´) and C(4´´)), 166.3, 167.3 and 170.2 (C(4) and 3 COO). MS (*m/z*, %): 526 (14, M^+), 468 (3), 435 (25), 403 (3), 358 (18), 346 (20), 326 (21), 314 (66), 300 (11), 268 (21), 256 (3), 241 (16), 222 (9), 203 (3), 191 (30), 152 (11), 138 (14), 115 (22), 91 (45), 78 (22), 59 (100). HRMS calcd for $C_{25}H_{22}N_2O_{11}$ (*M*): *M+H*, 527.1296. Found: *m/z* 527.1289.

Trimethyl 2´-chloro-2-(2-chlorobenzyl)-4-hydroxy-[1,1´-biphenyl]-3,3,5(2H)-tricarboxylate (2d)

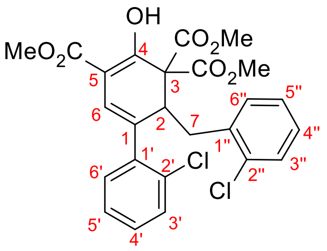

The compound **2d** was prepared in yield 72 mg (84%). Colorless thick oil. IR ($CHCl_3$): $\tilde{\nu}$ 3168 (OH), 3046, 3003, 2955, 1740 br (C=O), 1594, 1571, 1474, 1437, 1373, 1260, 1221, 1181 cm^{-1}.

^{1}H NMR (300.1 MHz, $CDCl_3$): δ 2.84 (dd, 1H, H$_a$(7), ^2J = 14.0 Hz, ^3J = 9.3 Hz), 3.23 (dd, 1H, H$_b$(7), ^2J = 14.0 Hz, ^3J = 6.3 Hz), 3.66, 3.76

and 3.88 (all s, 3 × 3H, 3 CO_2Me), 4.35 (dd, 1H, H(2), ^3J = 9.3 Hz, ^3J = 6.3 Hz), 6.61 (s, 1H, H(6)), 6.82–7.22 (m, 7H, 7 H_{Ar}),7.51 (dd, 1H, H_{Ar}, ^3J = 7.8 Hz, ^4J = 1.6 Hz), 12.95 (s, 1H, OH) ppm. ^{13}C NMR (75.5 MHz, $CDCl_3$): δ 32.8 (C(7)), 42.3 (C(2)), 52.3, 53.0 and 53.4 (3 OMe), 63.9 (C(3)), 100.3 (C(5)), 121.1 (C(6)), 126.2, 126.3, 127.5, 127.6, 128.7, 129.8, 130.4 and 131.7 (8 CH_{Ar}), 128.7 and 135.6 (C(1´) and C(1´´)), 132.2 and 134.1 (C(2´) and C(2´´)), 137.7 (C(1)), 166.2 (C(4)), 166.6, 167.5 and 170.9 (3 COO). MS (*m/z*, for ^{35}Cl, %): 504 (6, M^+), 413 (28), 381 (2), 347 (10), 335 (3), 303 (22), 289 (4), 275 (9), 257 (4), 230 (4), 215 (2), 202 (3), 173 (7), 159 (4), 149 (14), 139 (10), 125 (54), 115 (13), 89 (23), 59 (82), 15 (100). HRMS calcd for $C_{25}H_{22}Cl_2O_7$ (*M*, for ^{35}Cl): *M*+*Na*, 527.0635. Found: *m/z* 527.0623.

Trimethyl 4´-fluoro-2-(4-fluorobenzyl)-4-hydroxy-[1,1´-biphenyl]-3,3,5(2H)-tricarboxylate (2e)

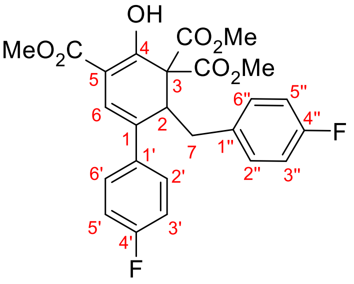

The compound **2e** was prepared in yield 59 mg (74%). Colorless thick oil. IR ($CHCl_3$): $\tilde{\nu}$ 3170 (O-H), 3048, 3027, 2956, 1737 br (C=O), 1659, 1603, 1511, 1438, 1376, 1250, 1228, 1182, 1159 cm^{-1}. ^{1}H NMR (300.1 MHz, $CDCl_3$): δ 2.83 (dd, 1H, H_a(7), ^2J = 14.0 Hz, ^3J = 7.9 Hz), 3.00 (dd, 1H, H_b(7), ^2J = 14.0 Hz, ^3J = 5.7 Hz), 3.72, 3.74 and 3.85 (all s, 3 × 3H, 3 CO_2Me), 3.99 (dd, 1H, H(2), ^3J = 7.9 Hz, ^3J = 5.7 Hz), 6.34 (s, 1H, H(6)), 6.70 (t, 2H, H(3´´) and H(5´´), $^3J_{HH}$ = $^3J_{HF}$ = 8.7 Hz), 6.79–7.74 (m, 6H, 6 H_{Ar}), 12.82 (s, 1H, OH) ppm. ^{13}C NMR (75.5 MHz, $CDCl_3$): δ 33.3 (C(7)), 43.9 (C(2)), 52.1, 53.0 and 53.6 (3 OMe), 63.7 (C(3)), 100.7 (C(5)), 114.1 (d, C(3´´) and C(5´´), $^2J_{CF}$ = 21.2 Hz), 114.9 (d, C(3´) and C(5´), $^2J_{CF}$ = 21.5 Hz), 116.2 (C(6)), 127.8 (d, C(2´) and C(6´), $^3J_{CF}$ = 8.0 Hz), 130.9 (d, C(2´´) and C(6´´), $^3J_{CF}$ = 7.8 Hz), 132.8 (C(1)), 133.7 (d, C(1´´), $^4J_{CF}$ = 3.1 Hz), 135.5 (d, C(1´), $^4J_{CF}$ = 3.2 Hz), 161.3 (d, C(4´´), $^1J_{CF}$ = 244.1 Hz), 161.9 (d, C(4´), $^1J_{CF}$ = 246.7 Hz), 165.8 (C(4)), 166.6, 167.8 and 170.6 (3 COO). ^{19}F NMR (282.4 MHz, $CDCl_3$): δ –115.3 (tt, 1F, F(4´), $^3J_{HF}$ = 8.4 Hz, $^4J_{HF}$ = 5.3 Hz), –117.2 (tt, 1F, F(4´´), $^3J_{HF}$ = 8.7 Hz, $^4J_{HF}$ = 5.4 Hz). MS (*m/z*, %): 472 (23, M^+), 414 (14), 382 (21), 355 (41), 331 (40), 319 (15), 287 (80), 267 (35), 253 (15), 241 (45), 213 (40), 201 (11), 170 (12), 157 (51), 146 (20), 133 (71), 123 (54), 109 (100), 95 (26), 83 (45), 59 (100), 45 (56), 15 (100). HRMS calcd for $C_{25}H_{22}F_2O_7$ (*M*): *M*+*Na*, 495.1226. Found: *m/z* 495.1209.

Trimethyl 4-hydroxy-4´-methyl-2-(4-methylbenzyl)-[1,1´-biphenyl]-3,3,5(2H)-tricarboxylate (2f)

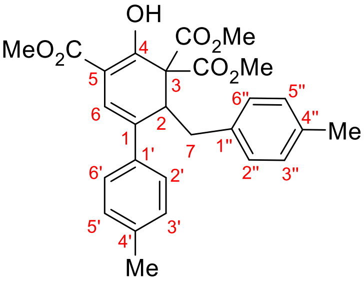

The compound **2f** was prepared in yield 37 mg (47%). Colorless thick oil. IR ($CHCl_3$): $\tilde{\nu}$ 3165 (OH), 3050, 3003, 2956, 2926, 1736 br (C=O), 1671, 1611, 1514, 1437, 1346, 1251, 1182 cm^{-1}. ^{1}H NMR (400.1 MHz, $CDCl_3$): δ 2.18 and 2.30 (both s, 2 × 3H, 2 Me), 2.83 (dd, 1H, H_a(7), ^2J = 14.4 Hz, ^3J = 6.3 Hz), 2.98 (dd, 1H,

H$_b$(7), ^2J = 14.4 Hz, ^3J = 6.3 Hz), 3.56, 3.70 and 3.83 (all s, 3 × 3H, 3 CO_2Me), 4.09 (t, 1H, H(2), ^3J = 6.3 Hz), 6.37 (s, 1H, H(6)), 6.86 (d, 2H, H(3´´) and H(5´´), ^3J = 7.9 Hz), 6.93 (d, 2H, H(2´´) and H(6´´), ^3J = 7.9 Hz), 7.04 (d, 2H, H(3´) and H(5´), ^3J = 8.1 Hz), 7.29 (d, 2H, H(2´) and H(6´), ^3J = 8.1 Hz), 12.76 (s, 1H, OH) ppm. ^{13}C NMR (100.6 MHz, $CDCl_3$): δ 20.8 and 21.0 (2 Me), 33.5 (C(7)), 43.4 (C(2)), 52.0, 52.7 and 53.5 (3 OMe), 63.5 (C(3)), 100.9 (C(5)), 115.2 (C(6)), 126.2 (C(2´) and C(6´)), 128.1 (C(3´´) and C(5´´)), 128.9 (C(3´) and C(5´)), 129.2 (C(2´´) and C(6´´)), 134.0 (C(1)), 135.1 and 135.2 (C(4´) and C(4´´)), 136.2 and 136.8 (C(1´) and C(1´´)), 165.5, 166.6, 167.9 and 170.7 (C(4) and 3 COO). MS (*m/z*, %): 464 (25, M$^+$), 406 (19), 374 (14), 347 (100), 327 (55), 315 (36), 304 (19), 283 (72), 268 (19), 259 (44), 237 (39), 215 (30), 202 (25), 184 (14), 165 (8), 153 (25), 141 (14), 128 (50), 115 (28), 105 (80), 91 (25), 77 (19), 59 (50), 15 (42). HRMS calcd for $C_{27}H_{28}O_7$ (*M*): *M*+*Na*, 487.1727. Found: *m/z* 487.1711.

Trimethyl 2-hydroxy-5-(naphthalen-1-yl)-6-(naphthalen-1-ylmethyl)cyclohexa-2,4-diene-1,1,3-tricarboxylate (2g)

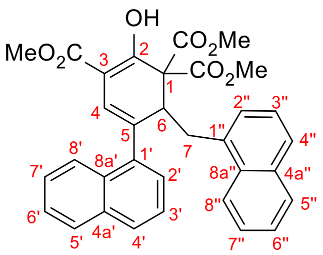

The compound **2g** was prepared in yield 19 mg (21%). Colorless thick oil. IR ($CHCl_3$): $\tilde{\nu}$ 3175 (OH), 3044, 3020, 2955, 2928, 2854, 1735 br (C=O), 1659, 1597, 1510, 1438, 1369, 1370, 1258, 1183 cm^{-1}.

^{1}H NMR (300.1 MHz, $CDCl_3$): δ 3.23 (dd, 1H, H$_a$(7), ^2J = 16.4 Hz, ^3J = 7.3 Hz), 3.51 (s, 3H, CO_2Me), 3.67 (dd, 1H, H$_b$(7), ^2J = 16.4 Hz, ^3J = 7.3 Hz), 3.88 and 3.91 (both s, 2 × 3H, 2 CO_2Me), 4.59 (t, 1H, H(6), ^3J = 7.3 Hz), 6.43 (s, 1H, H(4)), 6.89 (t, 1H, H$_{Ar}$, ^3J = 6.8 Hz), 7.14 (d, 1H, H$_{Ar}$, ^3J = 6.8 Hz), 7.19–8.13 (m, 12H, 12 H$_{Ar}$), 12.90 (s, 1H, OH) ppm. ^{13}C NMR (75.5 MHz, $CDCl_3$): δ 30.9 (C(7)), 45.0 (C(6)), 52.2, 52.7 and 53.3 (3 OMe), 64.0 (C(1)), 100.6 (C(3)), 120.2 (C(4)), 123.5–128.4 (14 CH$_{Ar}$), 131.3, 131.7, 131.8, 133.3, 133.7 and 133.9 (6 C$_{Ar}$), 137.5 (C(5)), 166.4 (C(2)), 166.8, 167.8 and 170.9 (3 COO). MS (*m/z*, %): 536 (4, M$^+$), 363 (14), 351 (4), 319 (22), 304 (60), 289 (7), 273 (10), 246 (5), 210 (9), 189 (27), 165 (50), 152 (33), 141 (100), 127 (11), 115 (36), 100 (9), 59 (34), 44 (21). HRMS calcd for $C_{33}H_{28}O_7$ (*M*): *M*+*Na*, 559.1727. Found: *m/z* 559.1708.

1.3. Cross-reaction of β-styrylmalonates 1a,f with benzylidenemalonate

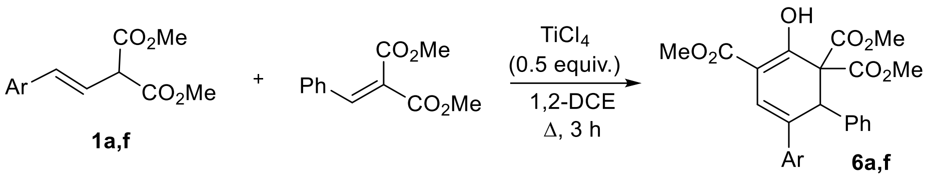

All operations were performed under dry argon atmosphere. A solution of styrylmalonate **1a,f** (0.34 mmol, 1 equiv.), dimethyl 2-benzylidenemalonate **5** (0.68 mmol, 2 equiv.) and $TiCl_4$ (0.17 mmol, 0.5 equiv.) in dry 1,2-DCE (3 mL) was stirred at reflux for 3 h. Then an aqueous solution of HCl (10%) was added at room temperature and the reaction mixture was extracted with CH_2Cl_2 (3×10 mL). The organic layer washed the brine and dried over $MgSO_4$. The solvent was removed *in vacuo*. The products **6a,f** were purified by column chromatography on silica gel (benzene to benzene–EtOAc, 20:1). The resulting compounds can be additionally purified on a Silufol chromatographic plate (20×20 cm) using hexane–acetone (5:1) eluent to afford the pure products.

Trimethyl 4´-hydroxy-[1,1´:2´,1´´-terphenyl]-3´,3´,5´(2´H)-tricarboxylate (6a)

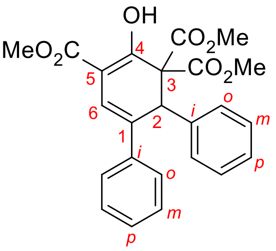

The compound **6a** was prepared from **1a** and **5** in yield 65 mg (45%) after several purifications on a Silufol chromatographic plate (20×20 cm) using hexane–acetone (5:1) as eluent. Colorless thick oil. IR ($CHCl_3$): $\tilde{\nu}$ 3169 (O-H), 3050, 3003, 2956, 1737 br (C=O), 1521, 1495, 1437, 1249, 1181 cm^{-1}.

^{1}H NMR (400.1 MHz, $CDCl_3$): δ 3.42, 3.84 and 3.96 (all s, 3 × 3H, 3 CO_2Me), 4.99 (s, 1H, H(2)), 6.78 (s, 1H, H(6)), 6.94–7.57 (m, 10H, 4 H(*o*), 4 H(*m*) and 2 H(*p*)), 12.88 (s, 1H, OH) ppm. ^{13}C NMR (75.5 MHz, $CDCl_3$): δ 49.6 (C(2)), 52.2, 52.5 and 53.8 (3 OMe), 65.7 (C(3)), 101.5 (C(5)), 116.2 (C(6)), 125.9 (2 C(*o*)), 127.3 and 127.9 (2 C(*p*)), 128.4, 128.5 and 128.8 (2 C(*o*) and 4 C(*m*)), 132.2 (C(1)), 135.8 (C(*i*)), 138.9 (C(*i*)), 165.1 (C(4)), 166.4, 167.1 and 170.9 (3 COO). MS (*m/z*, %): 422 (4, M^+), 331 (54), 322 (20), 313 (7), 299 (73), 287 (4), 273 (5), 262 (18), 231 (11), 215 (32), 204 (100), 191 (16), 165 (15), 152 (11), 139 (36), 129 (5), 115 (18), 105 (11), 91 (11), 77 (6), 59 (23). HRMS calcd for $C_{24}H_{22}O_7$ (*M*): *M+Na*, 445.1258. Found: *m/z* 445.1155.

Trimethyl 4´-hydroxy-4-methyl-[1,1´:2´,1´´-terphenyl]-3´,3´,5´(2´H)-tricarboxylate (6f)

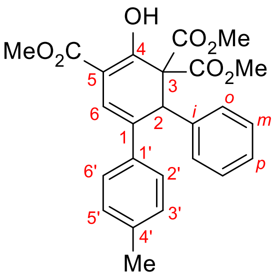

The compound **6f** was prepared from **1f** and **5** in yield 73 mg (49%). Light green thick oil. IR ($CHCl_3$): $\tilde{\nu}$ 3034, 3012, 2955, 2927, 1737 br (C=O), 1659, 1594, 1514, 1494, 1441, 1376, 1350, 1253, 1181, 1165 cm^{-1}.

^{1}H NMR (300.1 MHz, $CDCl_3$): δ 2.26 (s, 3H, Me), 3.39, 3.81 and 3.93 (all s, 3 × 3H, 3 CO_2Me), 4.95 (s, 1H, H(2)), 6.72 (s, 1H, H(6)), 6.94–7.36 (m, 9H, 9 H_{Ar}), 12.82 (s, 1H, OH) ppm. ^{13}C NMR (75.5 MHz, $CDCl_3$): δ 21.0 (Me), 49.4 (C(2)), 52.2, 52.5 and 53.8 (3 OMe), 65.7 (C(3)), 101.5 (C(5)), 115.3 (C(6)), 125.8 (C(2´) and C(6´)), 127.9

(C(*p*)), 128.5 and 128.8 (2 C(*o*) and 2 C(*m*)), 129.1 (C(3´) and C(5´)), 132.1 (C(1)), 135.8 and 135.9 (C(*i*) and C(1´)), 137.1 (C(4´)), 164.8 (C(4)), 166.4, 167.1 and 170.9 (3 COO). MS (*m/z*, %): 436 (12, M^+), 364 (29), 345 (40), 313 (58), 286 (8), 273 (9), 258 (8), 245 (17), 232 (54), 215 (31), 202 (21), 189 (17), 165 (10), 153 (17), 129 (33), 115 (21), 105 (22), 91 (19), 77 (12), 59 (100), 15 (29). HRMS calcd for $C_{25}H_{24}O_7$ (*M*): *M+Na*, 459.1414. Found: *m/z* 459.1395.

1.4. General syntethic procedure and spectroscopic data for trimethyl 7-hydroxy-11-aryl-9,10-dihydro-5,9-methanobenzo[8]annulene-6,8,8(5*H*)-tricarboxylates (3)

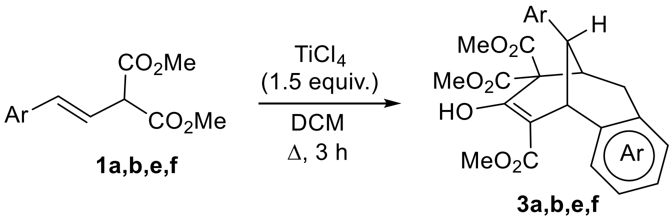

All operations were performed under dry argon atmosphere. A solution of styrylmalonate **1a,b,e** (0.34 mmol, 1 equiv.) and $TiCl_4$ (0.51 mmol, 1.5 equiv.) in dry DCM (3 mL) was stirred at reflux for 3 hours. Then an aqueous solution of HCl (10%) was added at room temperature and the reaction mixture was extracted with CH_2Cl_2 (3×10 mL). The organic layer washed the brine and dried over $MgSO_4$. The solvent was removed *in vacuo*. The residue was purified by column chromatography on silica gel (benzene to benzene–EtOAc, 10:1) to afford title compounds **3a,b,e**. The compound **3f** was obtained and isolated as by-product from reaction of styrylmalonate **1f** in the presence of $TiCl_4$ (0.5 equiv.) in dry DCE (3 mL) at reflux as described in the section 1.2 under synthsis of compound **2f**.

The resulting compounds can be additionally purified on a Silufol chromatographic plate (20×20 cm) using hexane–acetone (2:1) eluent to afford the pure products.

Trimethyl 7-hydroxy-11-phenyl-9,10-dihydro-5,9-methanobenzo[8]annulene-6,8,8(5H)-tricarboxylate (3a)

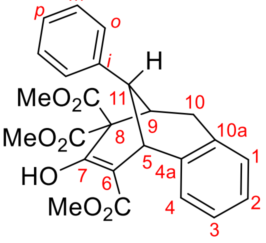

The title compound was prepared in yield 22 mg (30%). Colorless thick oil. IR ($CHCl_3$): $\tilde{\nu}$ 3180 (O-H), 3053, 3027, 3015, 1737 br (C=O), 1659, 1620, 1496, 1437, 1357, 1249, 1183 cm^{-1}.

^{1}H NMR (300.1 MHz, $CDCl_3$): δ 3.04 (s, 3H, CO_2Me), 3.10 (d, 1H, H_a(10), ^2J = 18.5 Hz), 3.44 (dd, 1H, H_b(10), ^2J = 18.5 Hz, ^3J = 7.4 Hz), 3.58 (br.s, 1H, H(11)), 3.69 and 3.99 (both s, 2 × 3H, 2 CO_2Me), 3.74–3.90 (m, 1H, H(9)), 4.30 (br.s, 1H, H(5)), 6.95–7.48 (m, 9H, 9 H_{Ar}), 12.43 (s, 1H, OH) ppm. ^{13}C NMR (75.5 MHz, $CDCl_3$): δ 34.7 (C(5)), 34.9 (C(10)), 41.0 (C(9)), 42.7 (C(11)), 52.2, 52.6 and 52.7 (3 OMe), 62.5 (C(8)), 105.4 (C(6)), 125.6, 126.6, 126.8 and 127.1 (4 CH_{Ar}), 128.0 (2 C(*m*)), 128.3 (2

C(*o*)), 128.6 (C(*p*)), 133.1 and 141.3 (C(4a) and C(10a)), 139.8 (C(*i*)), 165.6 (C(7)), 167.2, 168.2 and 171.8 (3 COO). MS (*m/z*, %): 436 (11, M^+), 404 (29), 372 (11), 340 (8), 331 (6), 313 (11), 299 (8), 285 (25), 272 (11), 257 (9), 244 (20), 229 (17), 215 (38), 204 (100), 189 (11), 172 (24), 152 (17), 142 (18), 133 (28), 115 (78), 105 (39), 91 (97), 77 (57), 59 (92). HRMS calcd for $C_{25}H_{24}O_7$ (*M*): *M+H*, 437.1595; *M+Na*, 459.1414. Found: *m/z* 437.1600, 459.1417.

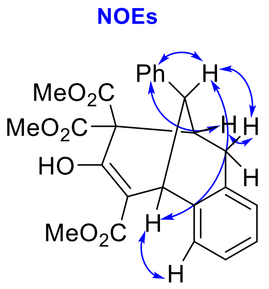

2D ^{1}H,^{1}H-NOESY NMR data:

Trimethyl 2-bromo-11-(3-bromophenyl)-7-hydroxy-9,10-dihydro-5,9-methanobenzo[8]-annulene-6,8,8(5H)-tricarboxylate (3b)

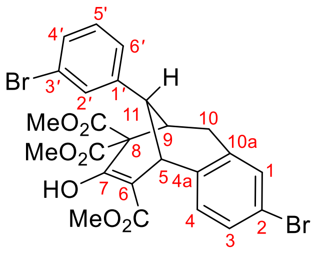

The title compound was prepared in yield 37 mg (37%). Colorless thick oil. IR ($CHCl_3$): $\tilde{\nu}$ 3179 (O-H), 3050, 3022, 3015, 1738 br (C=O), 1660, 1569, 1477, 1437, 1251, 1182 cm^{-1}.

^{1}H NMR (300.1 MHz, $CDCl_3$): δ 3.11 (d, 1H, H_a(10), ^2J = 18.7 Hz), 3.17 (s, 3H, CO_2Me), 3.38 (dd, 1H, H_b(10), ^2J = 18.7 Hz, ^3J = 7.0 Hz), 3.50 (br.s, 1H, H(11)), 3.72 and 3.99 (both s, 2 × 3H, 2 CO_2Me), 3.77–3.89 (m, 1H, H(9)), 4.22 (br.s, 1H, H(5)), 6.92–7.65 (m, 7H, 7 H_{Ar}), 12.43 (s, 1H, OH) ppm. ^{13}C NMR (75.5 MHz, $CDCl_3$): δ 34.2 (C(5)), 34.5 (C(10)), 40.2 (C(9)), 42.4 (C(11)), 52.3, 52.8 and 52.9 (3 OMe), 62.4 (C(8)), 104.6 (C(6)), 120.4 and 122.1 (2 C-Br), 126.7, 128.7, 128.8, 129.6, 129.8, 131.5 and 131.6 (7 CH_{Ar}), 135.3 and 141.9 (C(4a) and C(10a)), 140.0 (C(1´)), 165.7 (C(7)), 166.9, 167.8 and 171.4 (3 COO). MS (*m/z* for ^{79}Br, %): 560 (2, M^+), 490 (2), 441 (3), 428 (1), 400 (1), 373 (1), 360 (38), 312 (13), 298 (24), 280 (16), 270 (13), 251 (12), 232 (23), 215 (47), 200 (46), 185 (40), 172 (44), 144 (26), 133 (44), 115 (85), 101 (44), 87 (100), 69 (51), 55 (62). HRMS calcd for $C_{25}H_{22}Br_2O_7$ (*M*, for ^{79}Br): *M+H*, 592.9805; *M+Na*, 614.9624. Found: *m/z* 592.9792, 614.9606.

Trimethyl 3-fluoro-11-(4-fluorophenyl)-7-hydroxy-9,10-dihydro-5,9-methanobenzo[8]-annulene-6,8,8(5H)-tricarboxylate (3e)

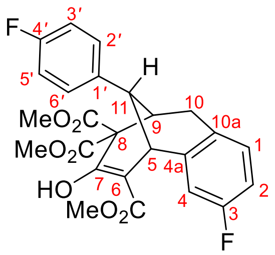

The title compound was identified in about 10% NMR yield. ^{1}H NMR (300.1 MHz, $CDCl_3$): 4.23 (br.s, 1H, H(5)), 12.60 (s, 1H, OH) ppm. Other signals are very small or overlap with the signals of other products and cannot be exactly identified.

Trimethyl 7-hydroxy-3-methyl-11-(p-tolyl)-9,10-dihydro-5,9-methanobenzo[8]annulene-6,8,8(5H)-tricarboxylate (3f)

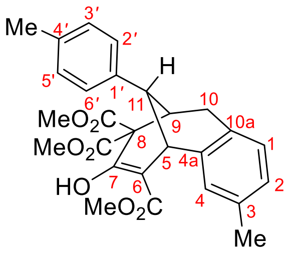

The compound **3f** was isolated from reaction mixture under preparation of cyclohexadienol **2f** in yield 6.5 mg (9%). Light yellow thick oil. IR ($CHCl_3$): $\tilde{\nu}$ 3160 (O-H), 3050, 3002, 2954, 2926, 1735 br (C=O), 1658, 1616, 1515, 1437, 1357, 1248, 1182 cm^{-1}.

^{1}H NMR (300.1 MHz, $CDCl_3$): δ 2.29 (s, 3H, Me), 2.30 (s, 3H, Me), 3.04 (d, 1H, H_a(10), ^2J = 18.7 Hz), 3.05 (s, 3H, CO_2Me), 3.37 (dd, 1H, H_b(10), ^2J = 18.7 Hz, ^3J = 7.7 Hz), 3.50–3.58 (m, 1H, H(11)), 3.68 and 3.99 (both s, 2 × 3H, 2 CO_2Me), 3.79–3.90 (m, 1H, H(9)), 4.23 (br.s, 1H, H(5)), 5.98–7.81 (m, 7H, 7 H_{Ar}), 12.40 (s, 1H, OH) ppm. ^{13}C NMR (75.5 MHz, $CDCl_3$): δ 20.9 and 21.1 (2 Me), 34.6 and 34.7 (C(5) and C(10)), 41.2 (C(9)), 42.4 (C(11)), 51.7, 52.3 and 52.4 (3 OMe), 62.6 (C(8)), 105.5 (C(6)), 128.2 (C(2´) and C(6´)), 128.4, 128.7 and 128.9 (3 CH_{Ar}), 128.6 (C(3´) and C(5´)), 135.0 and 141.2 (C(4a) and C(10a)), 135.9 and 136.7 (C(3) and C(4´)), 137.4 (C(1´)), 165.7 (C(7)), 168.2, 170.8 and 171.9 (3 COO). MS (*m/z*, %): 464 (10, M^+), 432 (12), 400 (6), 364 (100), 345 (6), 313 (12), 304 (34), 285 (6), 272 (14), 257 (6), 245 (16), 232 (70), 215 (14), 202 (6), 185 (16), 159 (6), 145 (7), 128 (40), 115 (24), 105 (30), 83 (48), 59 (62), 41 (30), 15 (62). HRMS calcd for $C_{27}H_{28}O_7$ (*M*): *M+H*, 465.1908; *M+Na*, 487.1727. Found: *m/z* 465.1899, 487.1718.

2. References

[S1] A. O. Chagarovskiy, O. A. Ivanova, E. R. Rakhmankulov, E. M. Budynina, I. V. Trushkov, M. Y. Melnikov, *Adv. Synth. Catal.* **2010**, *352*, 3179.

[S2] D. D. Borisov, G. R. Chermashentsev, R. A. Novikov, Y. V. Tomilov, *Synthesis* **2021**, *53*, 2253.

3. NMR spectra for the new compounds:

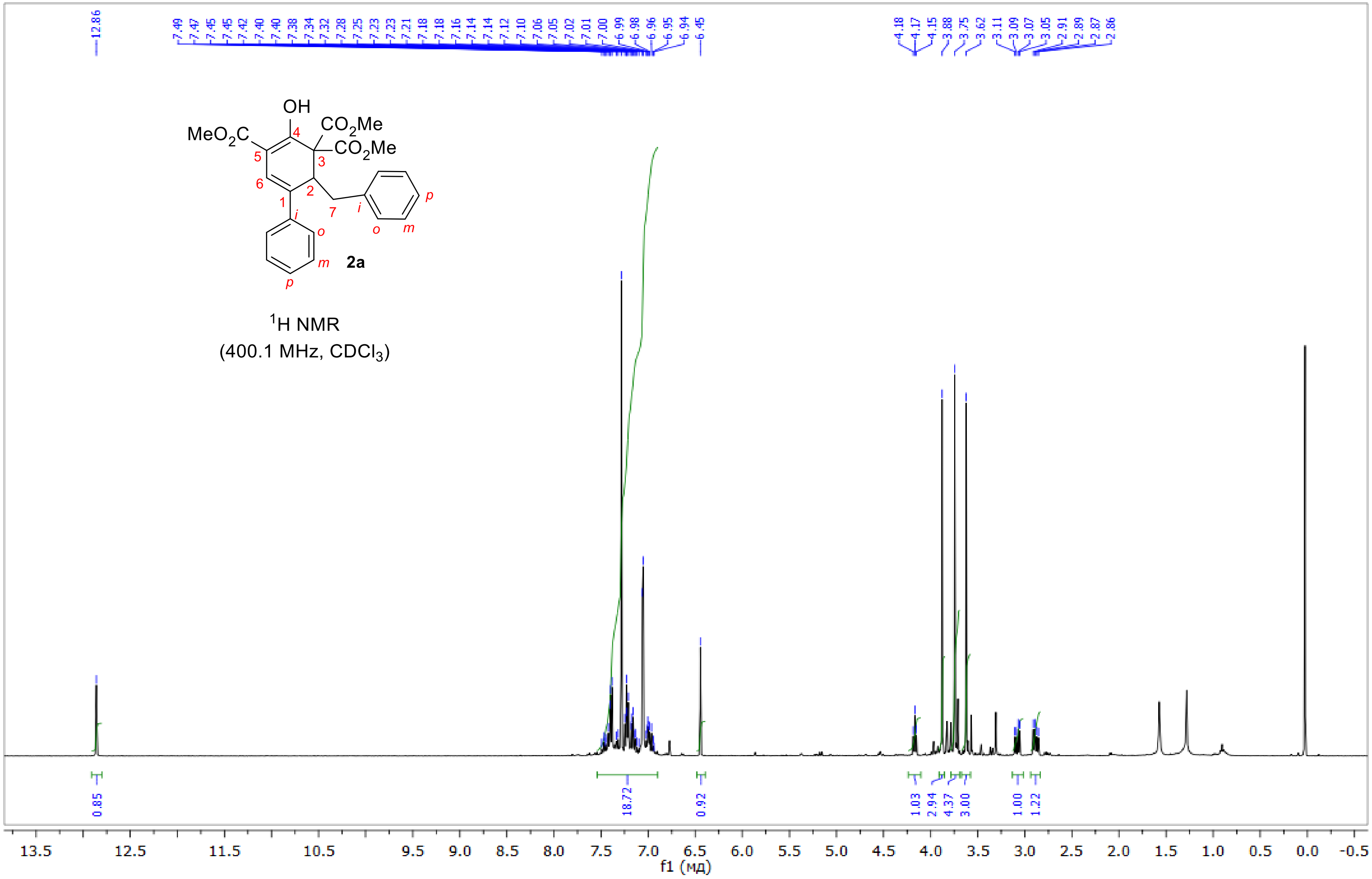

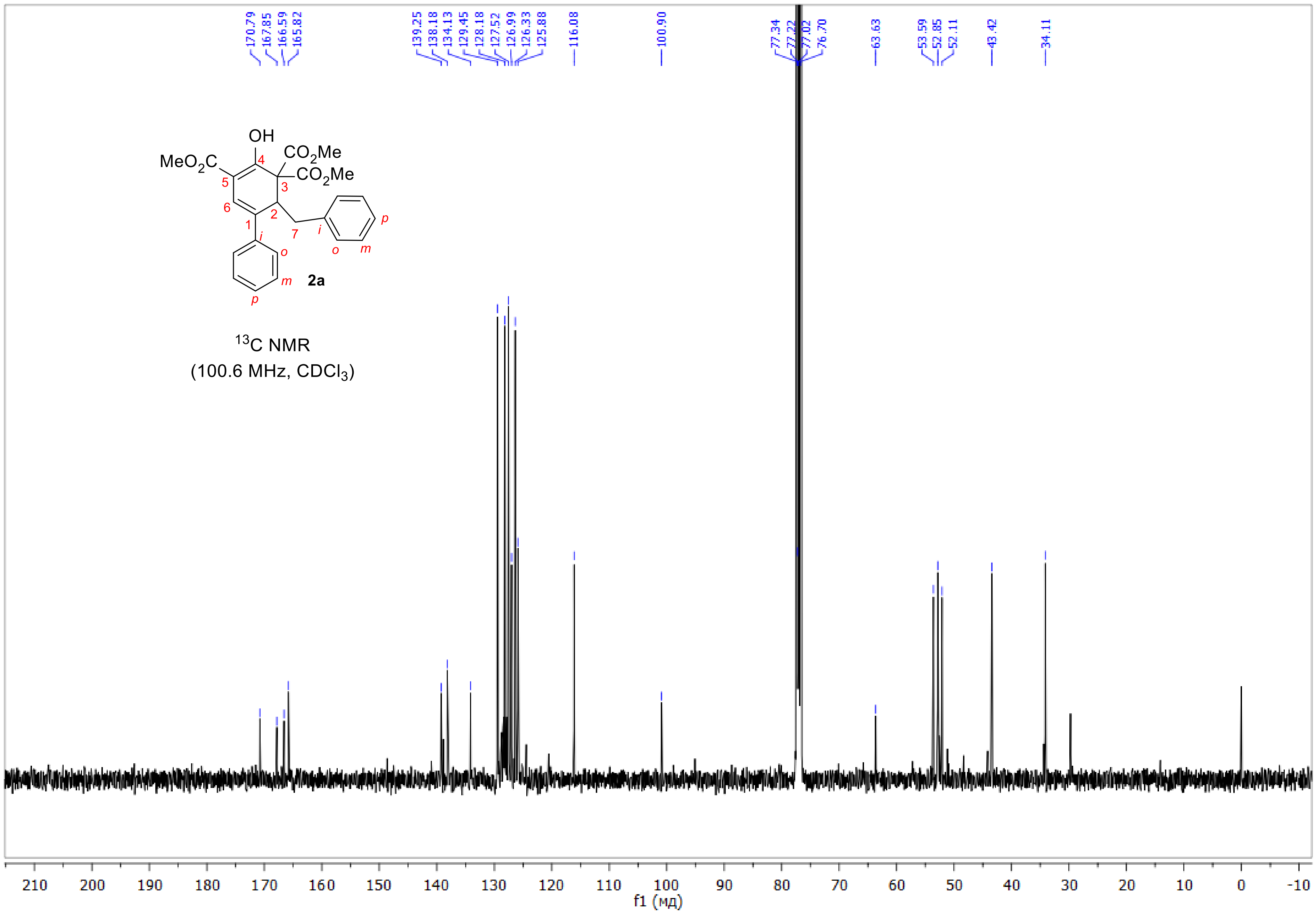

170.79
167.85
166.59
165.82
139.25
138.18
134.13
129.45
128.18
127.52
126.99
126.33
125.88
116.08
100.90
77.34
77.22
77.02
76.70
63.63
53.59
52.85
52.11
43.42
34.11
OH
MeO_2C
CO_2Me
CO_2Me
2a
^{13}C NMR
(100.6 MHz, $CDCl_3$)
f1 (мд)

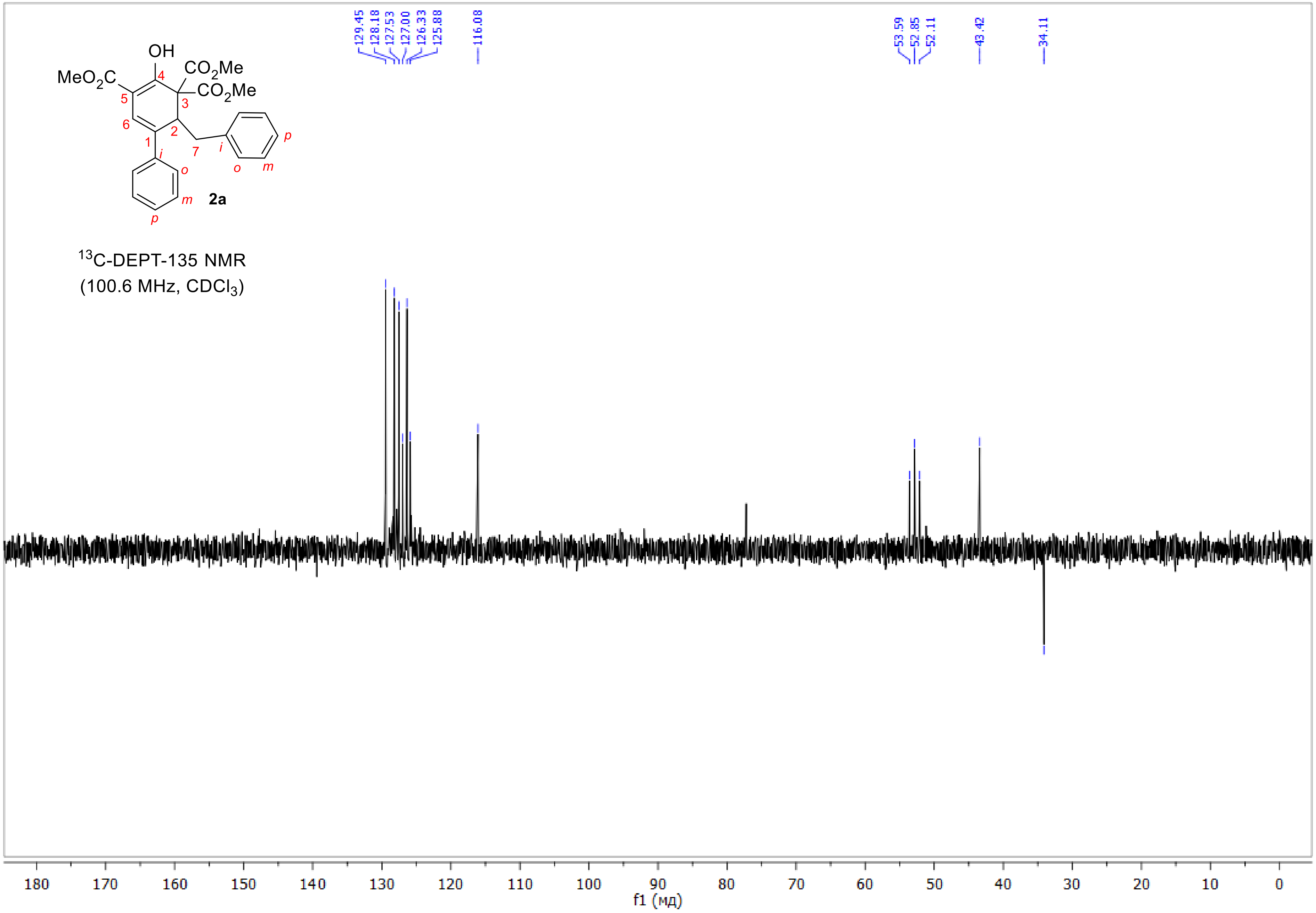

OH
MeO2C
CO2Me
CO2Me
2a
13C-DEPT-135 NMR
(100.6 MHz, CDCl3)
129.45
128.18
127.53
127.00
126.33
125.88
116.08
53.59
52.85
52.11
43.42
34.11
180
170
160
150
140
130
120
110
100
90
80
70
60
50
40
30
20
10
0
f1 (мд)

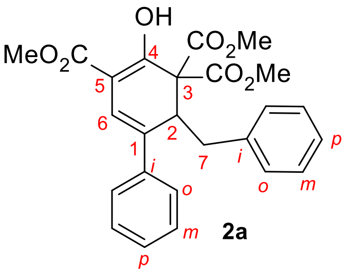

2a

$^1H,^1H$-COSY
(400.1 MHz, $CDCl_3$)

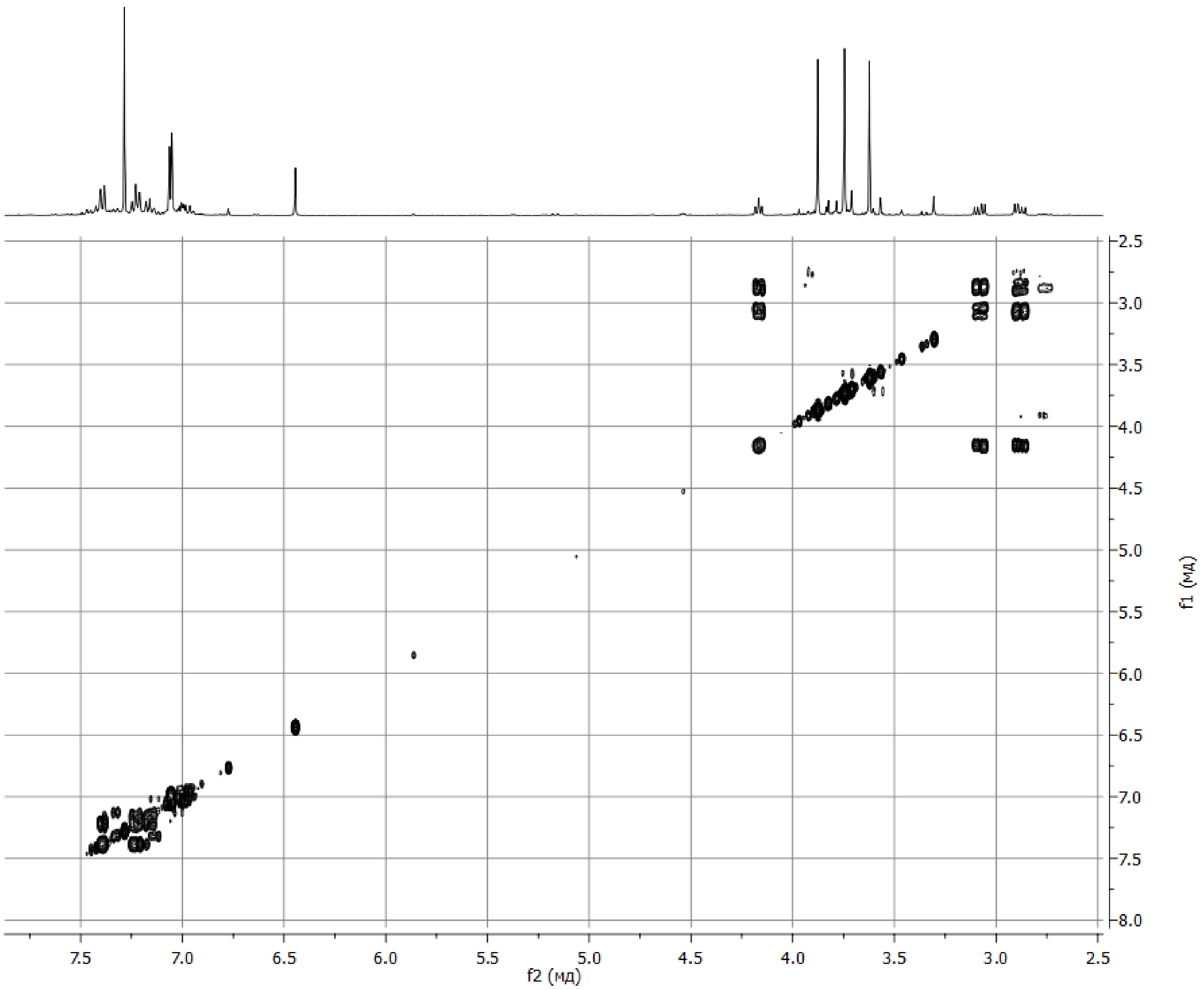

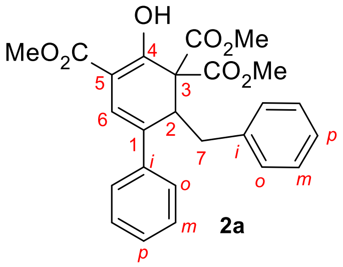

$^{1}H,^{1}H$-NOESY
(400.1 MHz, $CDCl_3$)

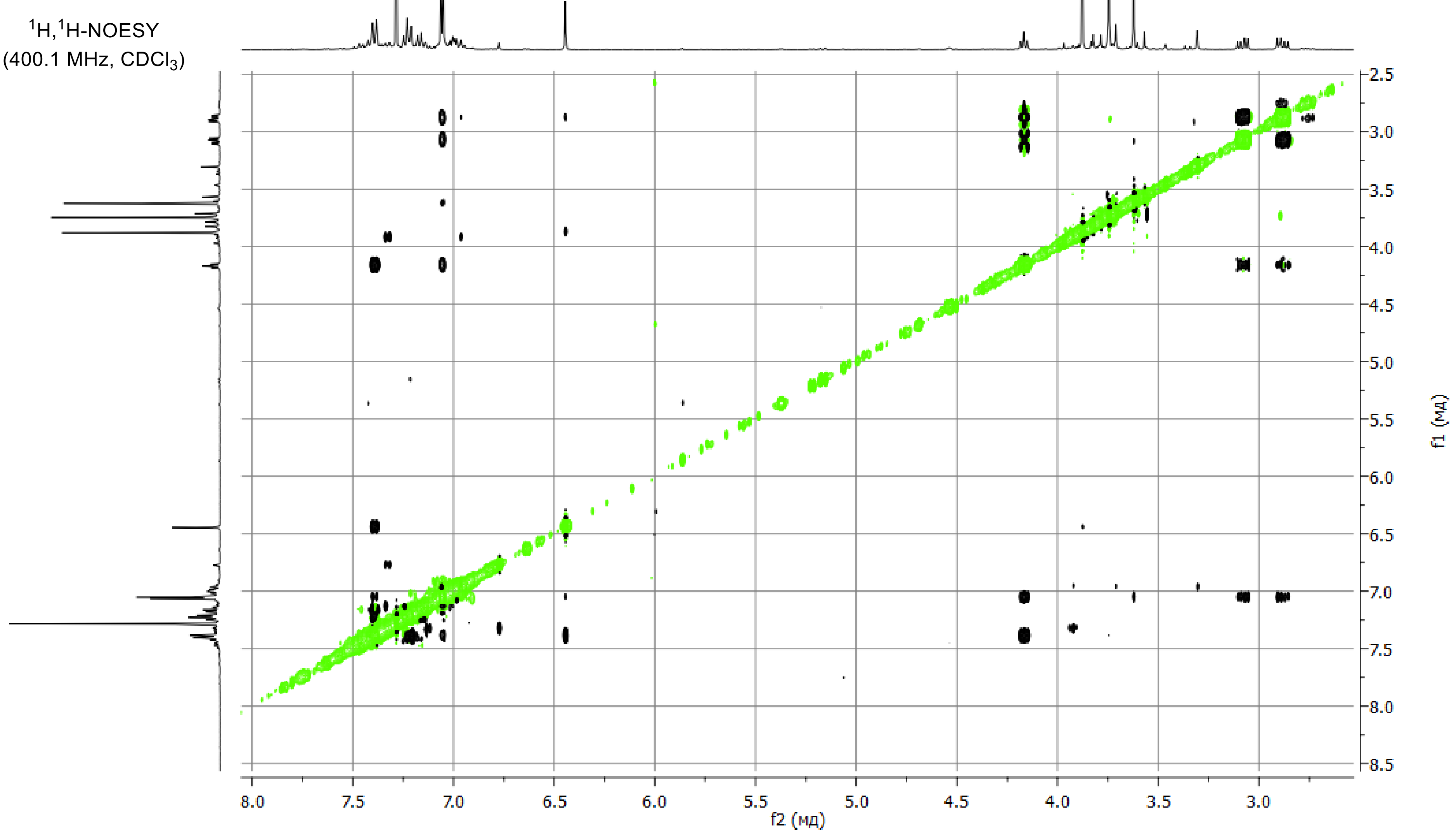

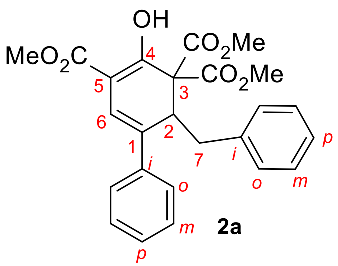

2a

$^{1}H,^{13}C$-HSQC
(^{1}H: 400.1 MHz,
^{13}C: 100.6 MHz
$CDCl_3$)

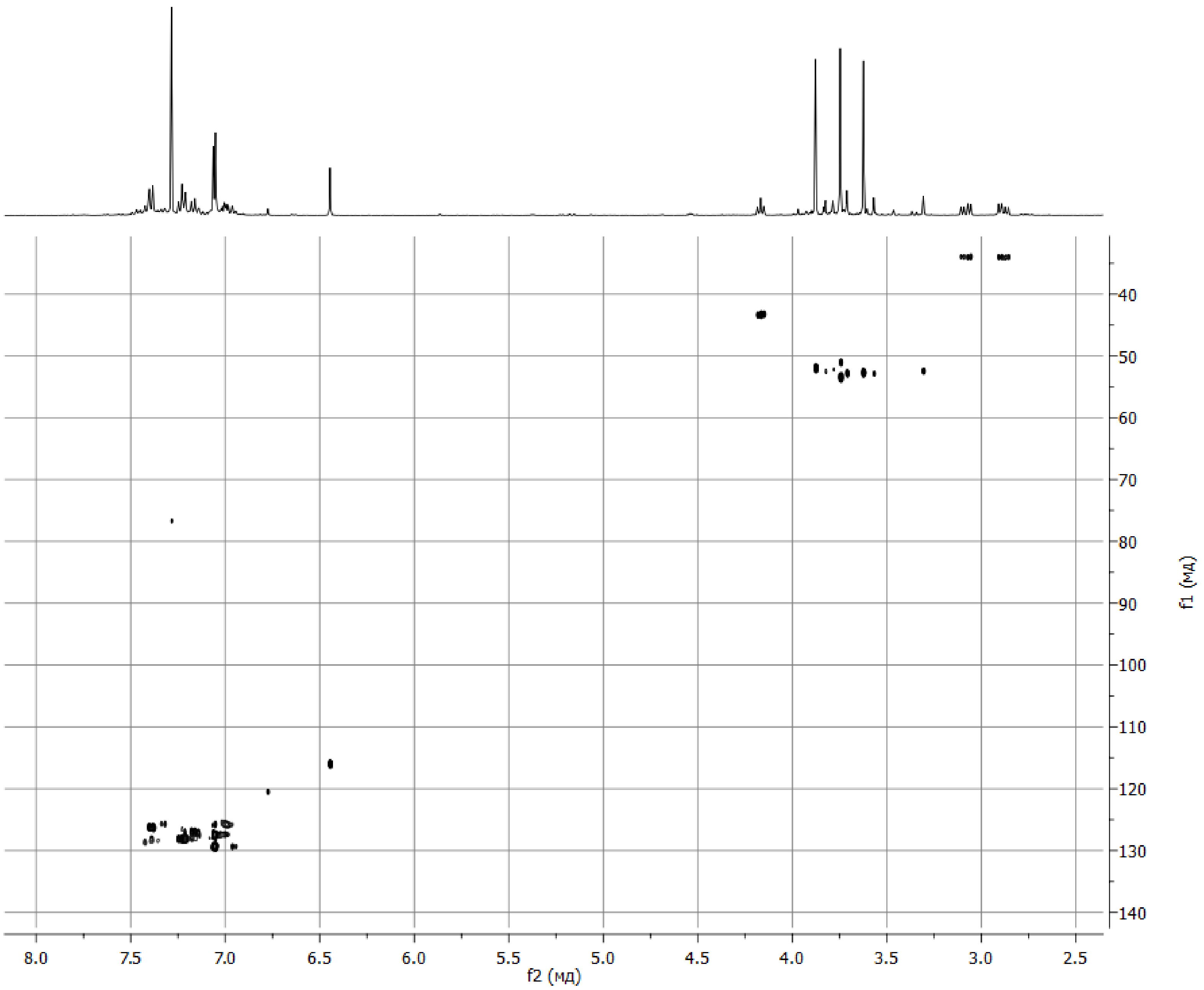

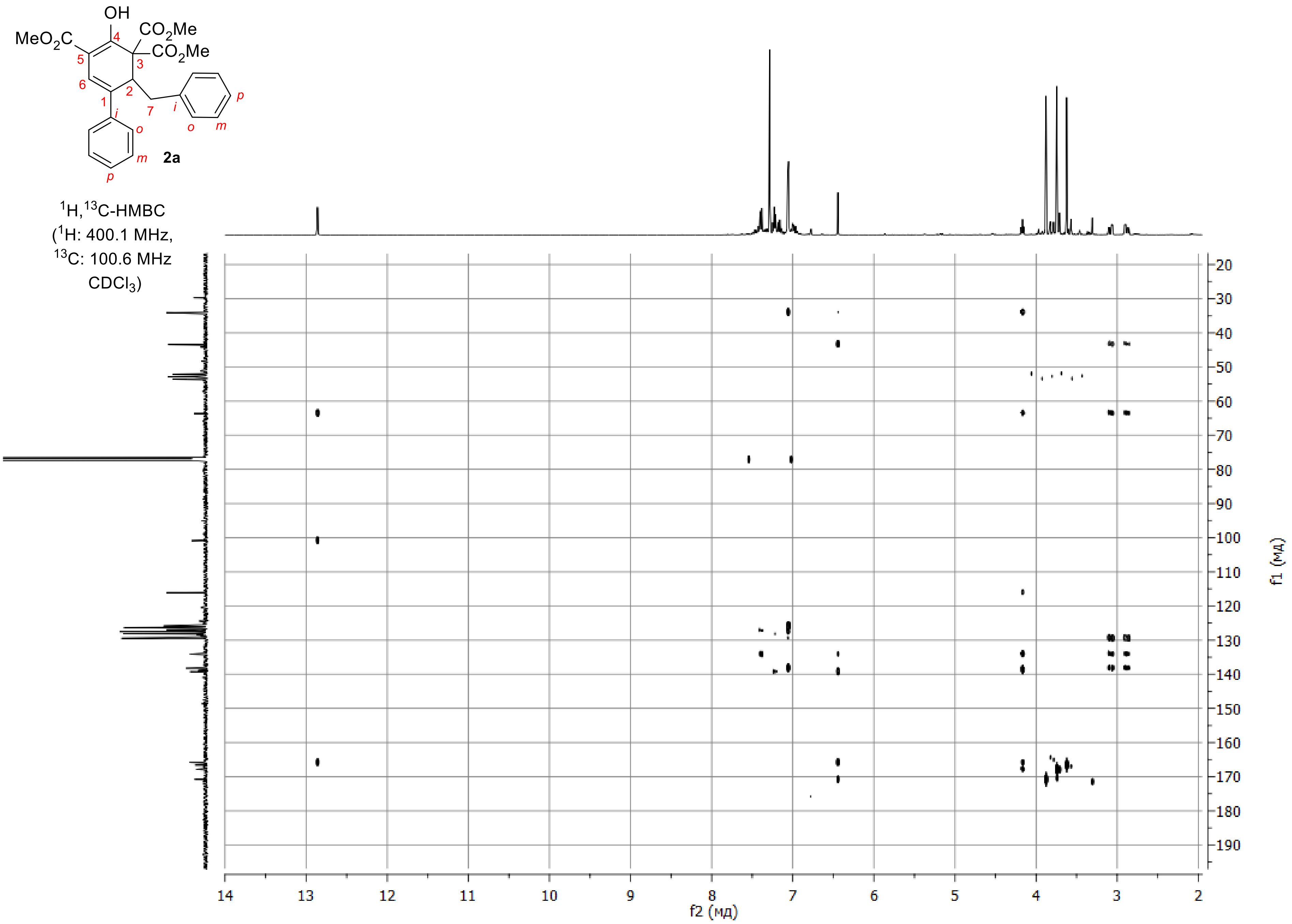

$^{1}H,^{13}C$-HMBC
(^{1}H: 400.1 MHz,
^{13}C: 100.6 MHz
$CDCl_3$)

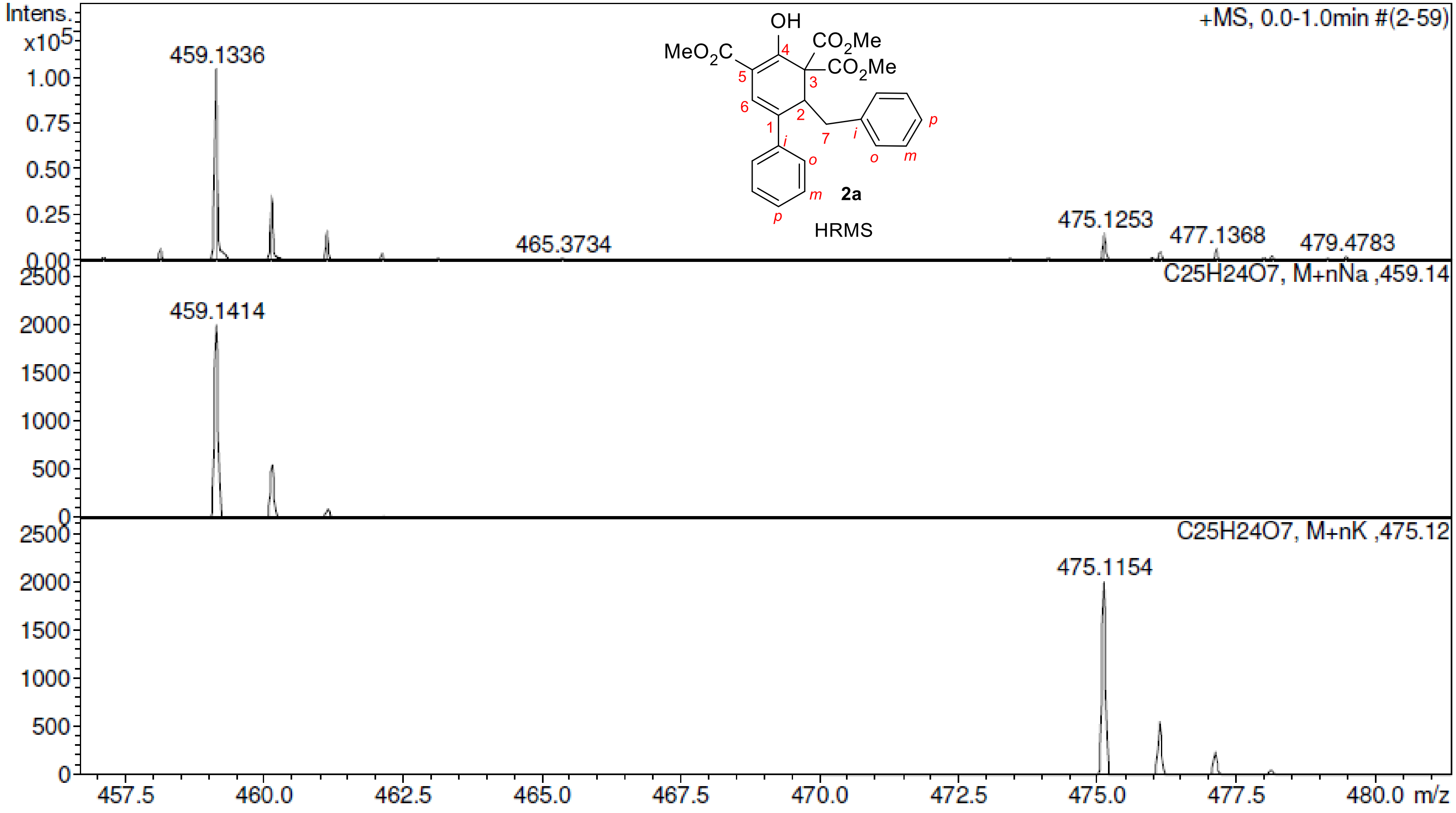

Intens.
x10^5
1.00
0.75
0.50
0.25
0.00
459.1336
465.3734
475.1253
477.1368
479.4783
+MS, 0.0-1.0min #(2-59)
OH
MeO2C
CO2Me
CO2Me
2a
HRMS
2500
2000
1500
1000
500
0
459.1414
C25H24O7, M+nNa ,459.14
475.1154
C25H24O7, M+nK ,475.12
457.5
460.0
462.5
465.0
467.5
470.0
472.5
475.0
477.5
480.0 m/z

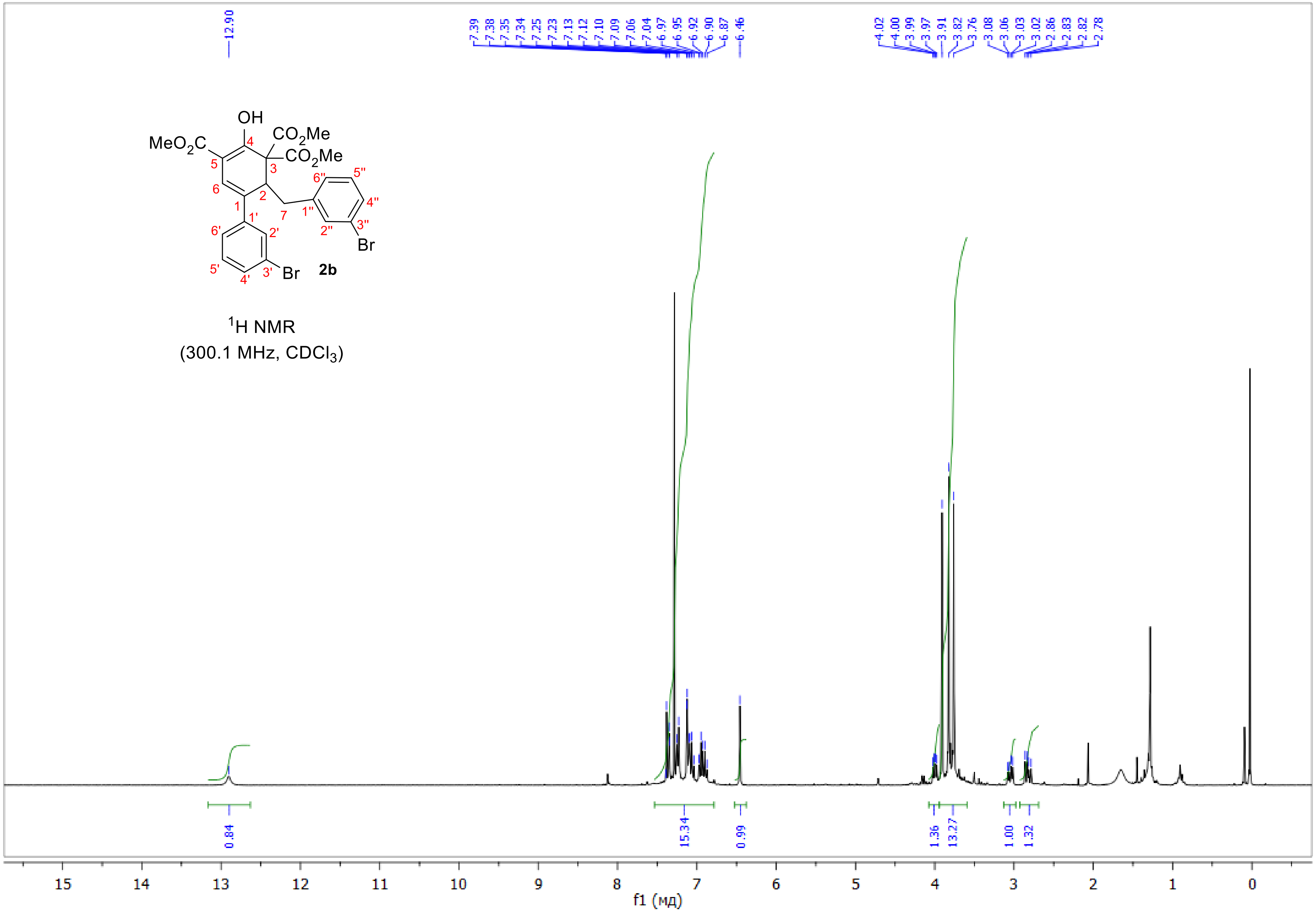

OH
MeO2C
CO2Me
CO2Me
Br
Br
2b
1H NMR
(300.1 MHz, CDCl3)
f1 (мд)

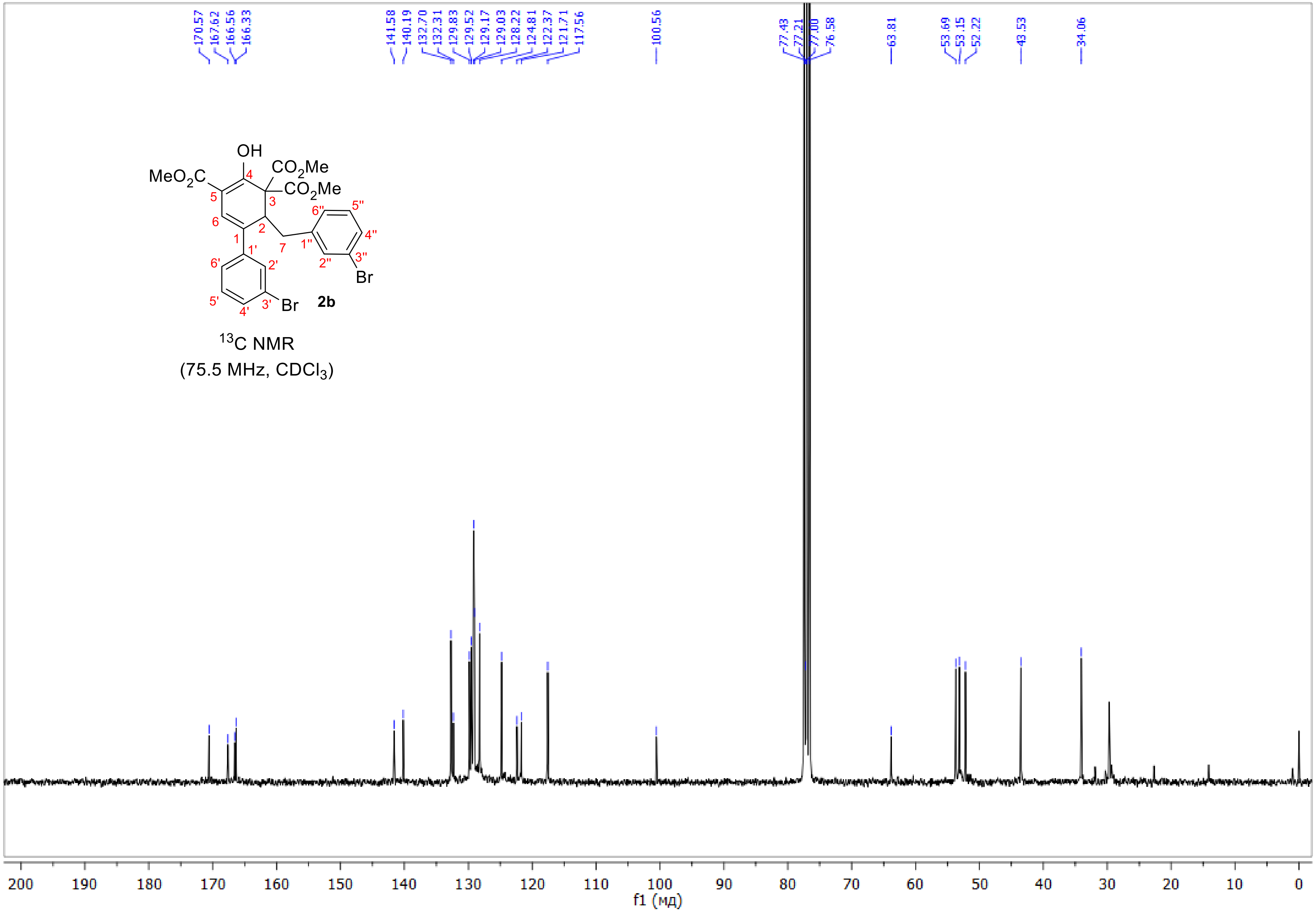
170.57
167.62
166.56
166.33
141.58
140.19
132.70
132.31
129.83
129.52
129.17
129.03
128.22
124.81
122.37
121.71
117.56
100.56
77.43
77.21
77.00
76.58
63.81
53.69
53.15
52.22
43.53
34.06
OH
MeO2C
CO2Me
CO2Me
Br
Br
2b
13C NMR
(75.5 MHz, CDCl3)
200 190 180 170 160 150 140 130 120 110 100 90 80 70 60 50 40 30 20 10 0
f1 (мд)

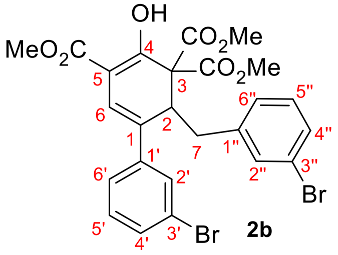

2b

$^{1}H,^{1}H$-COSY

(300.1 MHz, $CDCl_3$)

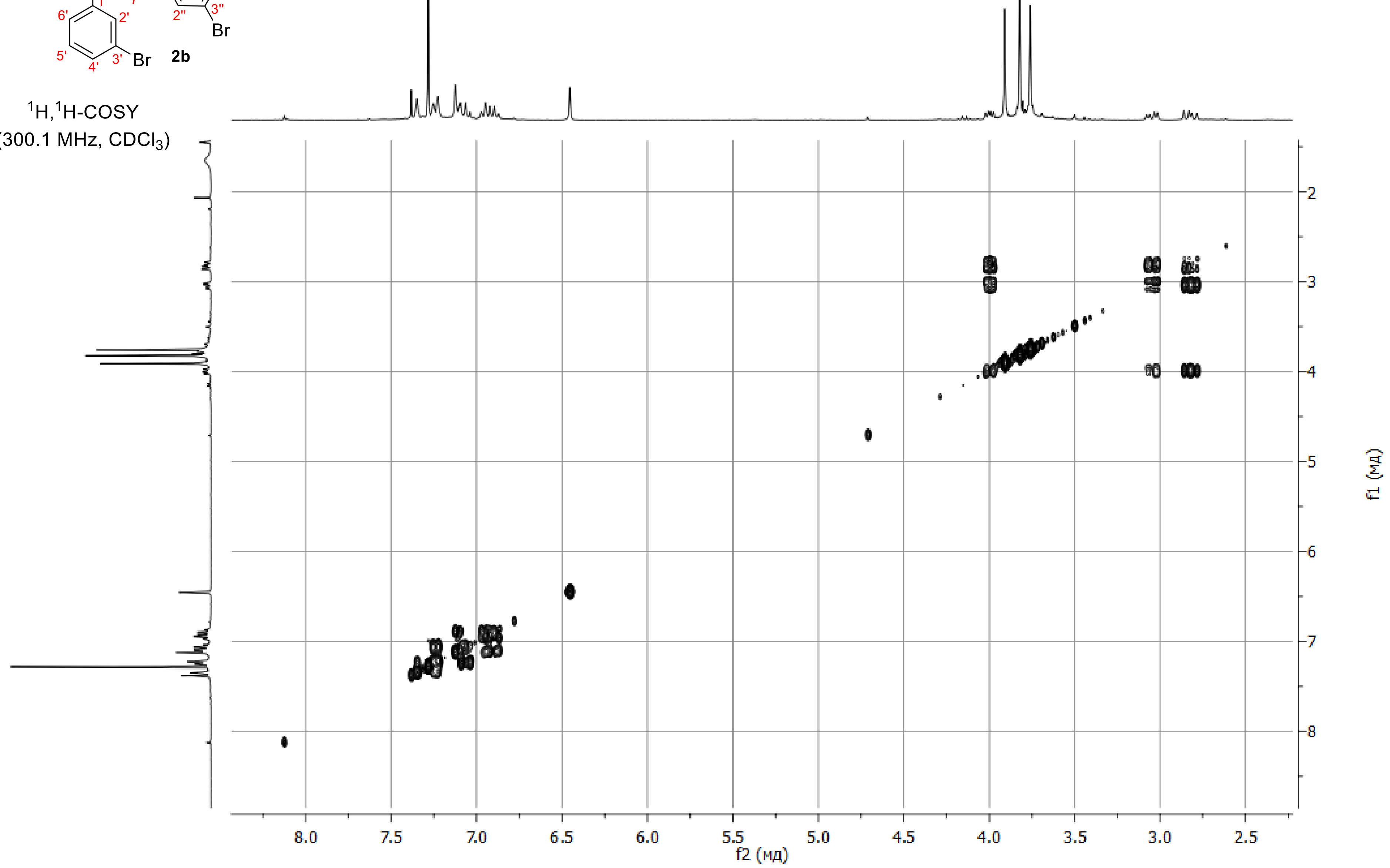

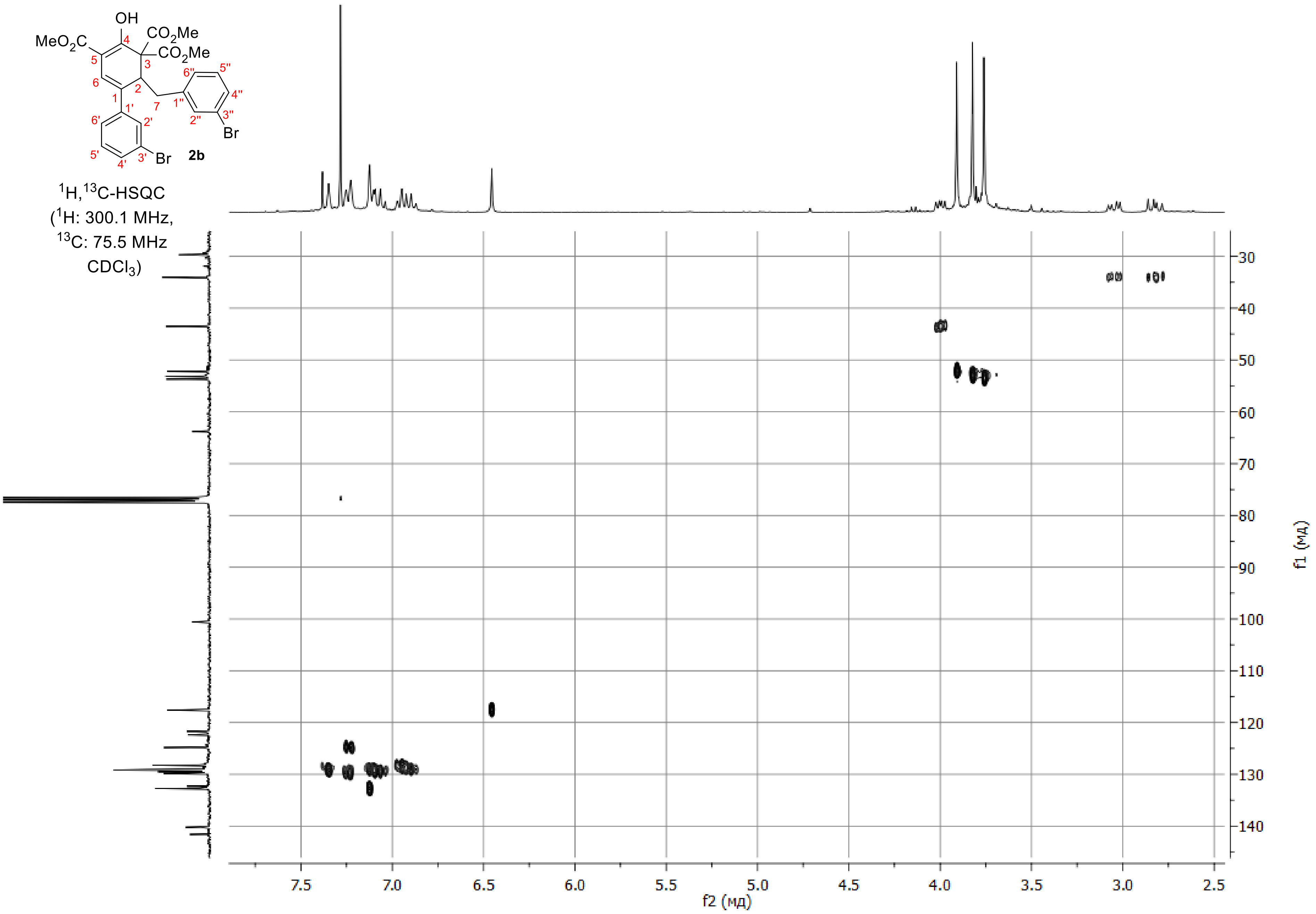

2b

$^{1}H,^{13}C$-HSQC
(^{1}H: 300.1 MHz,
^{13}C: 75.5 MHz
$CDCl_3$)

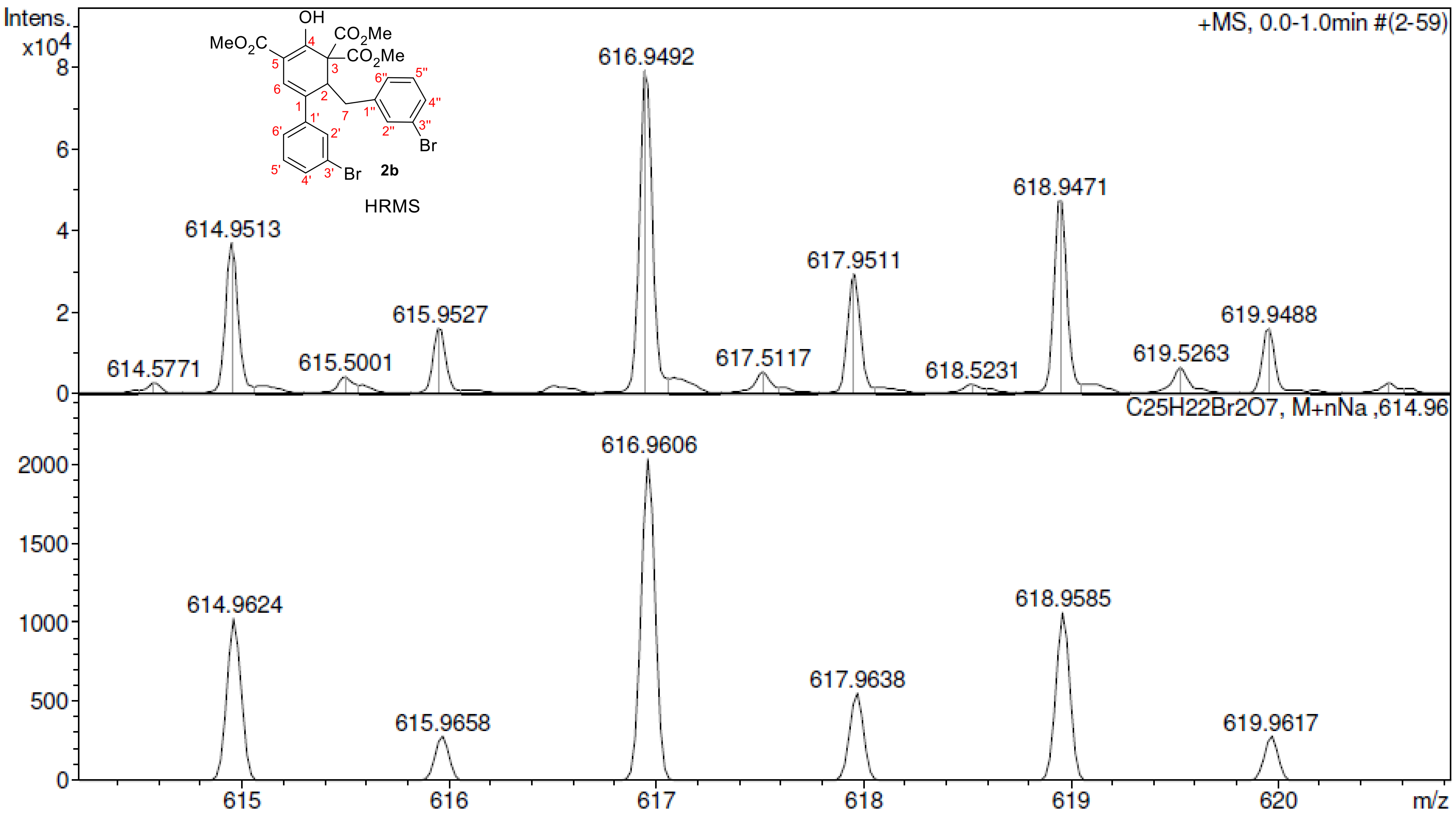

Intens.
x10^4
+MS, 0.0-1.0min #(2-59)
2b
HRMS
614.5771
614.9513
615.5001
615.9527
616.9492
617.5117
617.9511
618.5231
618.9471
619.5263
619.9488
C25H22Br2O7, M+nNa ,614.96
614.9624
615.9658
616.9606
617.9638
618.9585
619.9617
m/z

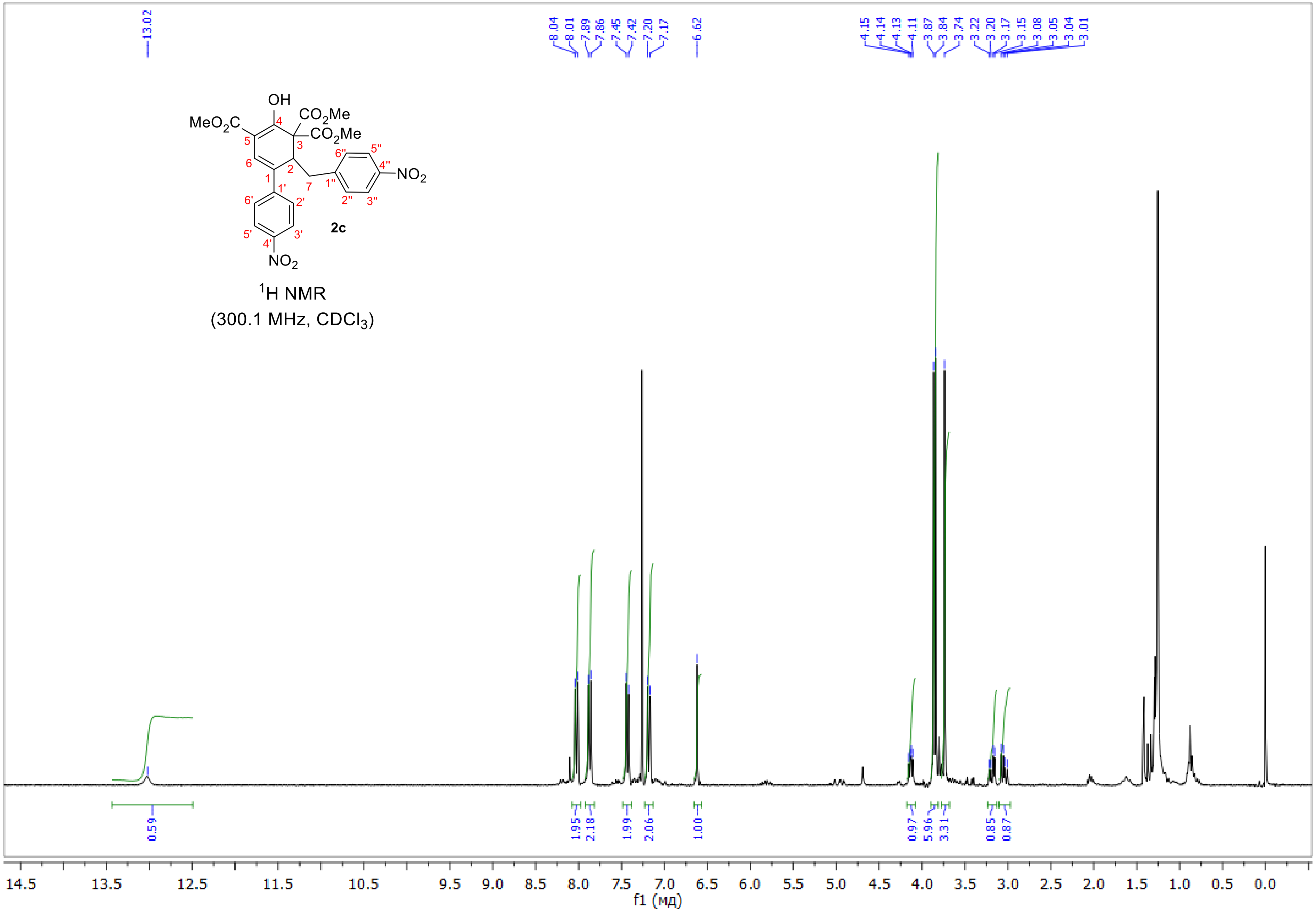

2c
^{1}H NMR
(300.1 MHz, CDCl$_3$)
f1 (мд)

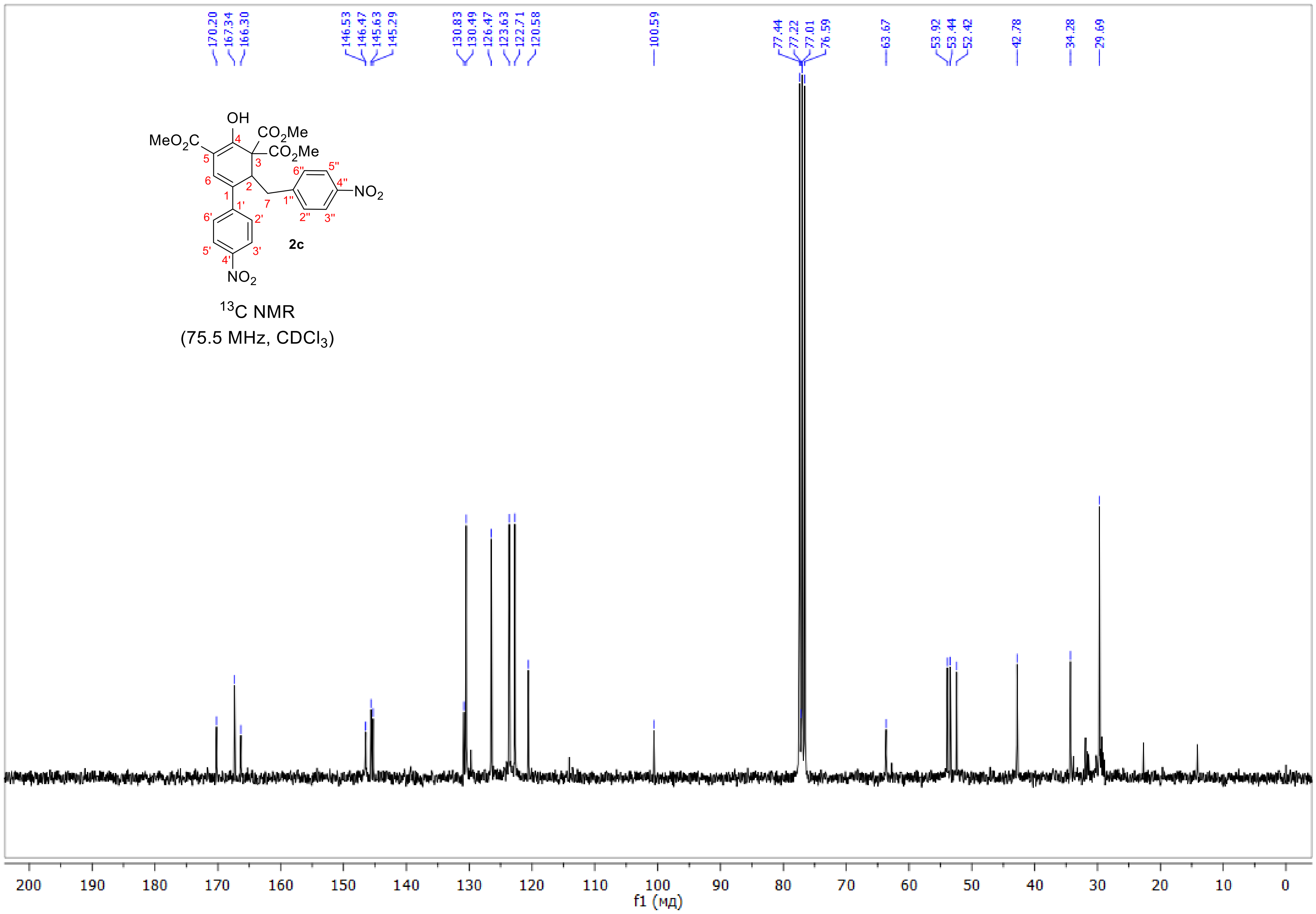
170.20
167.34
166.30
146.53
146.47
145.63
145.29
130.83
130.49
126.47
123.63
122.71
120.58
100.59
77.44
77.22
77.01
76.59
63.67
53.92
53.44
52.42
42.78
34.28
29.69
OH
MeO2C
CO2Me
CO2Me
NO2
NO2
2c
13C NMR
(75.5 MHz, CDCl3)
f1 (мд)

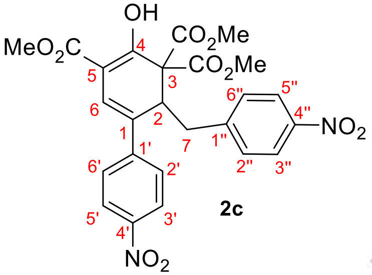

2c

^{1}H,^{1}H-COSY
(300.1 MHz, $CDCl_3$)

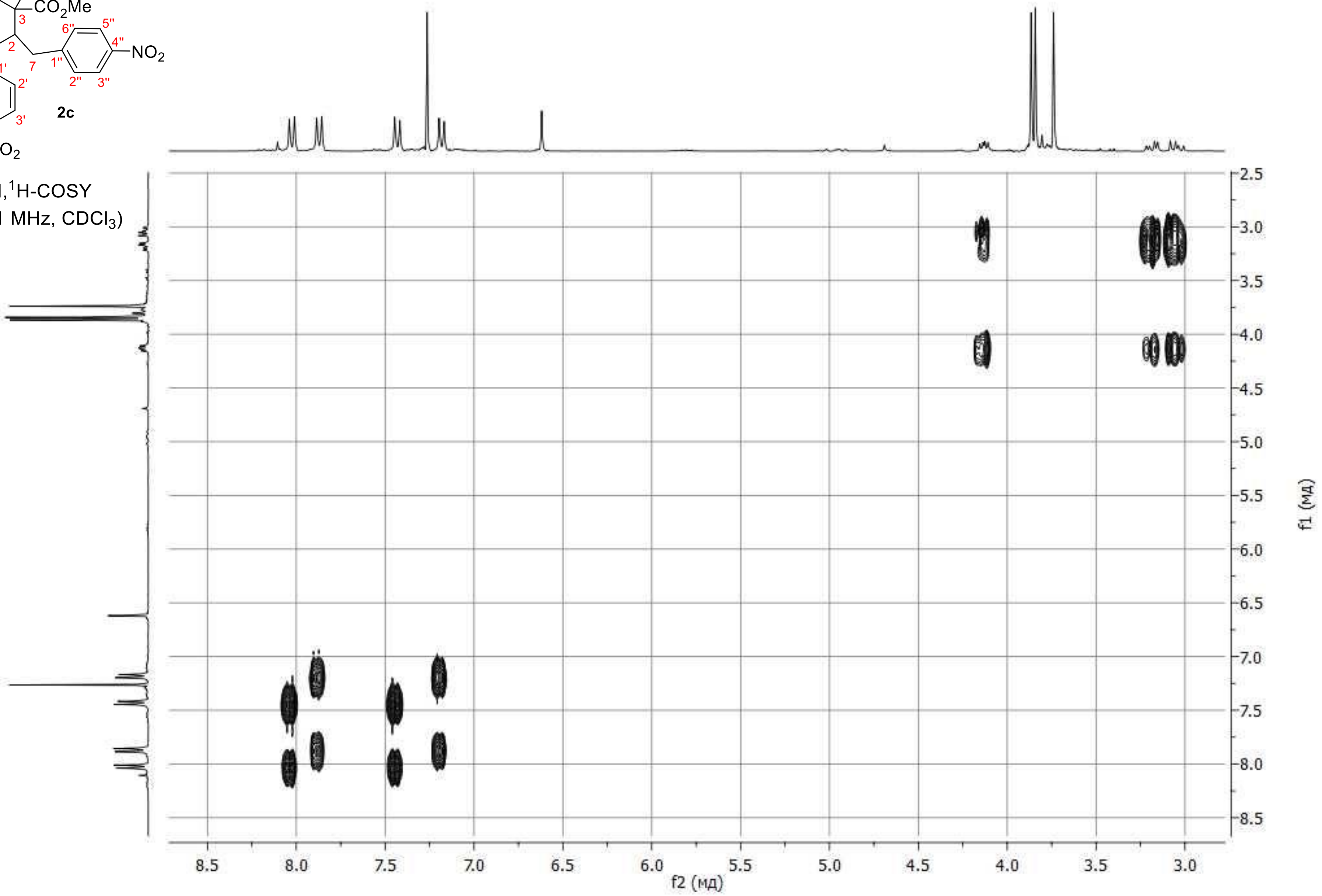

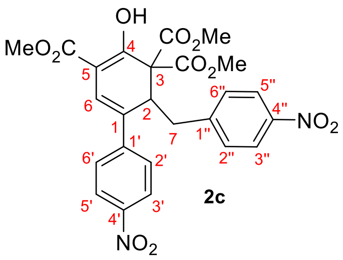

2c

$^{1}H,^{13}C$-HSQC
(^{1}H: 300.1 MHz,
^{13}C: 75.5 MHz
$CDCl_3$)

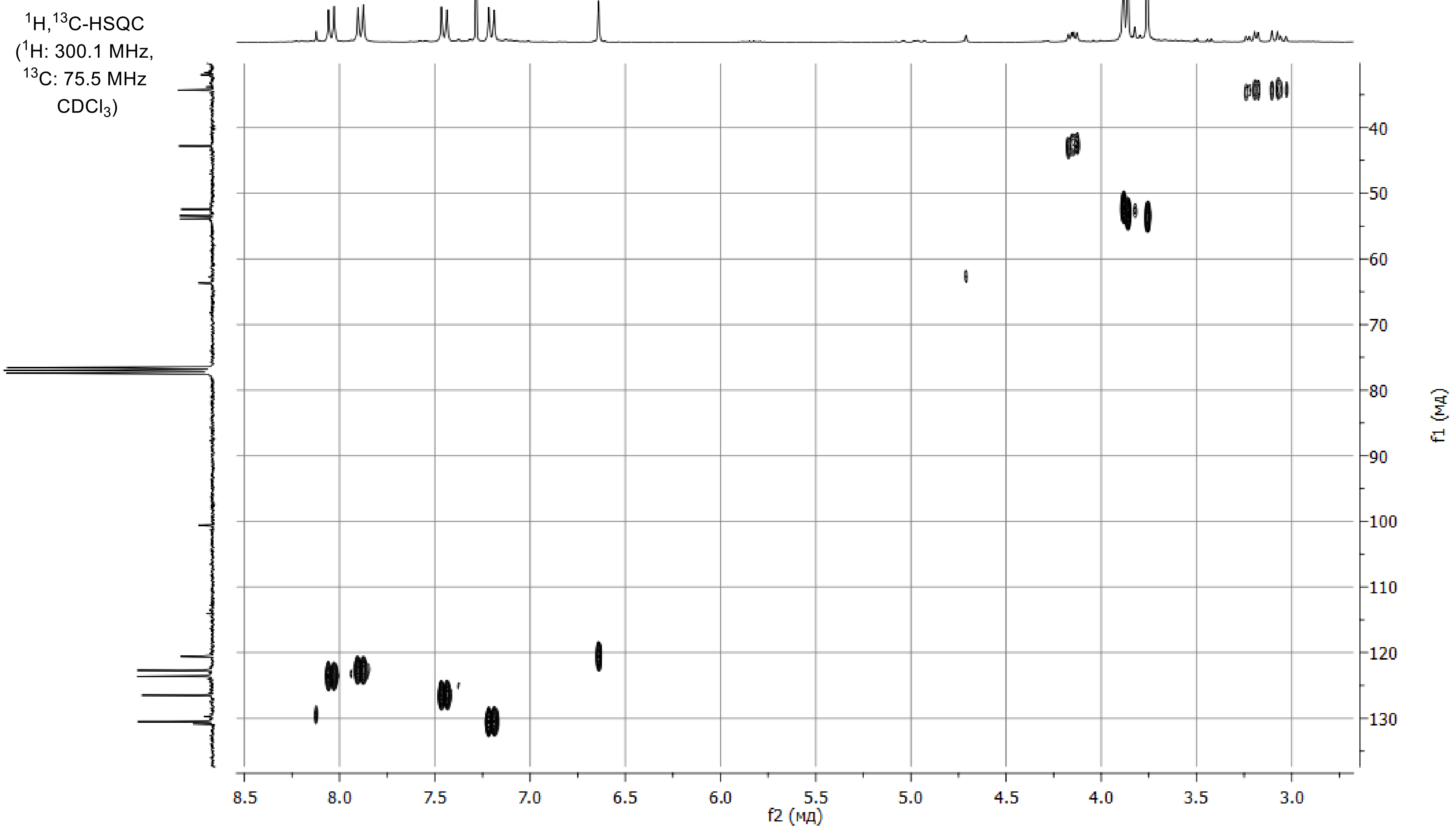

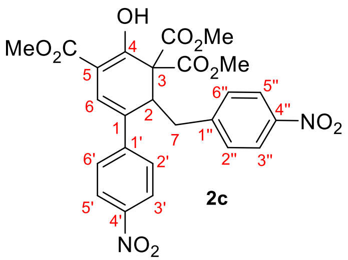

$^1H,^{13}C$-HMBC
(^1H: 300.1 MHz,
^{13}C: 75.5 MHz
$CDCl_3$)

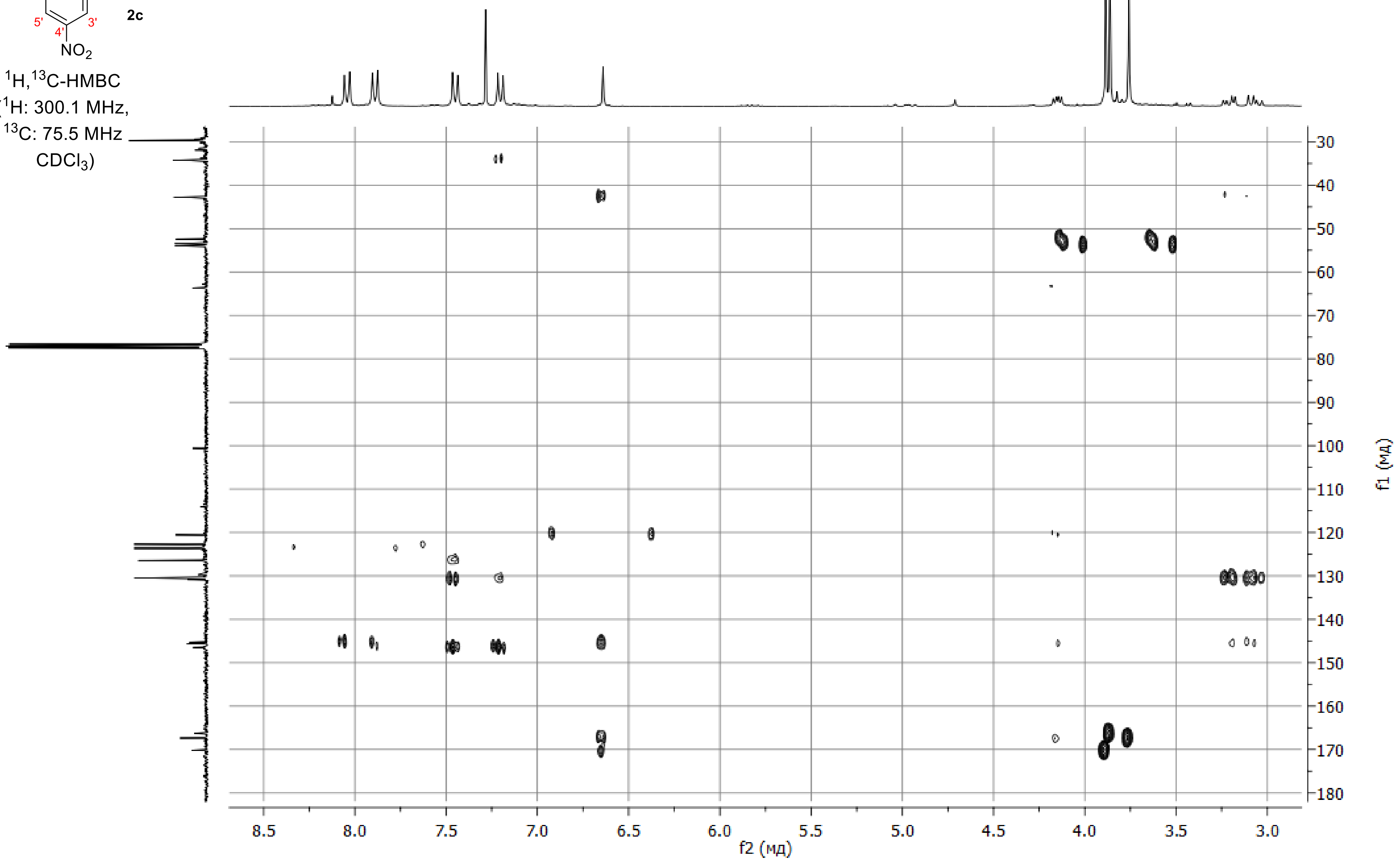

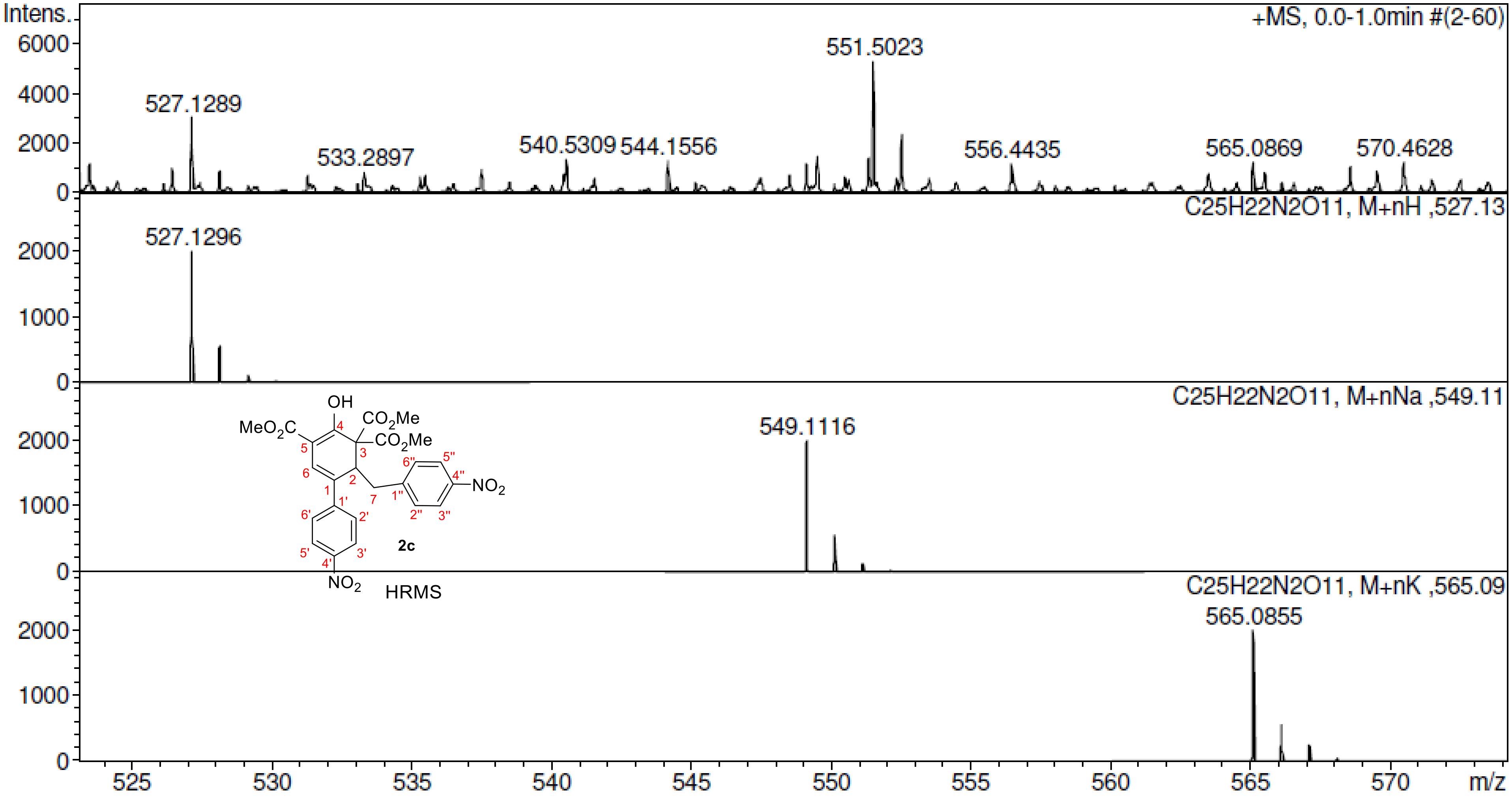
Intens.
+MS, 0.0-1.0min #(2-60)
551.5023
527.1289
533.2897
540.5309
544.1556
556.4435
565.0869
570.4628
C25H22N2O11, M+nH ,527.13
527.1296
C25H22N2O11, M+nNa ,549.11
549.1116
C25H22N2O11, M+nK ,565.09
565.0855
MeO2C
OH
CO2Me
CO2Me
NO2
NO2
2c
HRMS
m/z

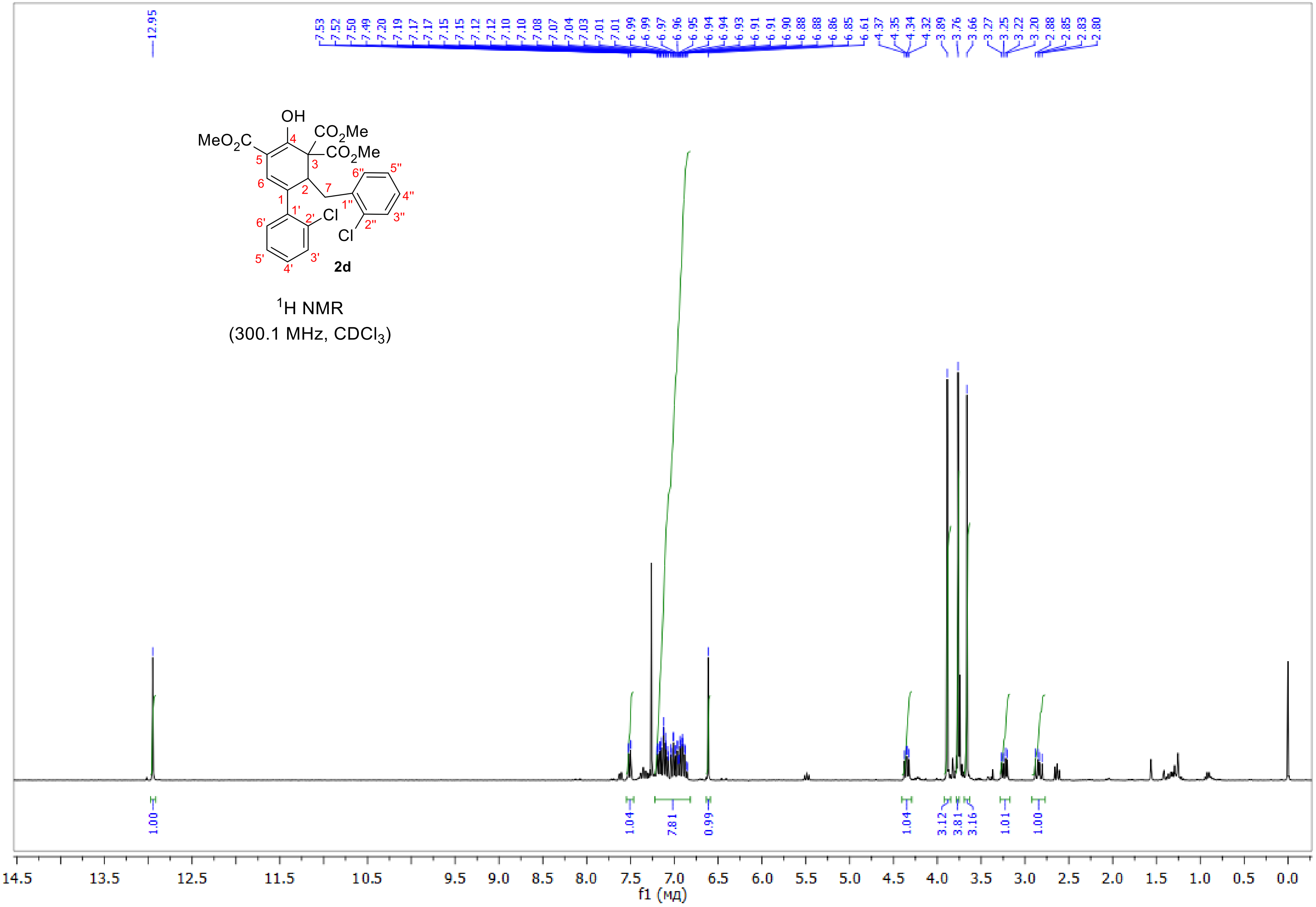

OH
MeO2C
CO2Me
CO2Me
Cl
Cl
2d
1H NMR
(300.1 MHz, CDCl3)
f1 (мд)

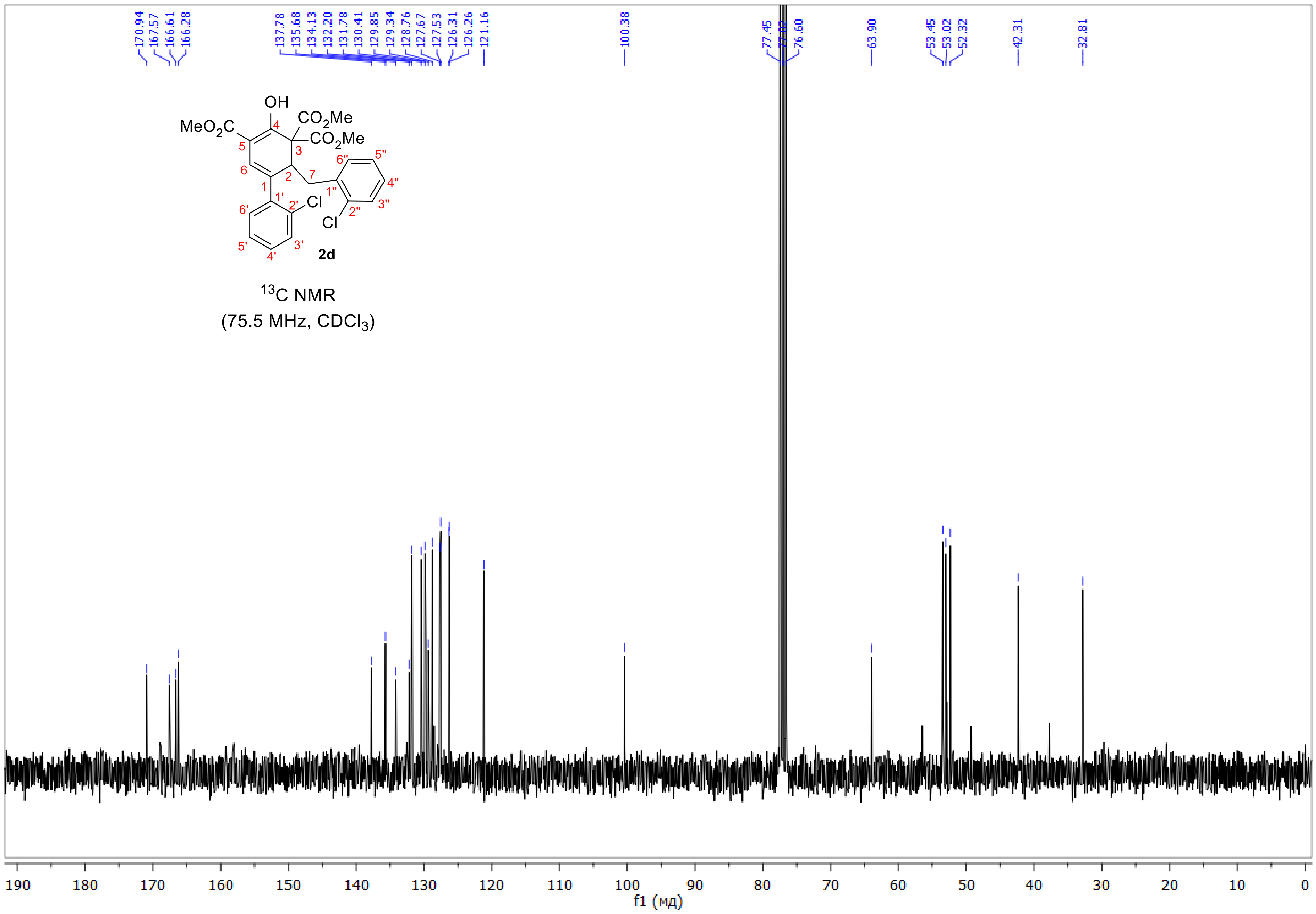
170.94
167.57
166.61
166.28
137.78
135.68
134.13
132.20
131.78
130.41
129.85
129.34
128.76
127.67
127.53
126.31
126.26
121.16
100.38
77.45
76.60
63.90
53.45
53.02
52.32
42.31
32.81
OH
MeO_2C
CO_2Me
CO_2Me
Cl
Cl
2d
^{13}C NMR
(75.5 MHz, $CDCl_3$)
f1 (мд)

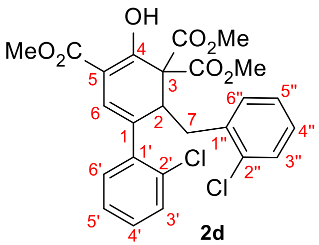

$^1H,^1H$-COSY
(300.1 MHz, $CDCl_3$)

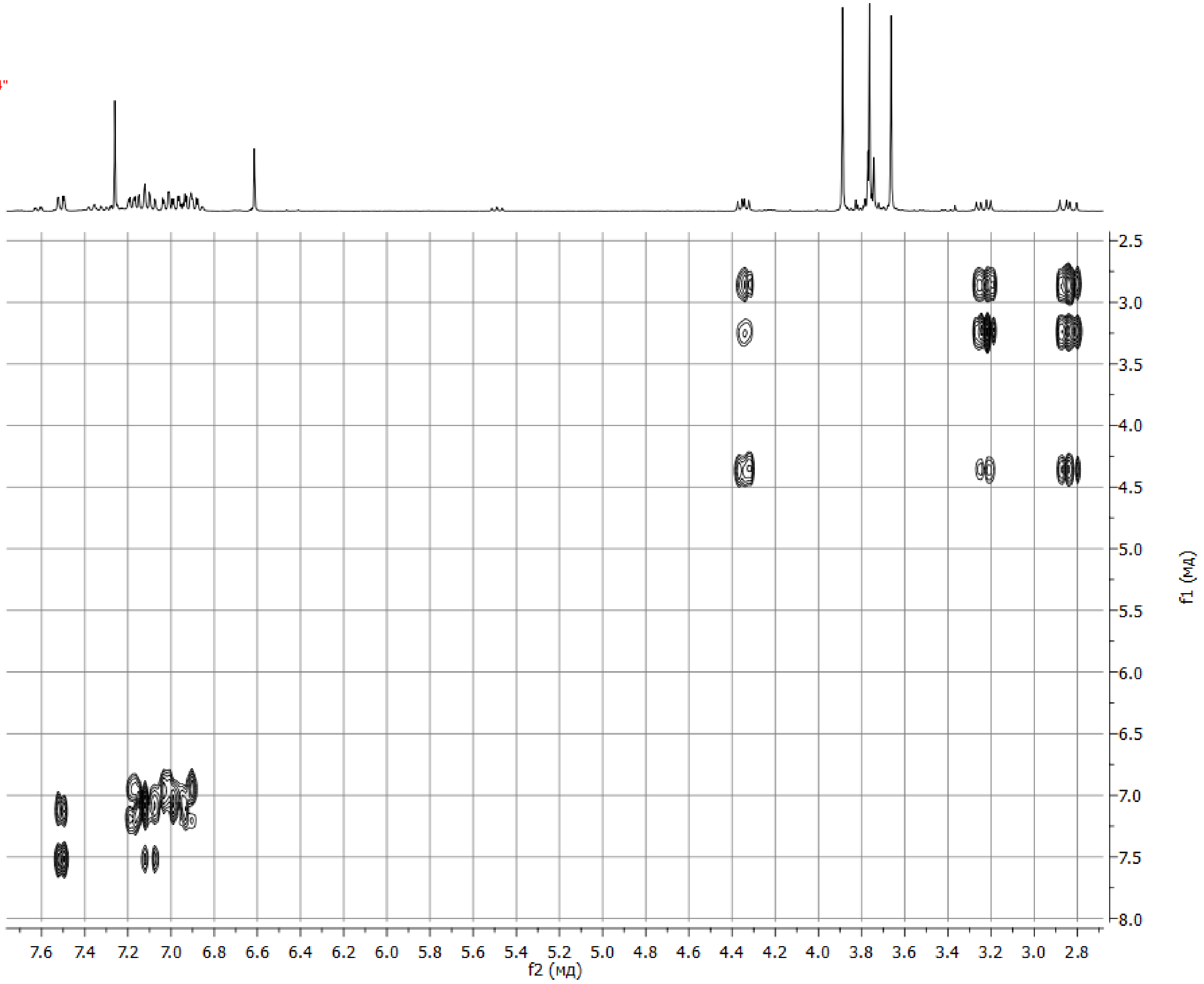

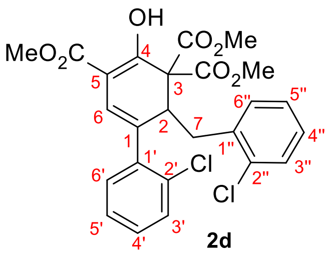

2d

$^{1}H,^{13}C$-HSQC
(^{1}H: 300.1 MHz,
^{13}C: 75.5 MHz
$CDCl_3$)

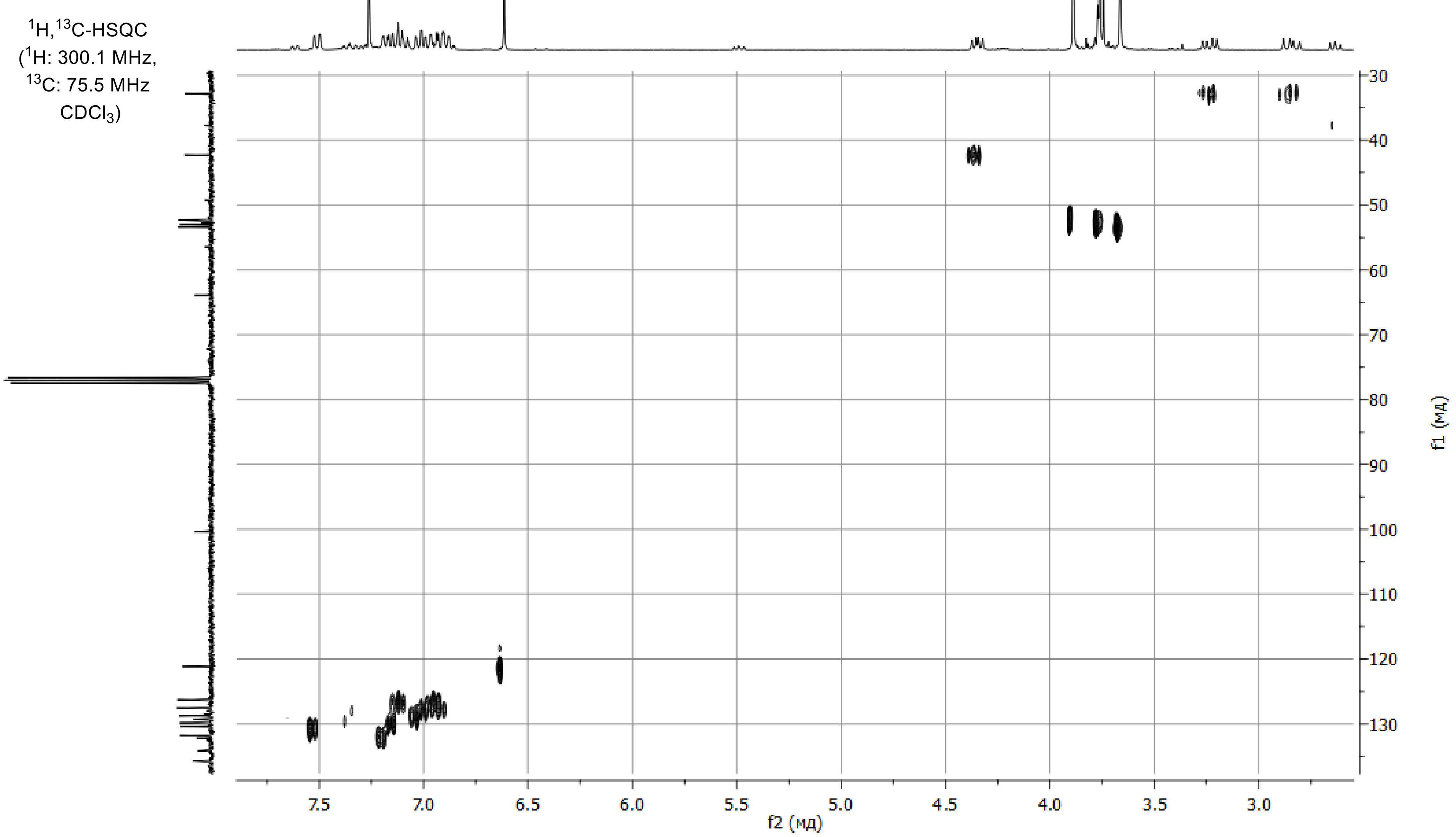

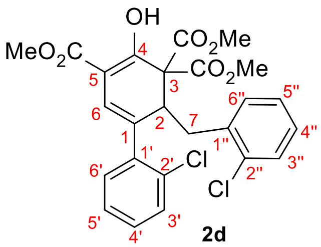

2d

^{1}H,^{13}C-HMBC
(^{1}H: 300.1 MHz,
^{13}C: 75.5 MHz
$CDCl_3$)

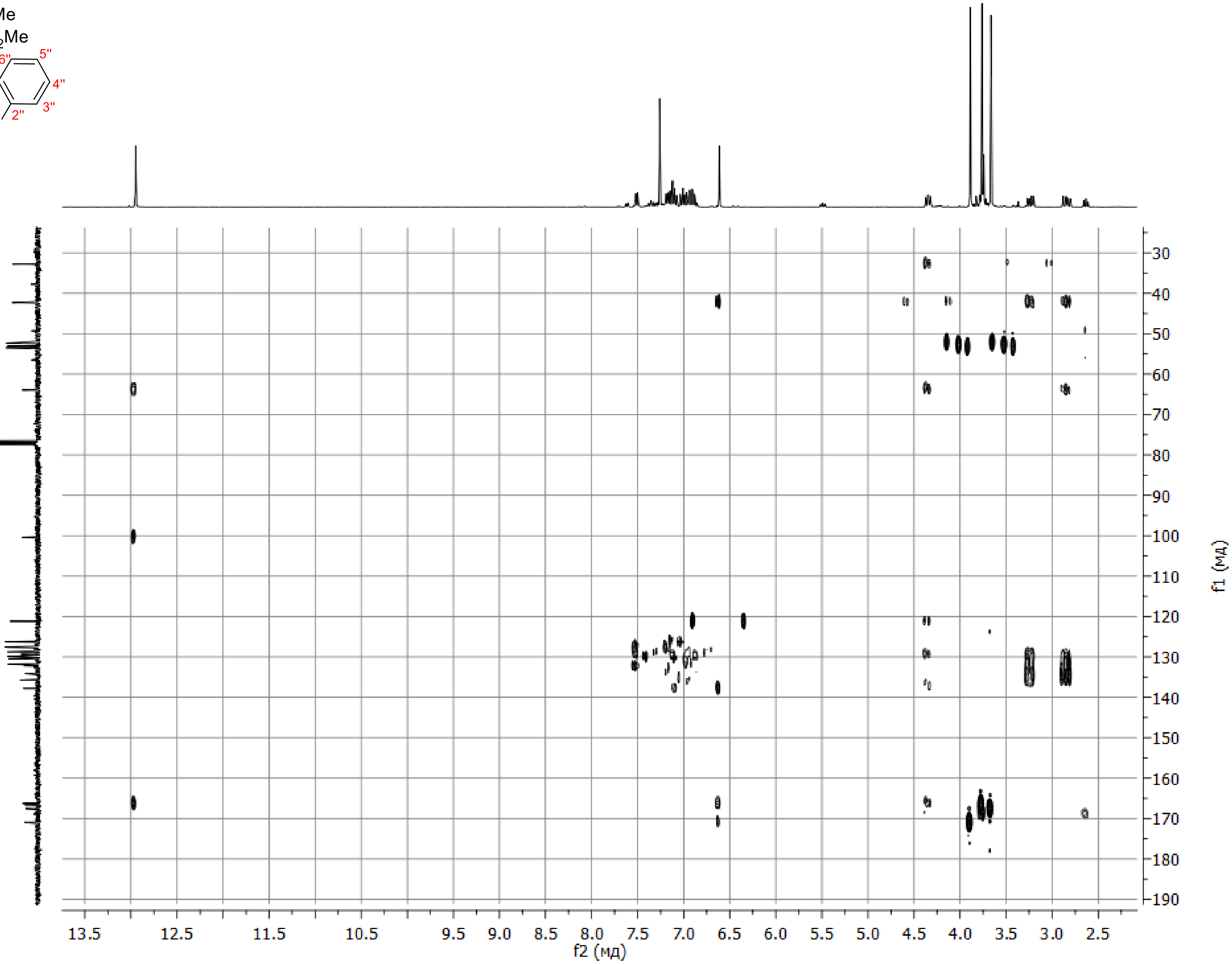

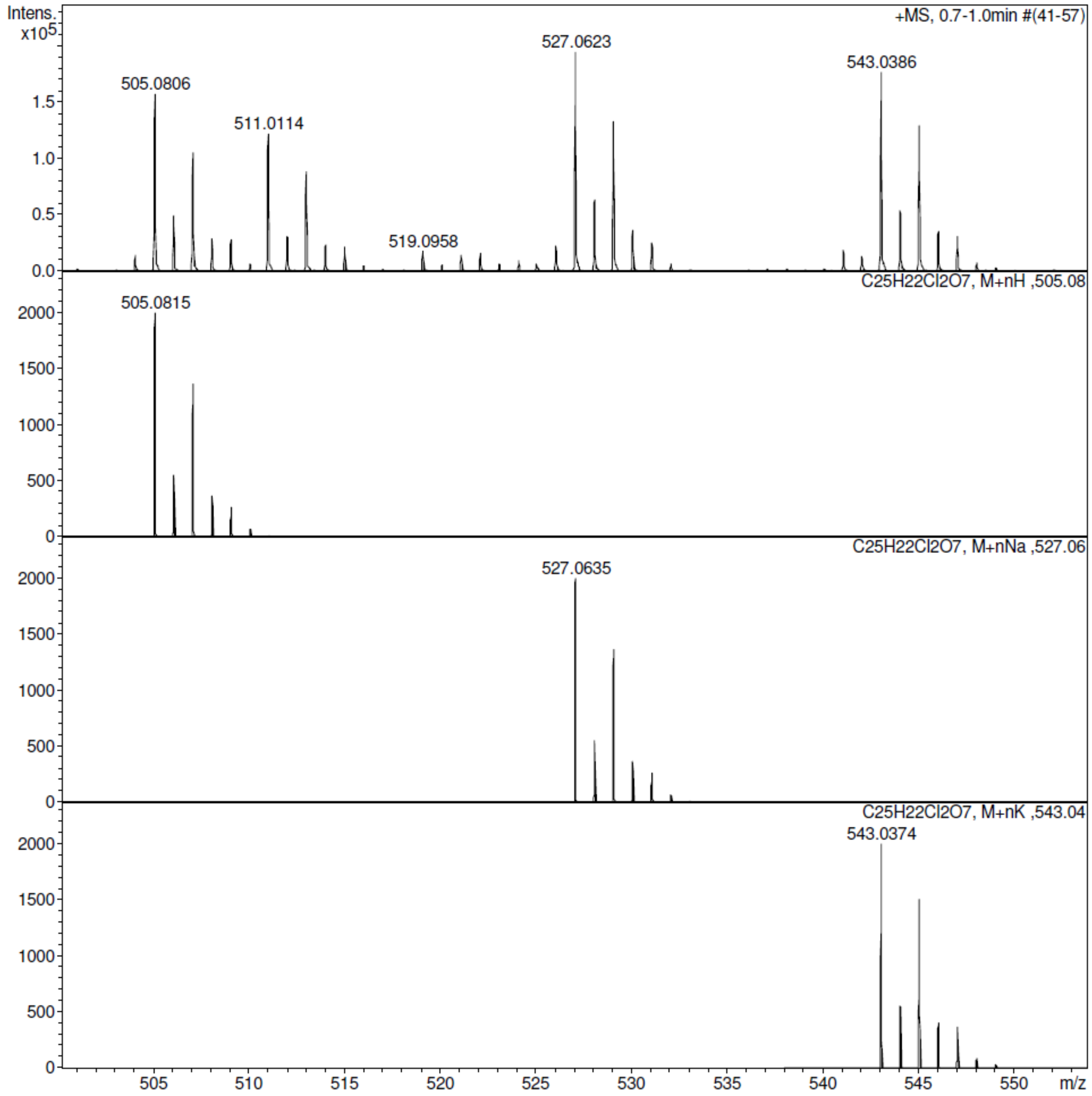

Intens.
x10^5
+MS, 0.7-1.0min #(41-57)
505.0806
511.0114
519.0958
527.0623
543.0386
C25H22Cl2O7, M+nH ,505.08
505.0815
C25H22Cl2O7, M+nNa ,527.06
527.0635
C25H22Cl2O7, M+nK ,543.04
543.0374
m/z

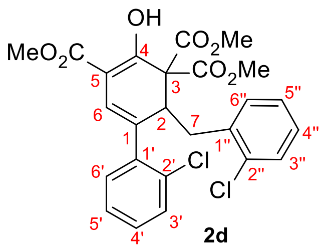

2d

HRMS

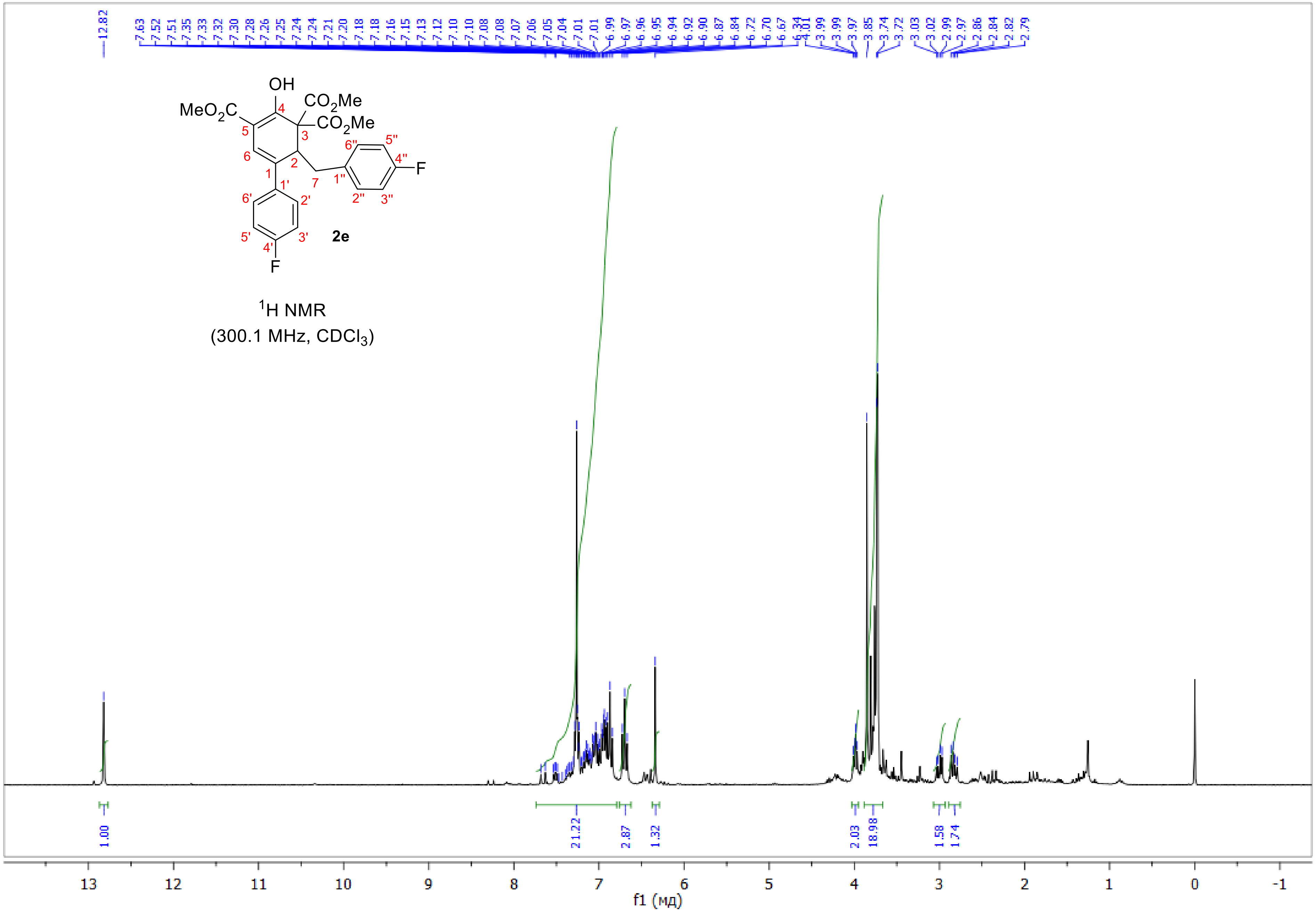

2e
^{1}H NMR
(300.1 MHz, $CDCl_3$)
f1 (мд)

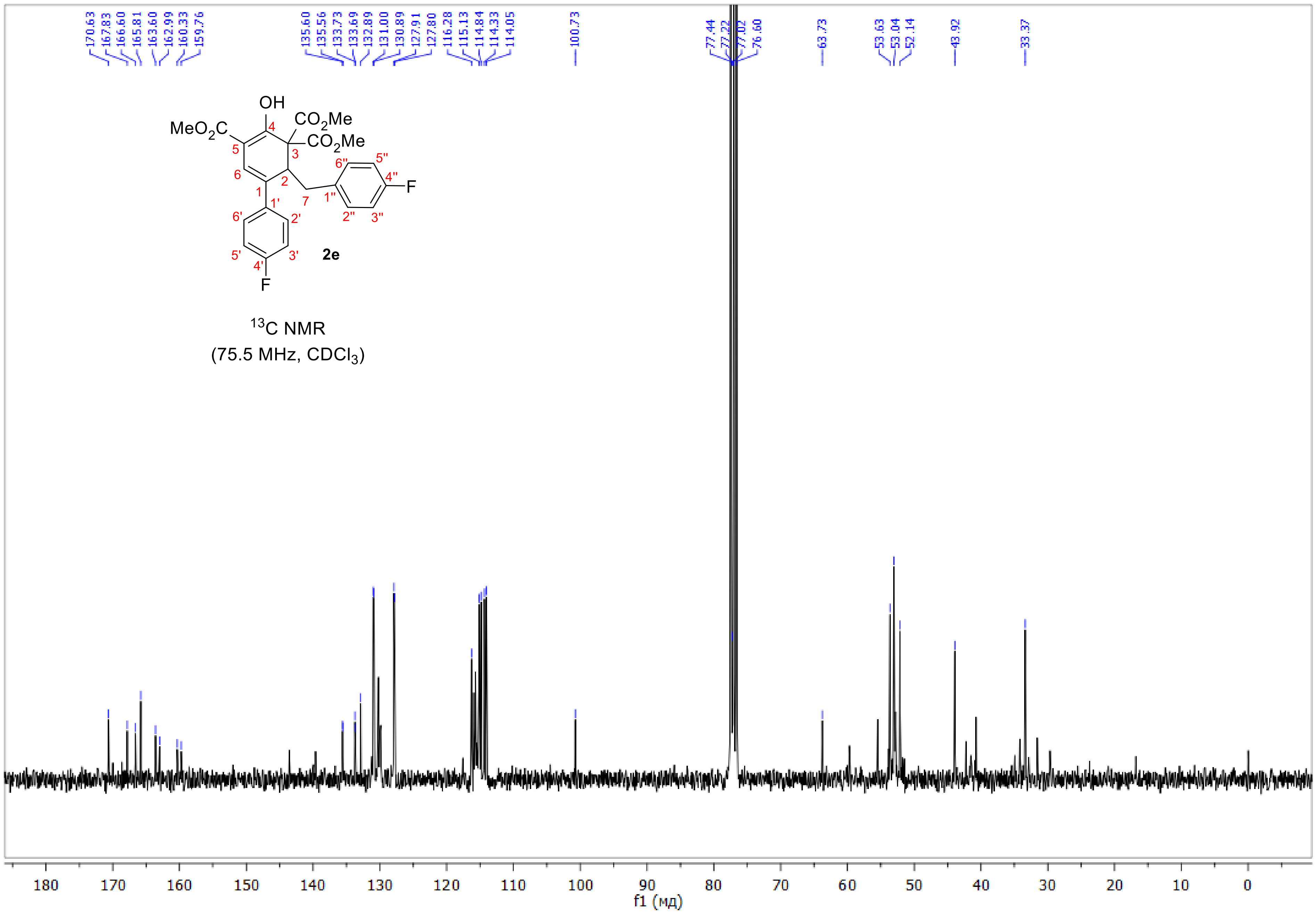

170.63
167.83
166.60
165.81
163.60
162.99
160.33
159.76
135.60
135.56
133.73
133.69
132.89
131.00
130.89
127.91
127.80
116.28
115.13
114.84
114.33
114.05
100.73
77.44
77.22
77.02
76.60
63.73
53.63
53.04
52.14
43.92
33.37
OH
MeO_2C
CO_2Me
CO_2Me
F
F
2e
^{13}C NMR
(75.5 MHz, $CDCl_3$)
f1 (мд)

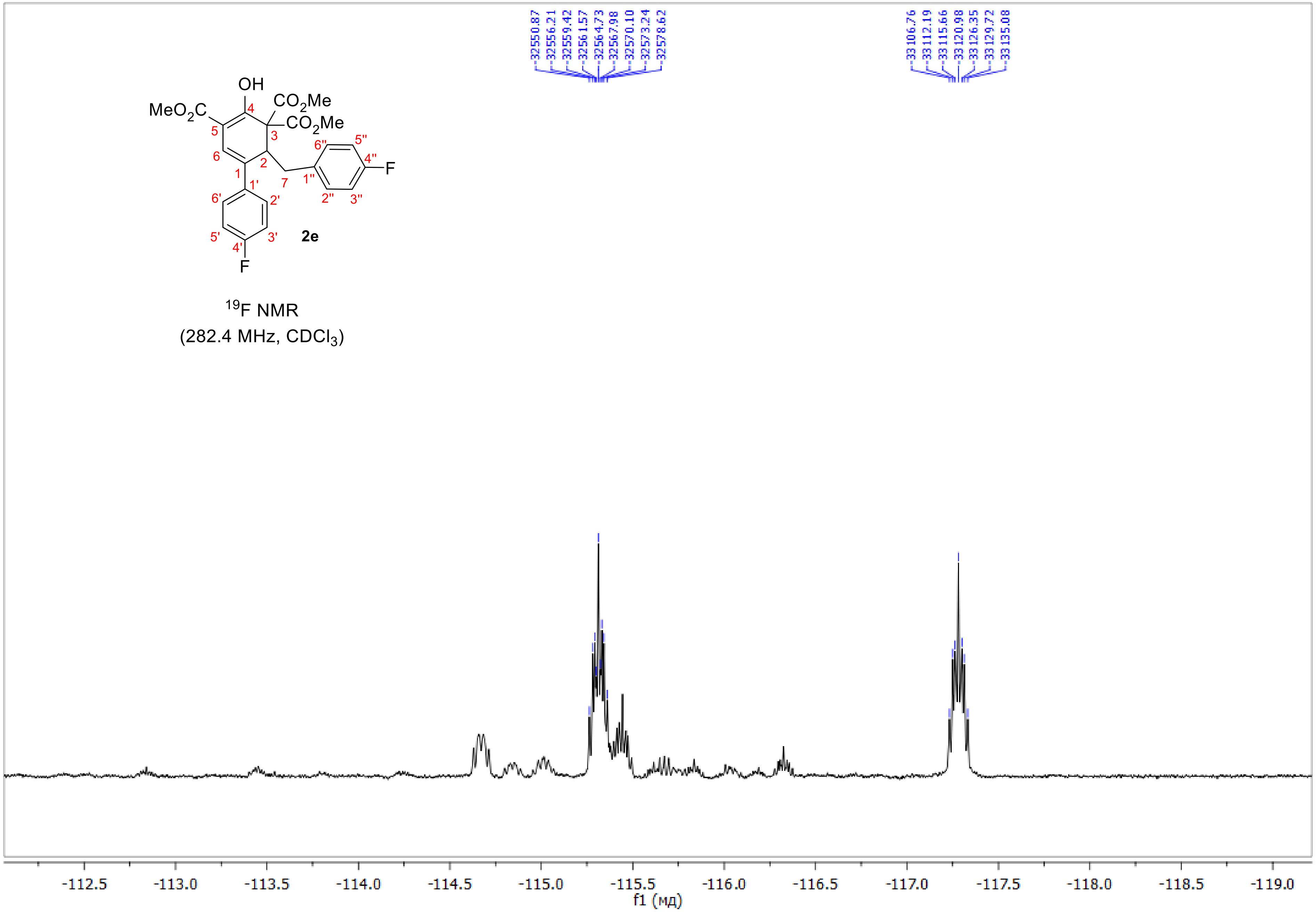
OH
MeO2C
CO2Me
CO2Me
F
F
2e
19F NMR
(282.4 MHz, CDCl3)
-32550.87
-32556.21
-32559.42
-32561.57
-32564.73
-32567.98
-32570.10
-32573.24
-32578.62
-33106.76
-33112.19
-33115.66
-33120.98
-33126.35
-33129.72
-33135.08
-112.5
-113.0
-113.5
-114.0
-114.5
-115.0
-115.5
-116.0
-116.5
-117.0
-117.5
-118.0
-118.5
-119.0
f1 (мд)

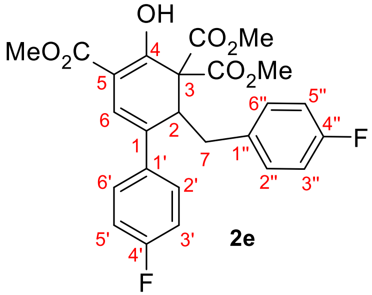

2e

$^{1}H,^{1}H$-COSY
(300.1 MHz, $CDCl_3$)

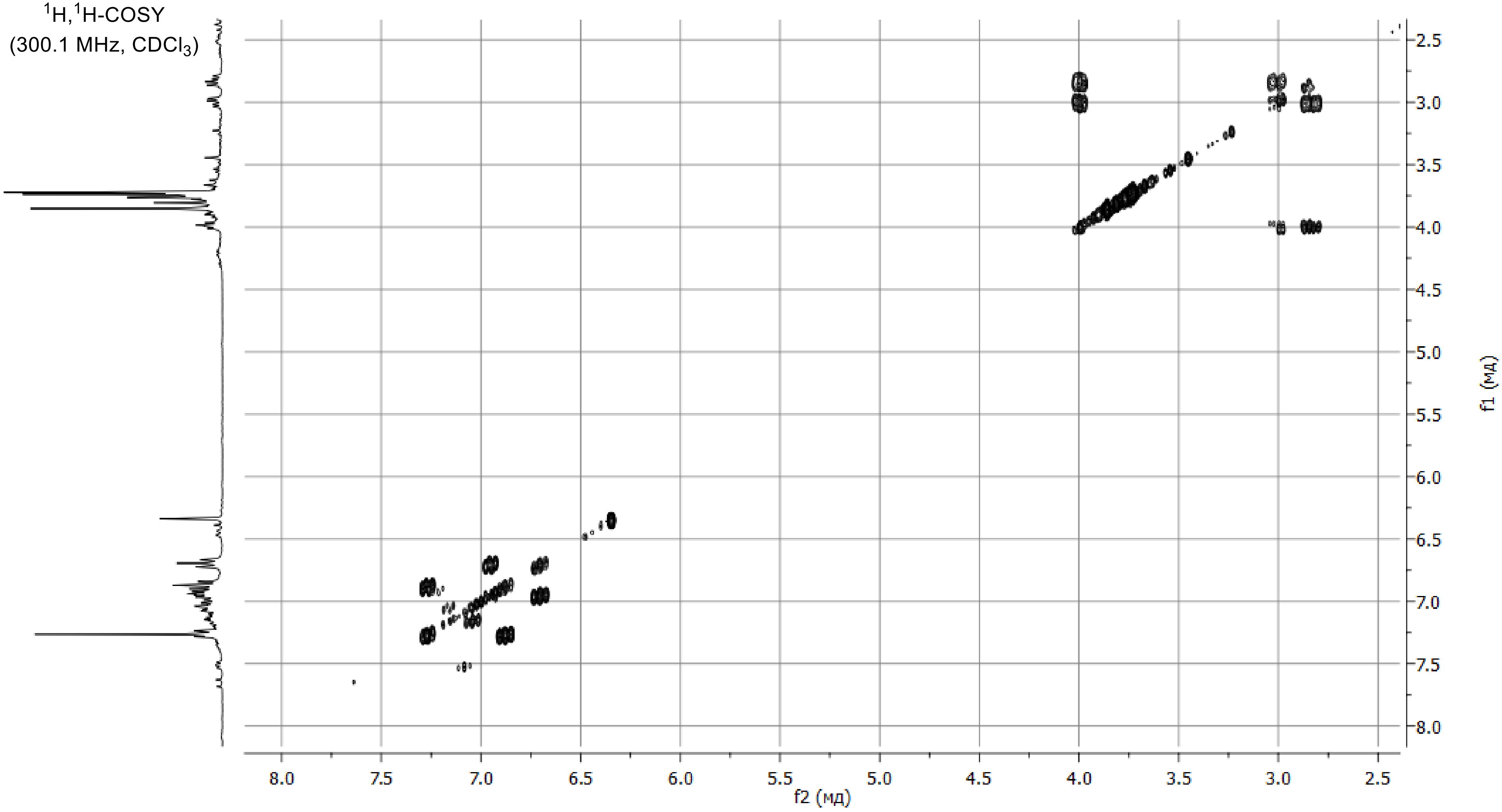

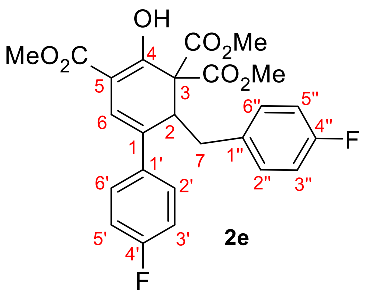

^{1}H,^{13}C-HSQC
(^{1}H: 300.1 MHz,
^{13}C: 75.5 MHz
$CDCl_3$)

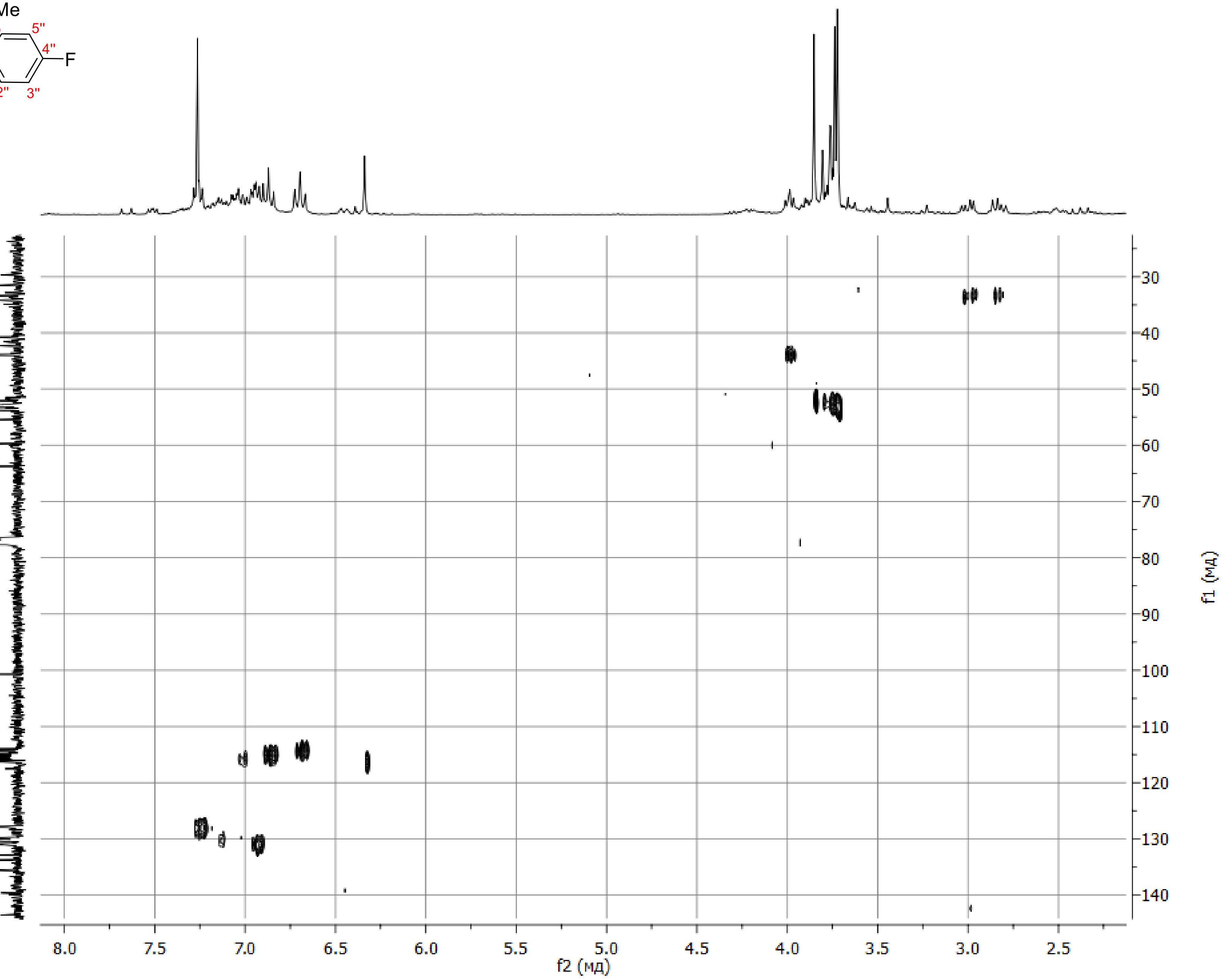

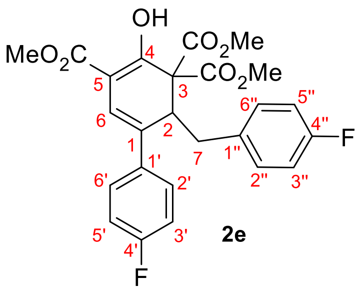

2e

^1H,^{13}C-HMBC
(^1H: 300.1 MHz,
^{13}C: 75.5 MHz
$CDCl_3$)

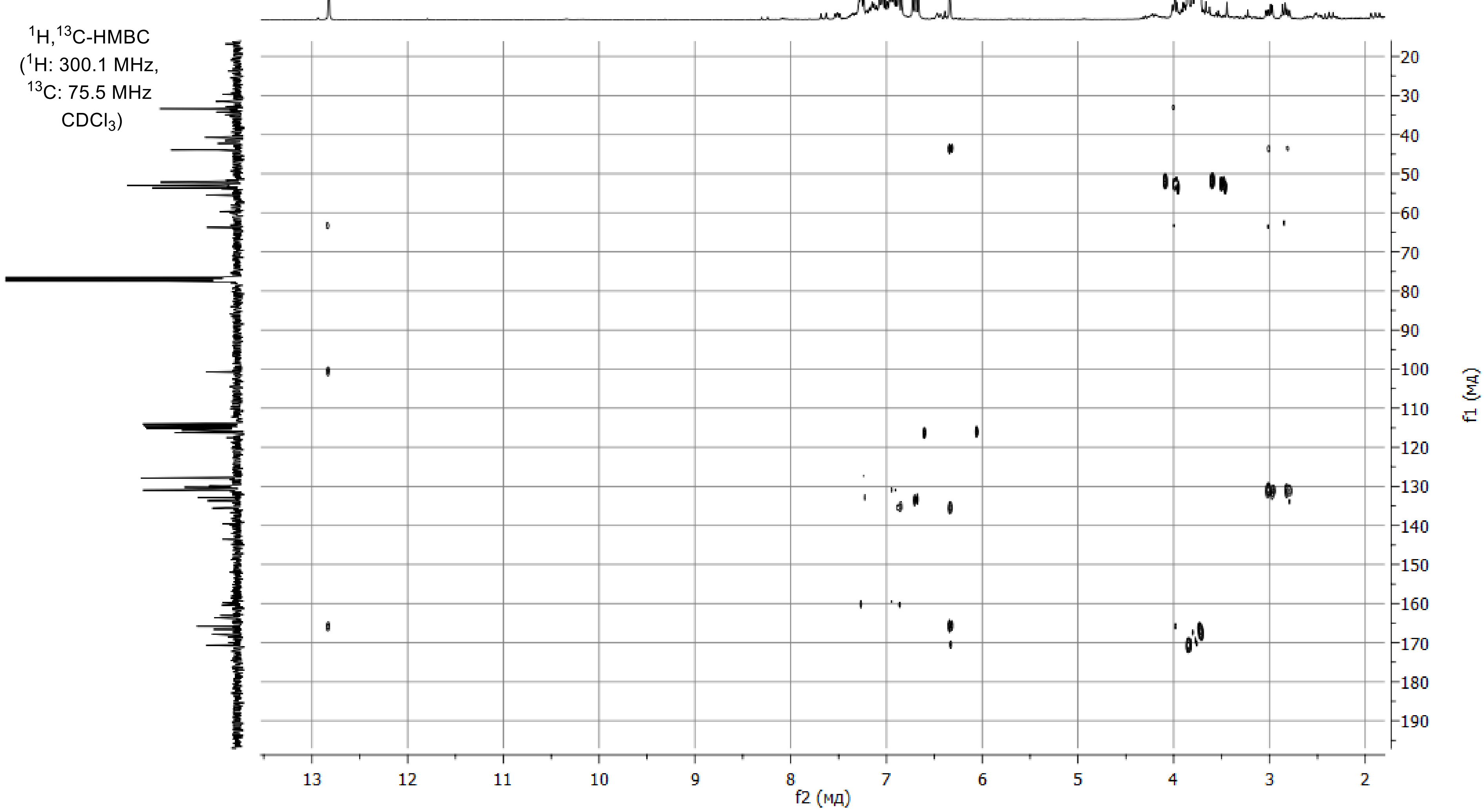

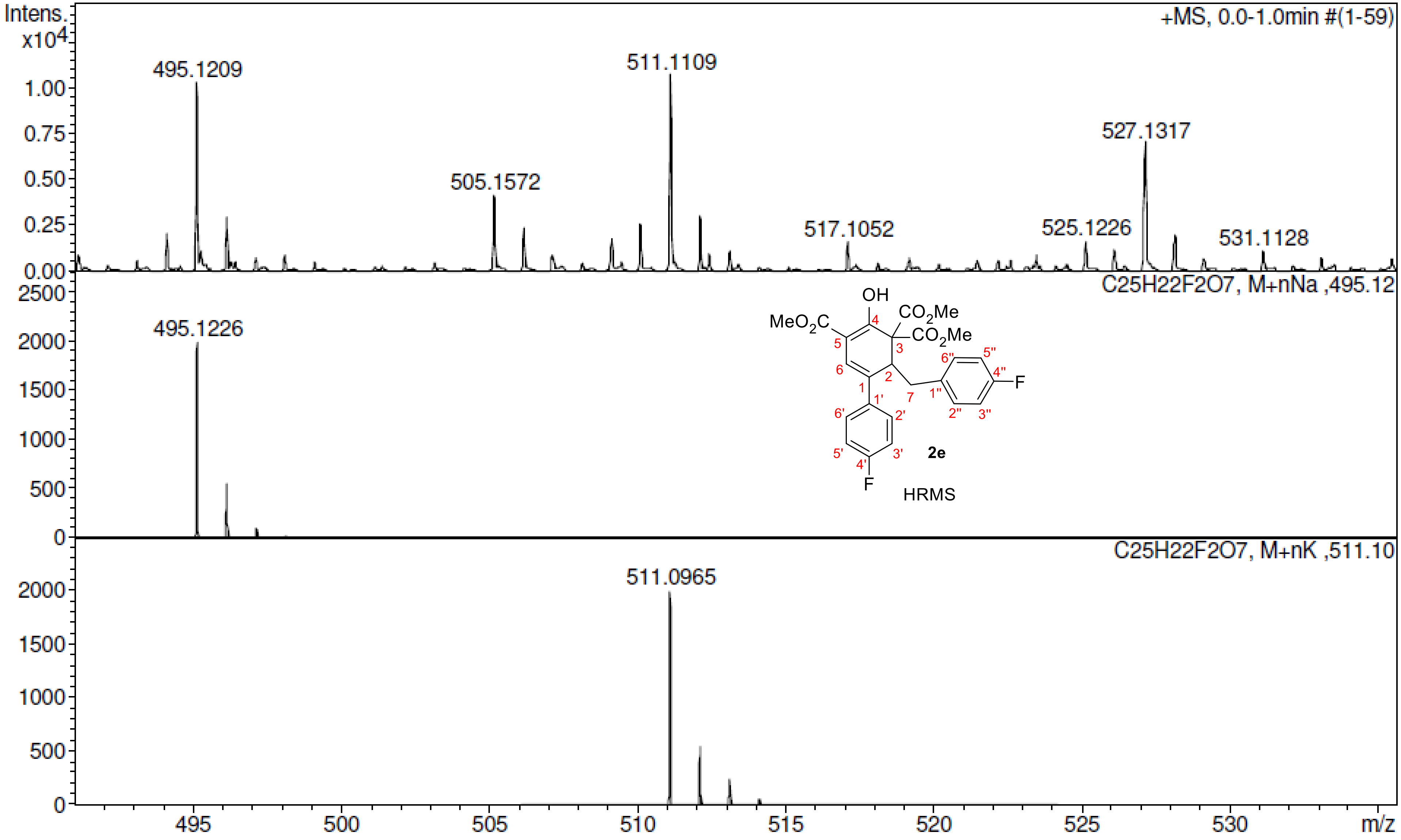
Intens.
x10^4
+MS, 0.0-1.0min #(1-59)
495.1209
511.1109
505.1572
527.1317
517.1052
525.1226
531.1128
C25H22F2O7, M+nNa ,495.12
495.1226
MeO2C
OH
CO2Me
CO2Me
F
F
2e
HRMS
C25H22F2O7, M+nK ,511.10
511.0965
m/z

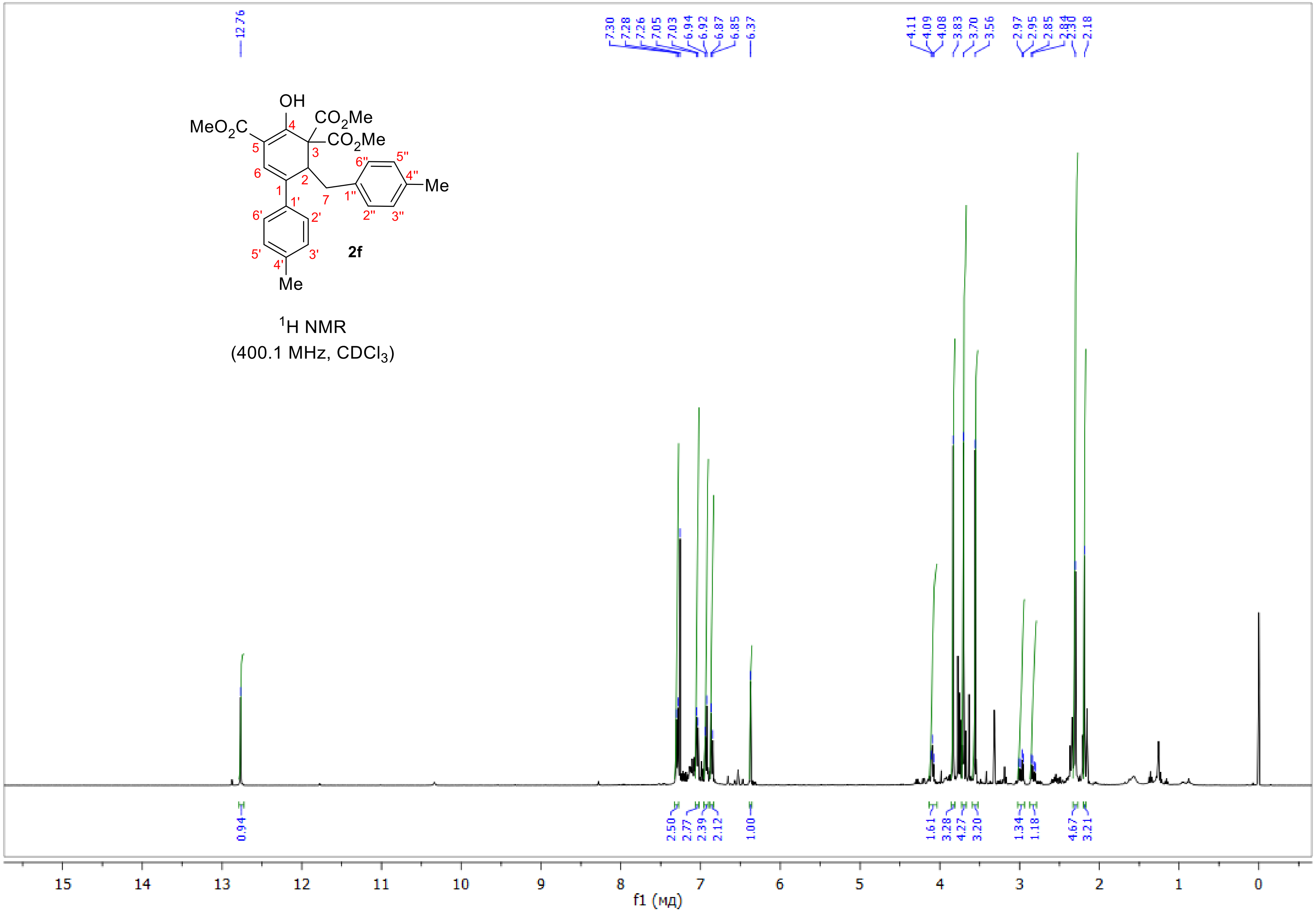

12.76
7.30
7.28
7.26
7.05
7.03
6.94
6.92
6.87
6.85
6.37
4.11
4.09
4.08
3.83
3.70
3.56
2.97
2.95
2.85
2.84
2.30
2.18
OH
MeO2C
CO2Me
CO2Me
Me
Me
2f
1H NMR
(400.1 MHz, CDCl3)
0.94
2.50
2.77
2.39
2.12
1.00
1.61
3.28
4.27
3.20
1.34
1.18
4.67
3.21
f1 (мд)

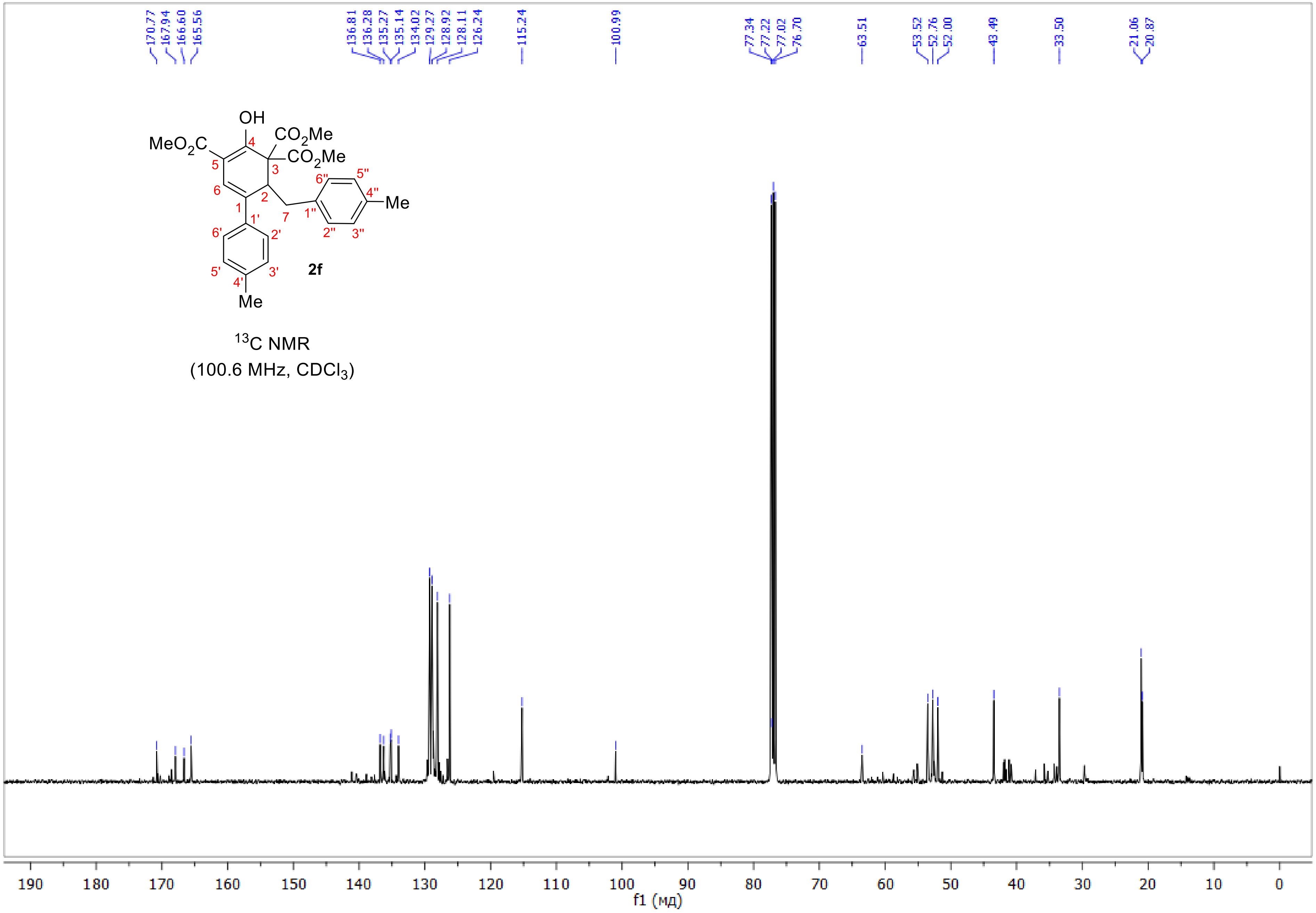

170.77
167.94
166.60
165.56
136.81
136.28
135.27
135.14
134.02
129.27
128.92
128.11
126.24
115.24
100.99
77.34
77.22
77.02
76.70
63.51
53.52
52.76
52.00
43.49
33.50
21.06
20.87
OH
MeO2C
CO2Me
CO2Me
Me
Me
2f
13C NMR
(100.6 MHz, CDCl3)
f1 (мд)

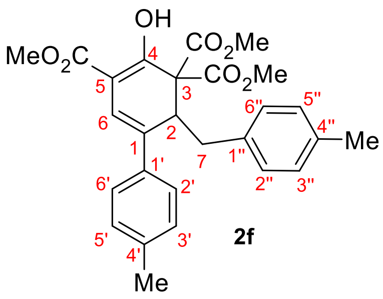

2f

^{1}H,^{1}H-COSY
(400.1 MHz, $CDCl_3$)

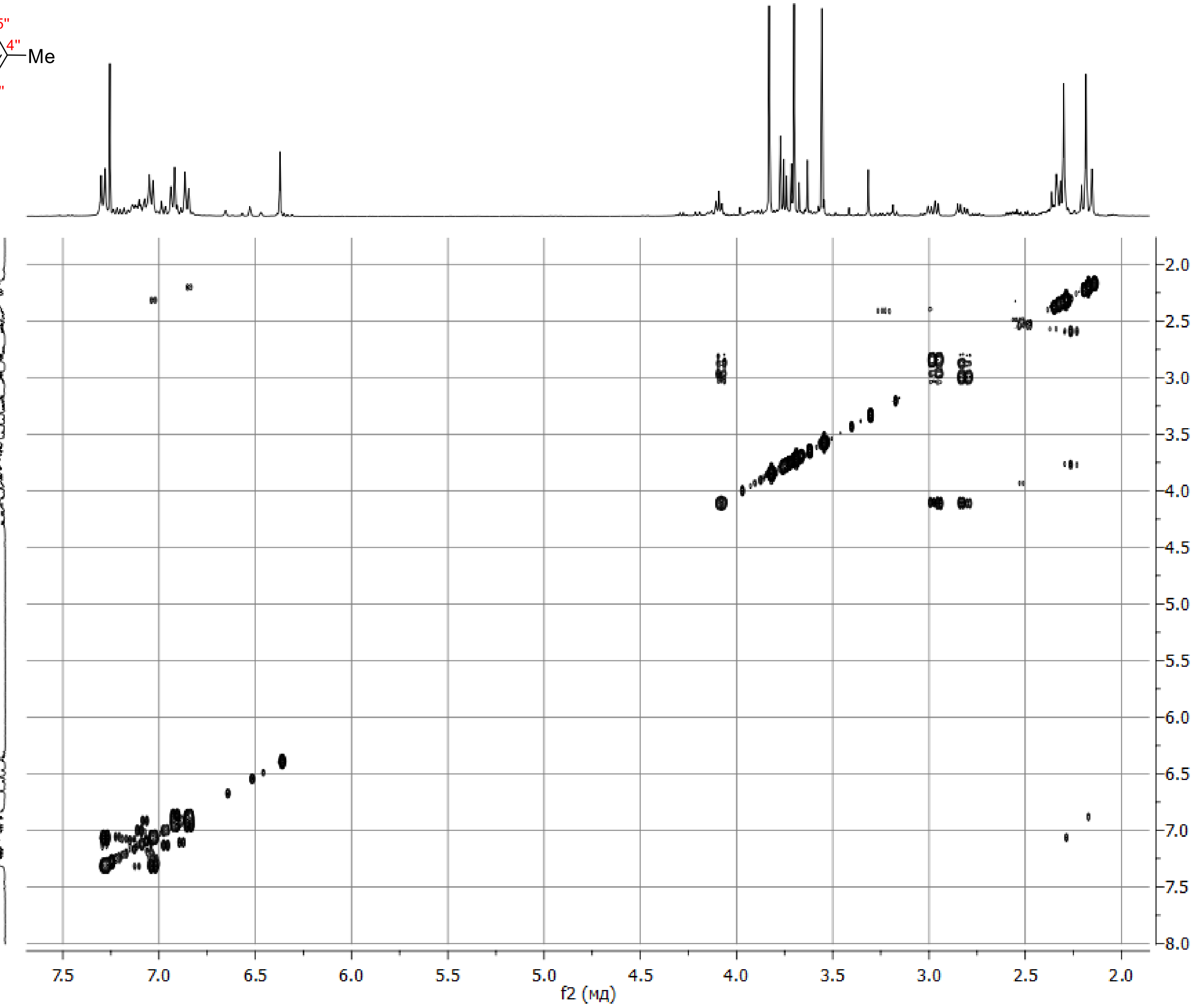

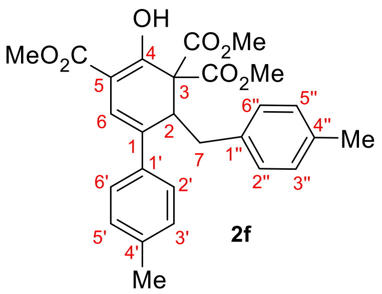

^{1}H,^{13}C-HSQC
(^{1}H: 400.1 MHz,
^{13}C: 100.6 MHz
CDCl$_3$)

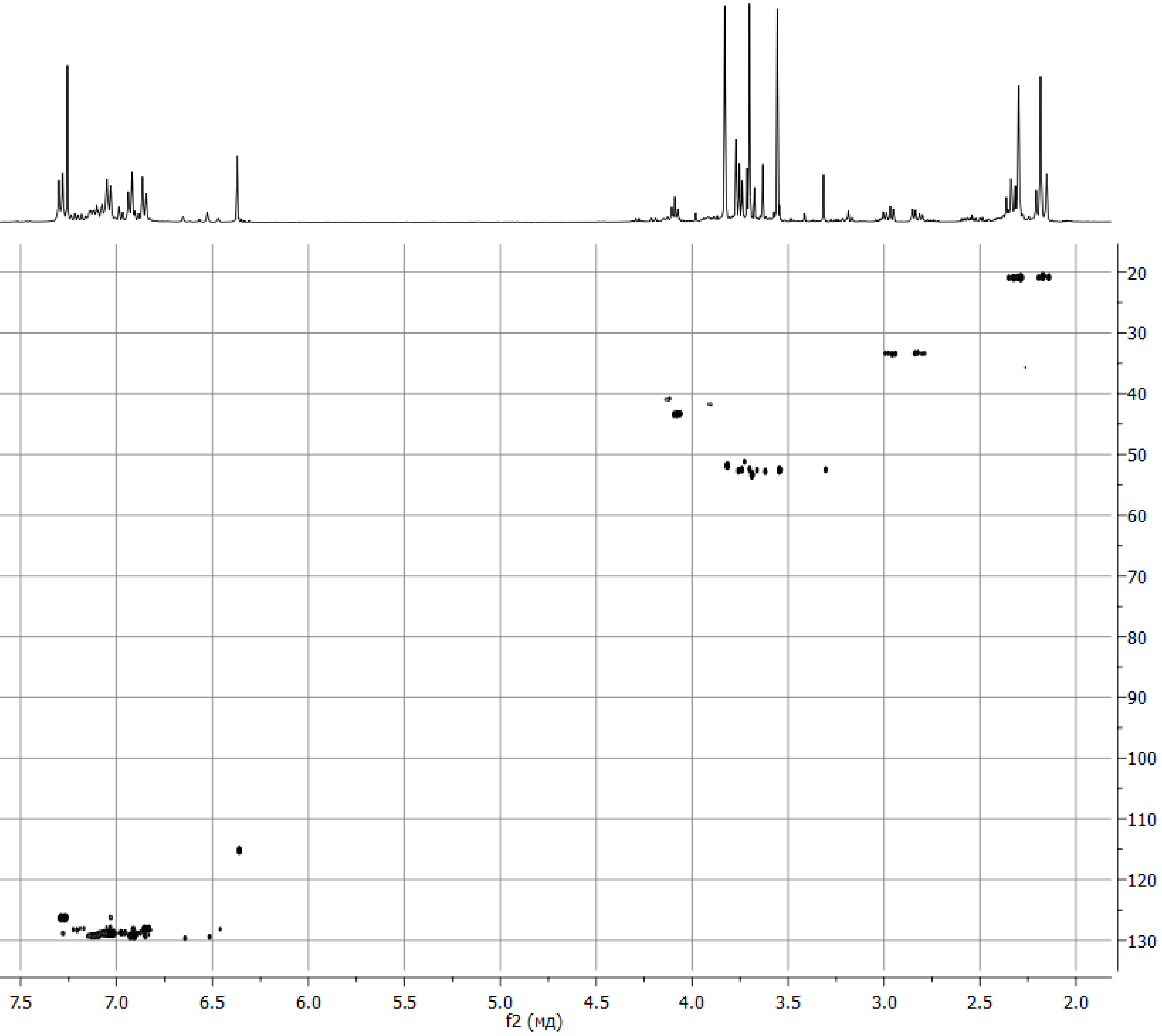

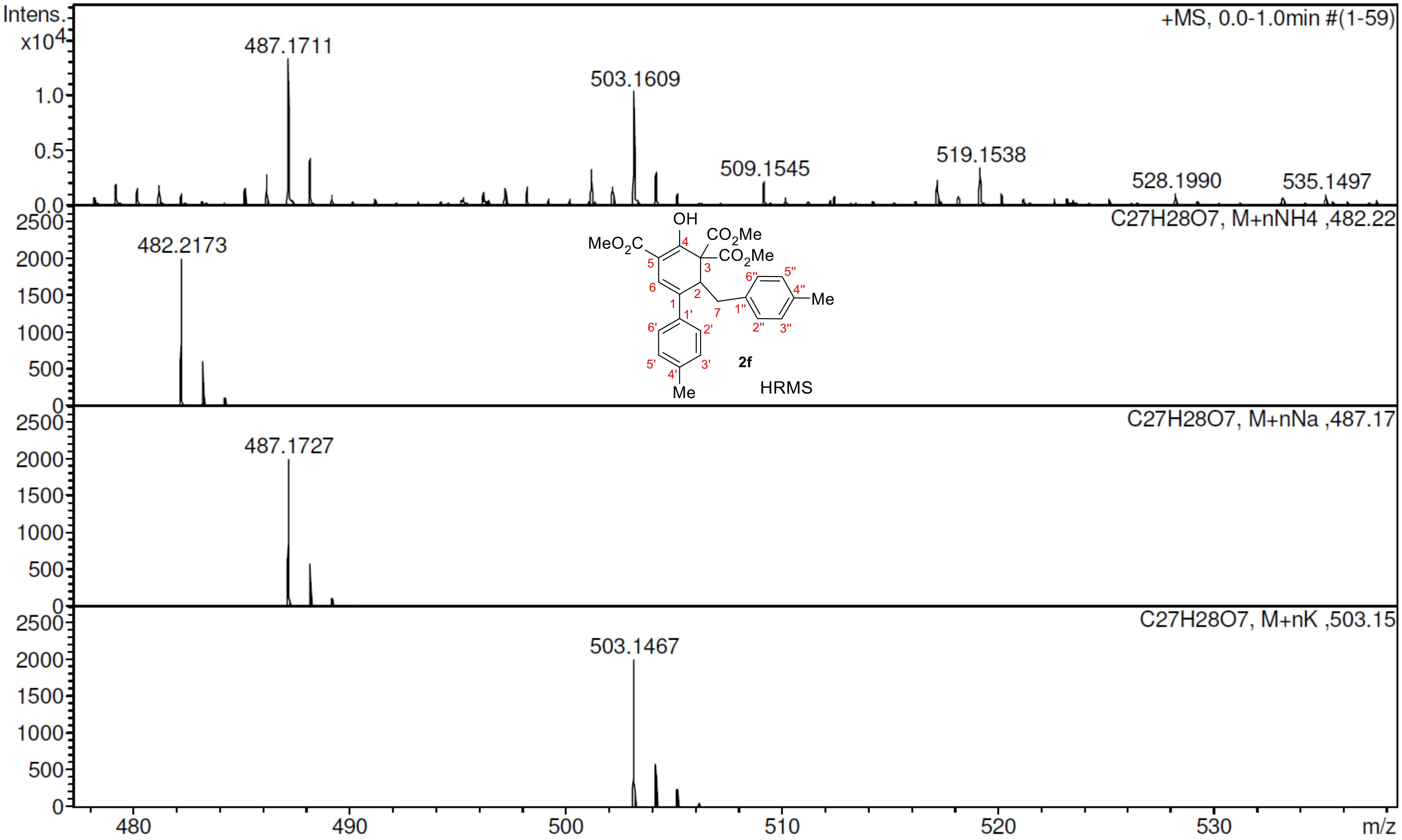
Intens.
x10^4
+MS, 0.0-1.0min #(1-59)
487.1711
503.1609
509.1545
519.1538
528.1990
535.1497
C27H28O7, M+nNH4 ,482.22
482.2173
C27H28O7, M+nNa ,487.17
487.1727
C27H28O7, M+nK ,503.15
503.1467
OH
MeO2C
CO2Me
CO2Me
Me
Me
2f
HRMS
m/z

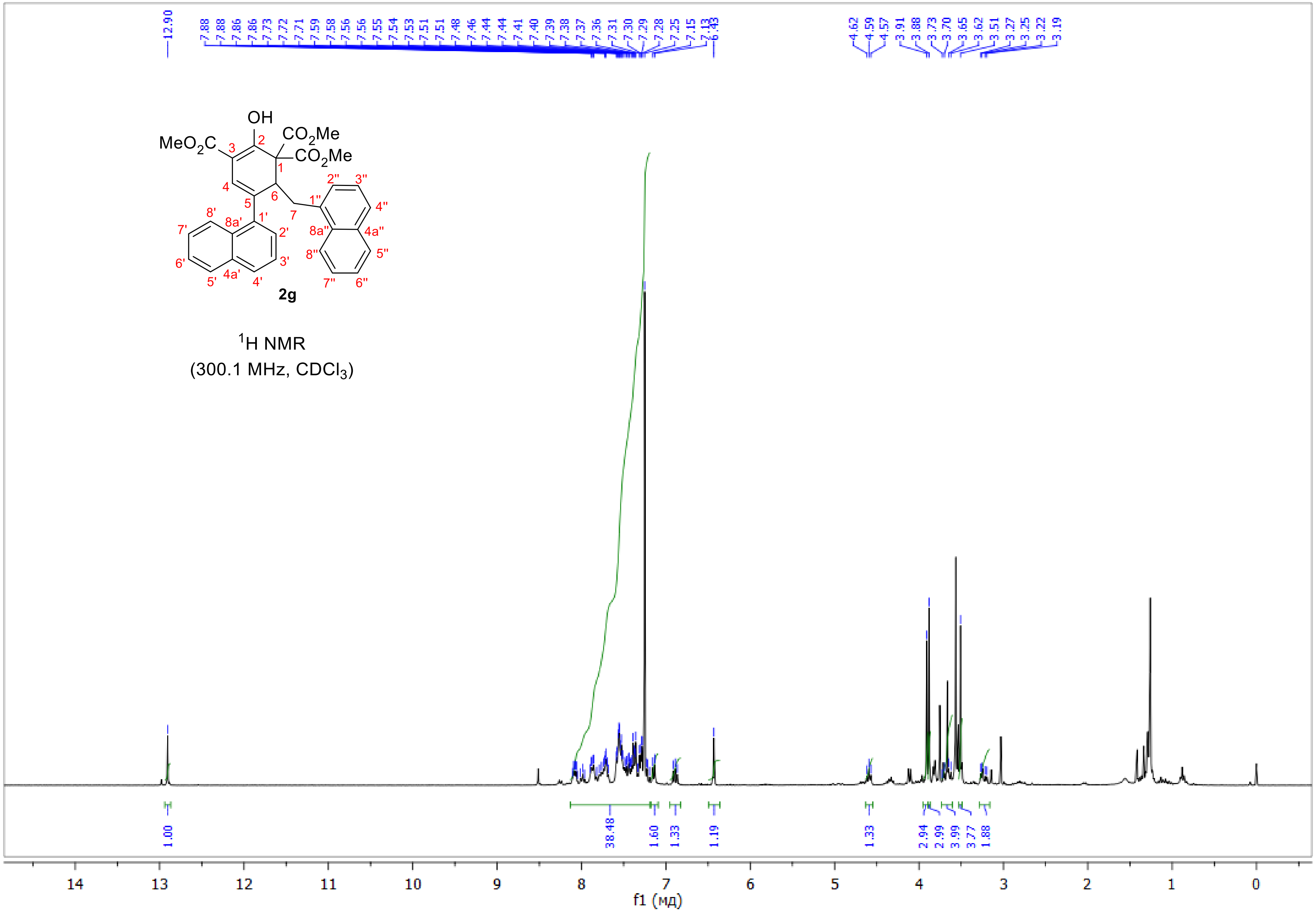

2g
^{1}H NMR
(300.1 MHz, $CDCl_3$)
f1 (мд)

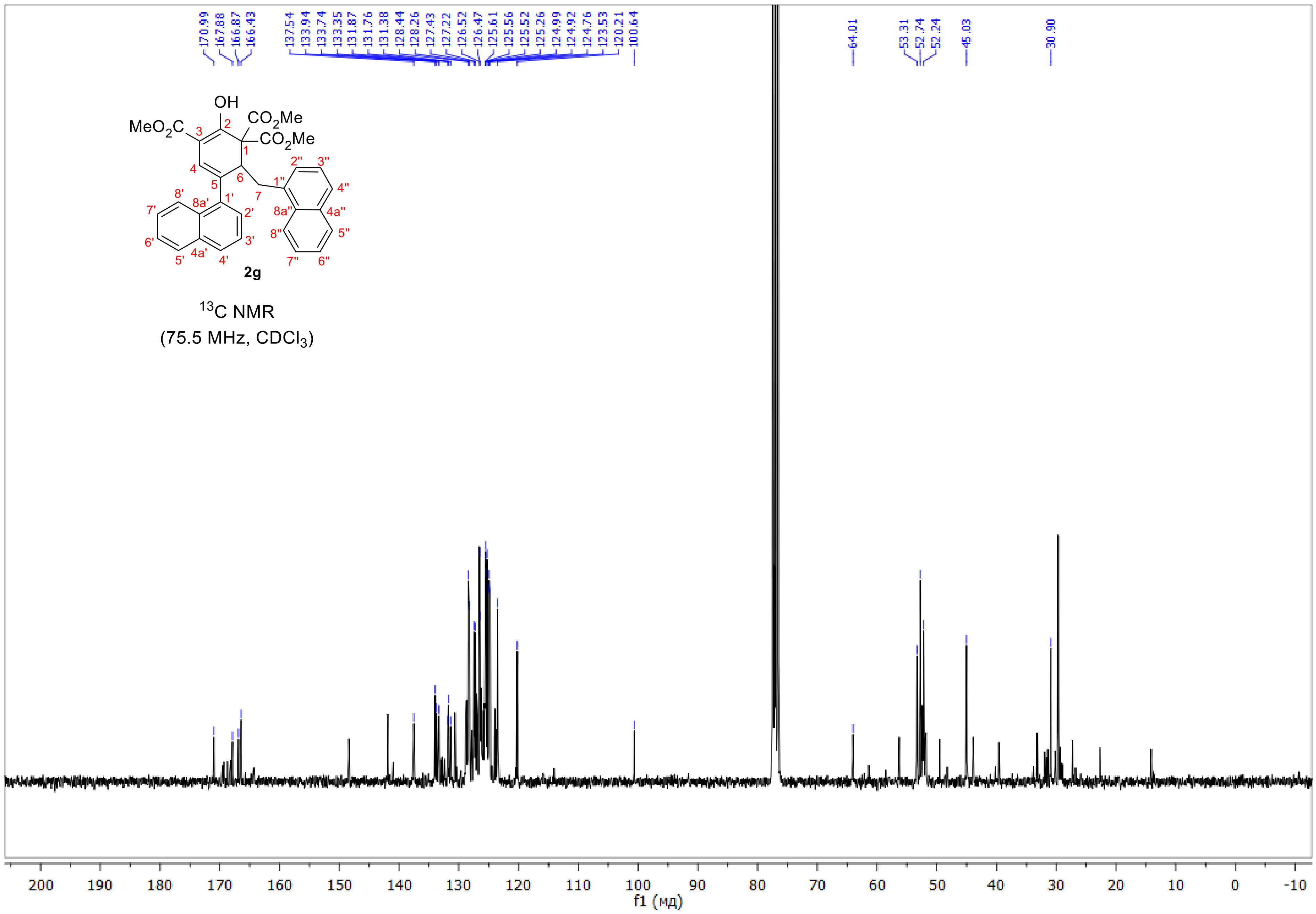
170.99
167.88
166.87
166.43
137.54
133.94
133.74
133.35
131.87
131.76
131.38
128.44
128.26
127.43
127.22
126.52
126.47
125.61
125.56
125.52
125.26
124.99
124.92
124.76
123.53
120.21
100.64
64.01
53.31
52.74
52.24
45.03
30.90
OH
MeO2C
CO2Me
CO2Me
2g
13C NMR
(75.5 MHz, CDCl3)
f1 (мд)

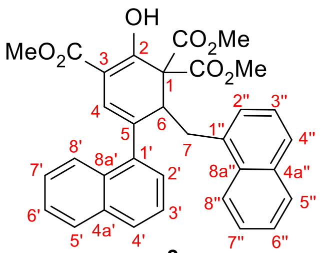

$^{1}H,^{1}H$-COSY

(300.1 MHz, $CDCl_3$)

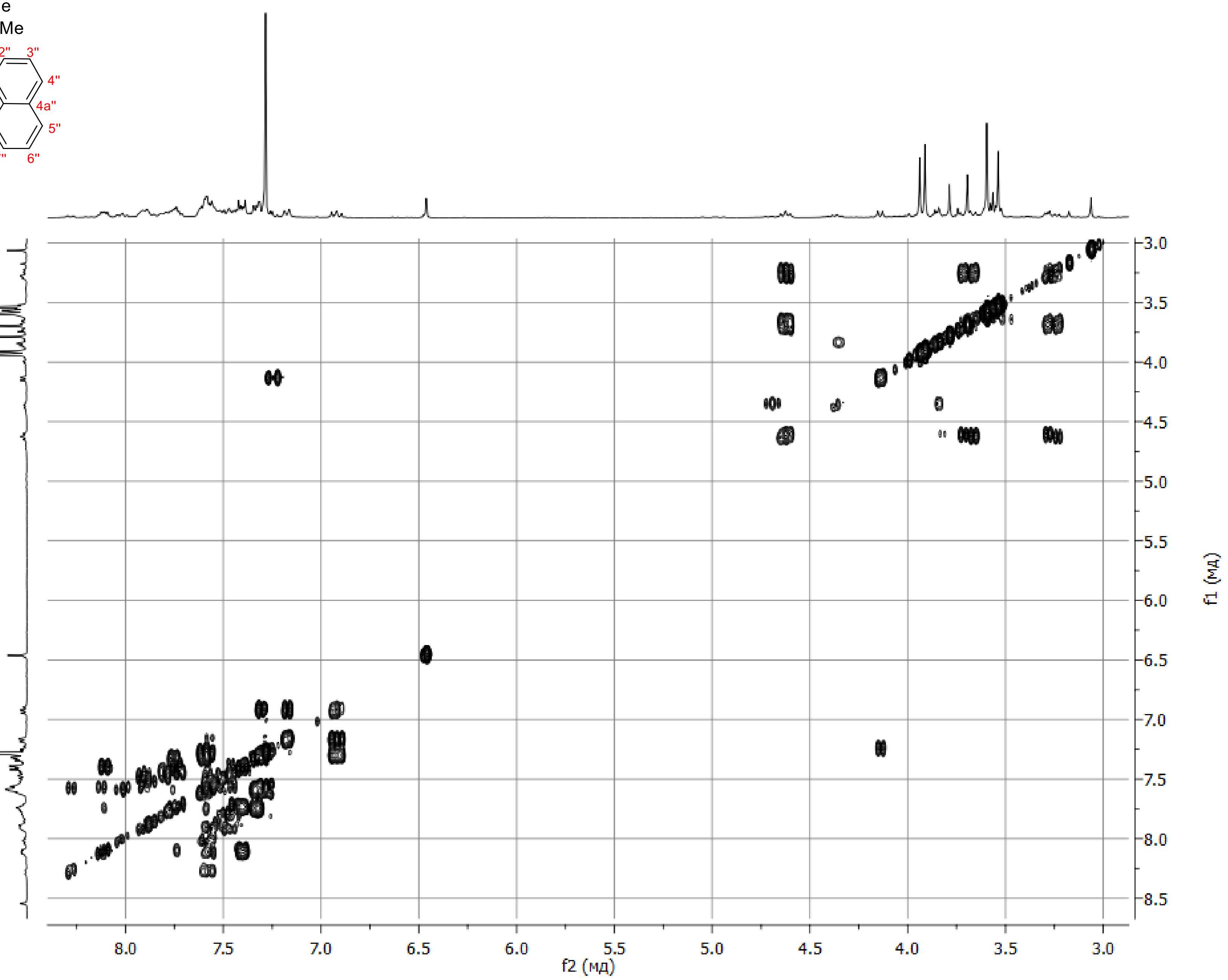

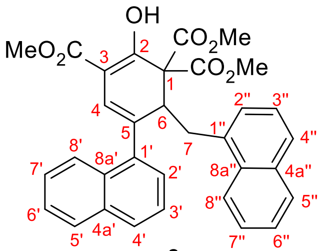

2g

^{1}H,^{13}C-HSQC
(^{1}H: 300.1 MHz,
^{13}C: 75.5 MHz
$CDCl_3$)

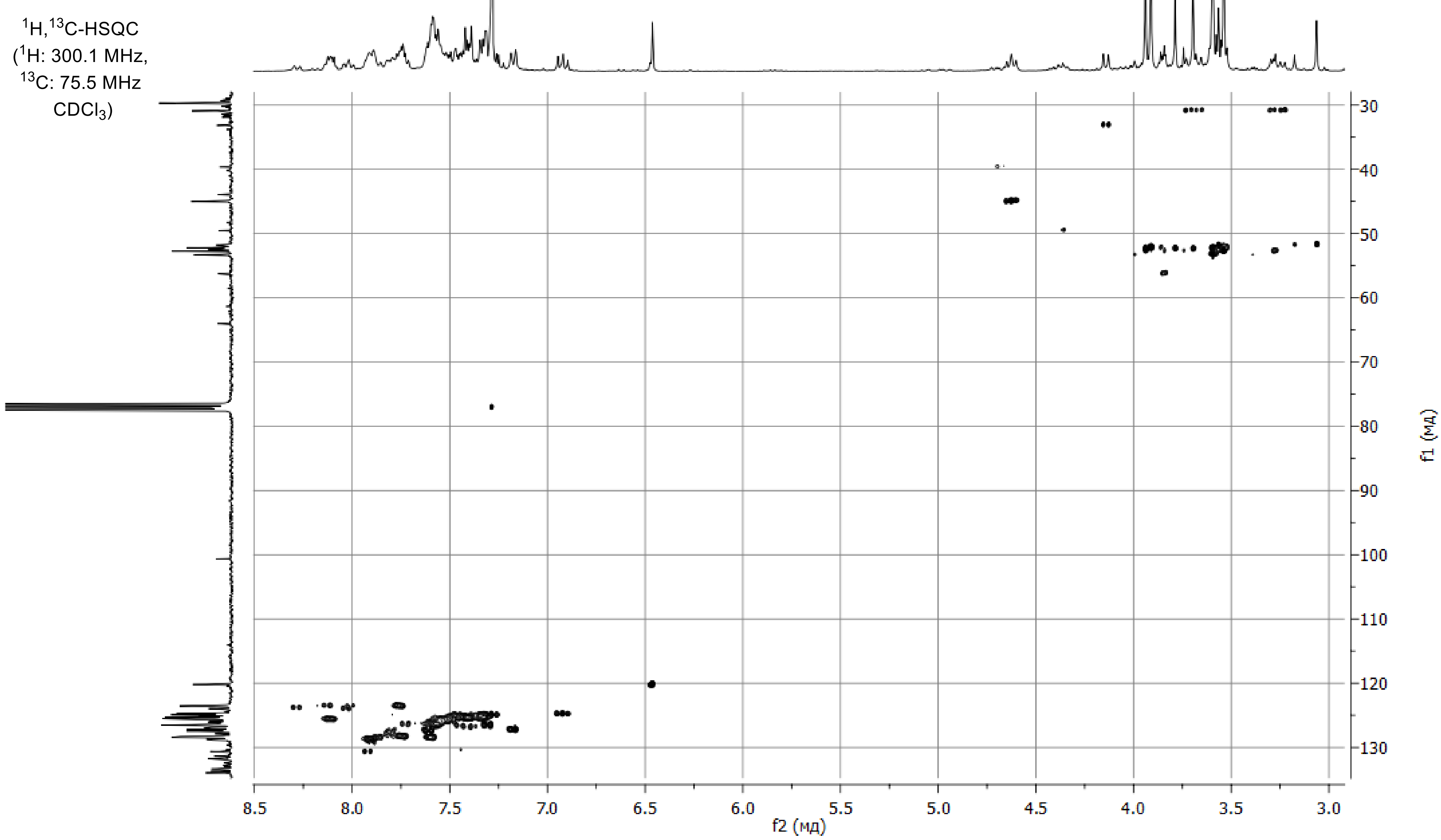

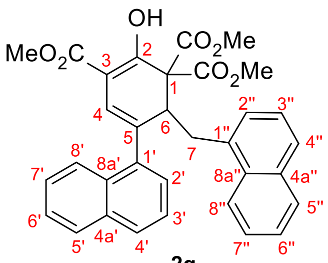

2g

$^{1}H,^{13}C$-HMBC
(^{1}H: 300.1 MHz,
^{13}C: 75.5 MHz
$CDCl_3$)

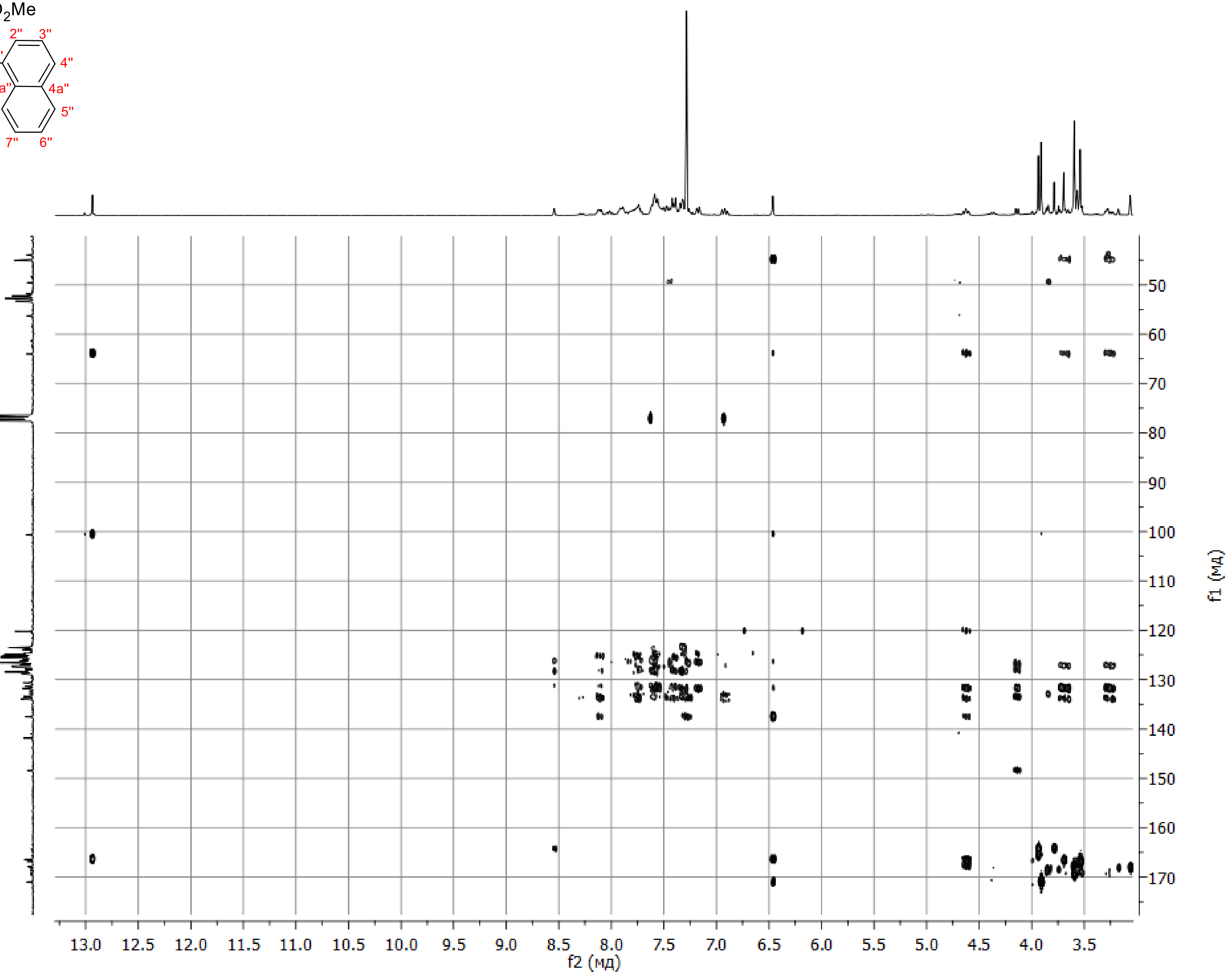

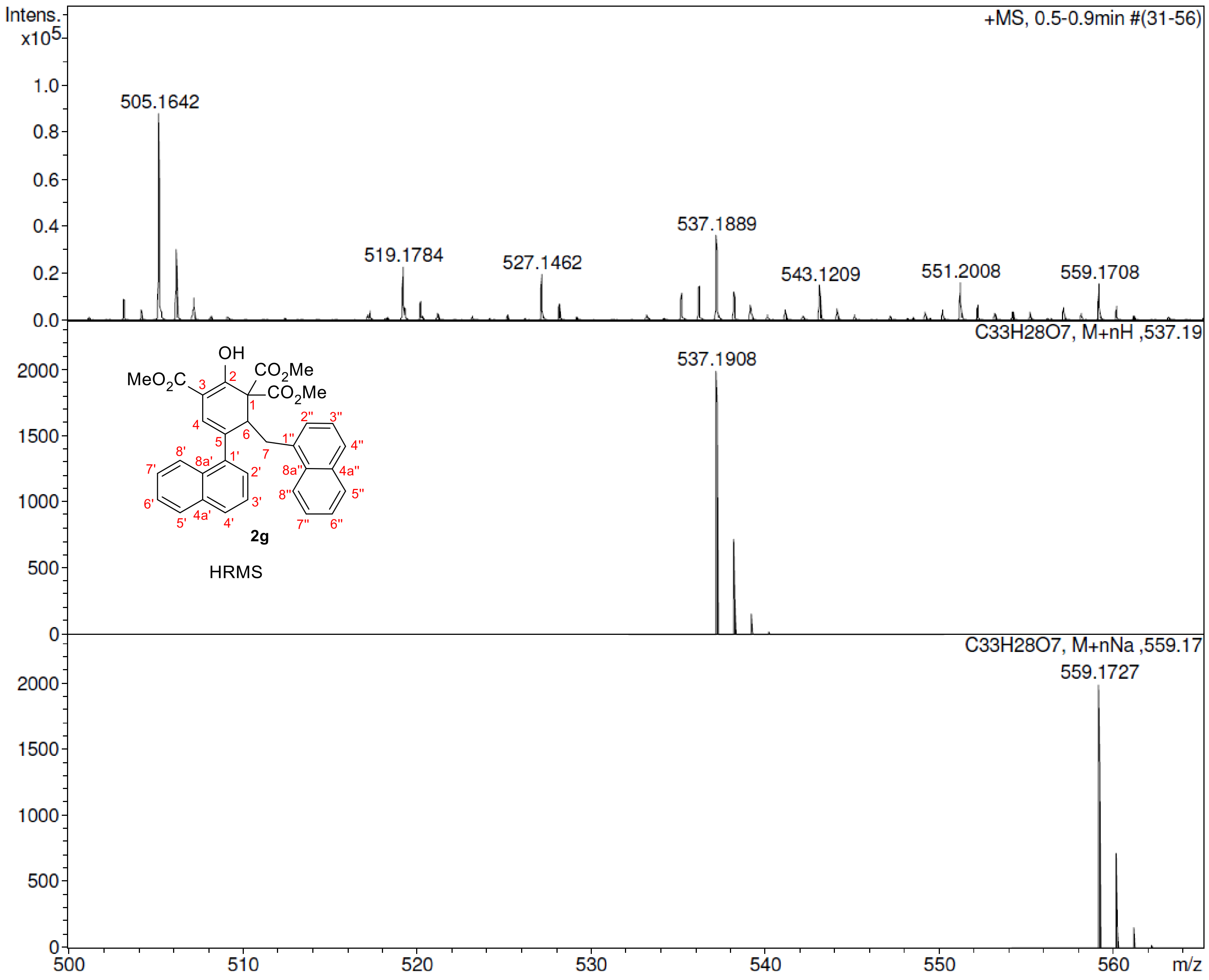
Intens.
x10^5
+MS, 0.5-0.9min #(31-56)
505.1642
519.1784
527.1462
537.1889
543.1209
551.2008
559.1708
C33H28O7, M+nH ,537.19
537.1908
C33H28O7, M+nNa ,559.17
559.1727
OH
MeO2C
CO2Me
CO2Me
2g
HRMS
m/z

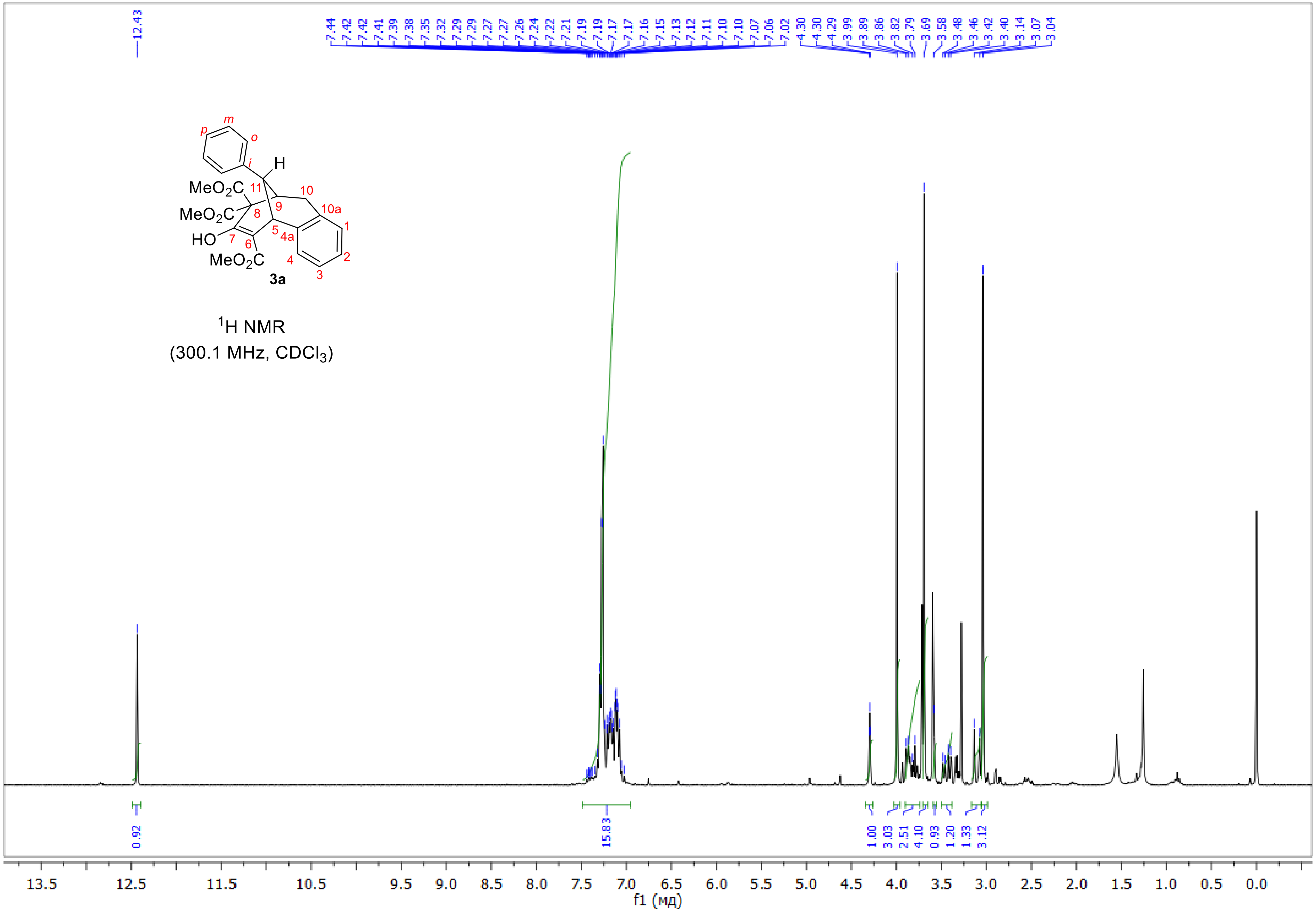

3a
^{1}H NMR
(300.1 MHz, CDCl3)
f1 (мд)

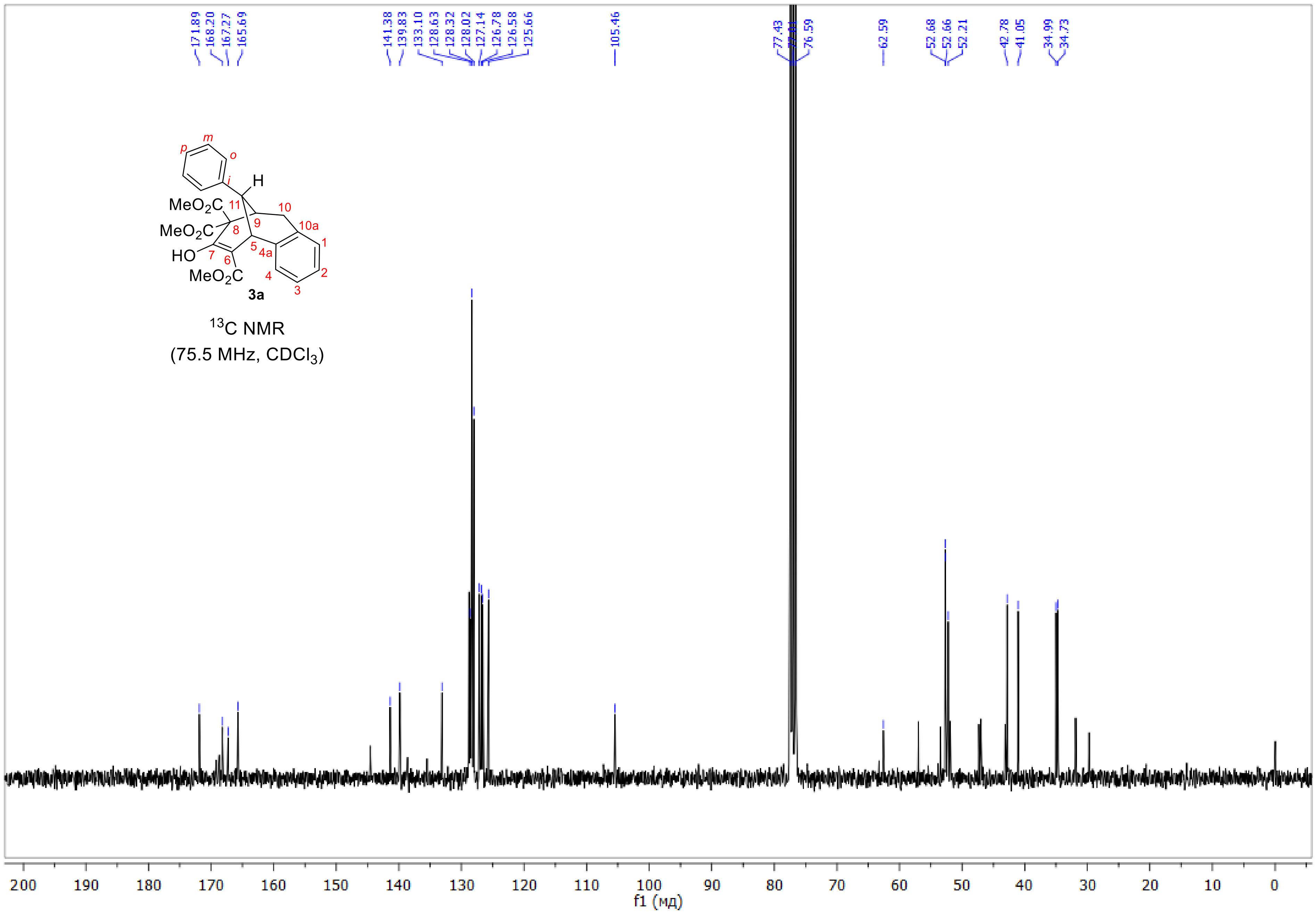

171.89
168.20
167.27
165.69
141.38
139.83
133.10
128.63
128.32
128.02
127.14
126.78
126.58
125.66
105.46
77.43
76.59
62.59
52.68
52.66
52.21
42.78
41.05
34.99
34.73
3a
^{13}C NMR
(75.5 MHz, $CDCl_3$)
f1 (мд)

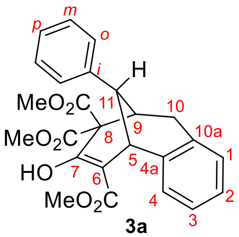

3a

$^{1}H,^{1}H$-COSY

(300.1 MHz, $CDCl_3$)

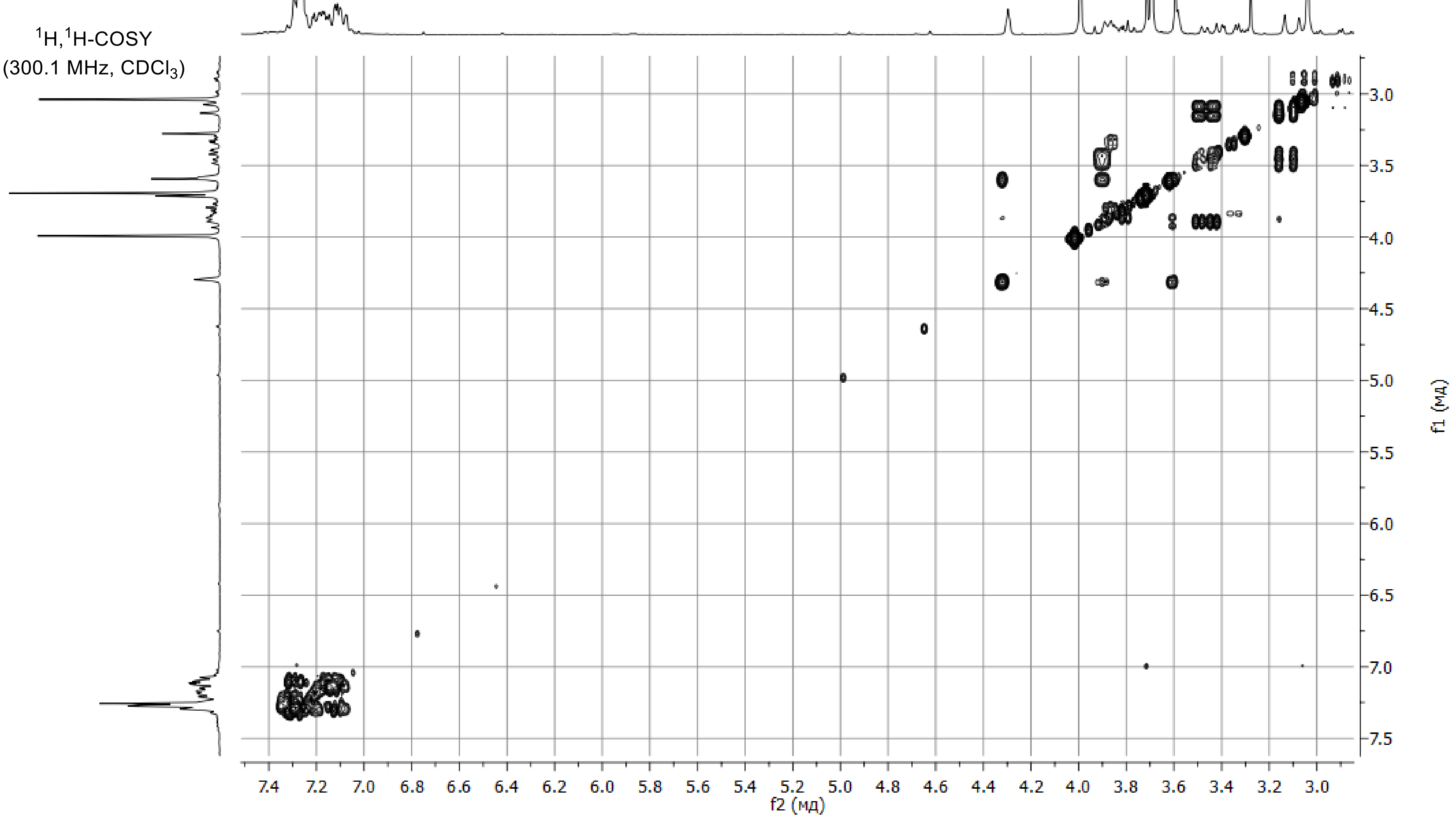

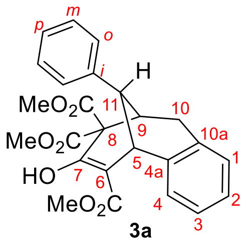

3a

$^{1}H,^{1}H$-NOESY
(300.1 MHz, $CDCl_3$)

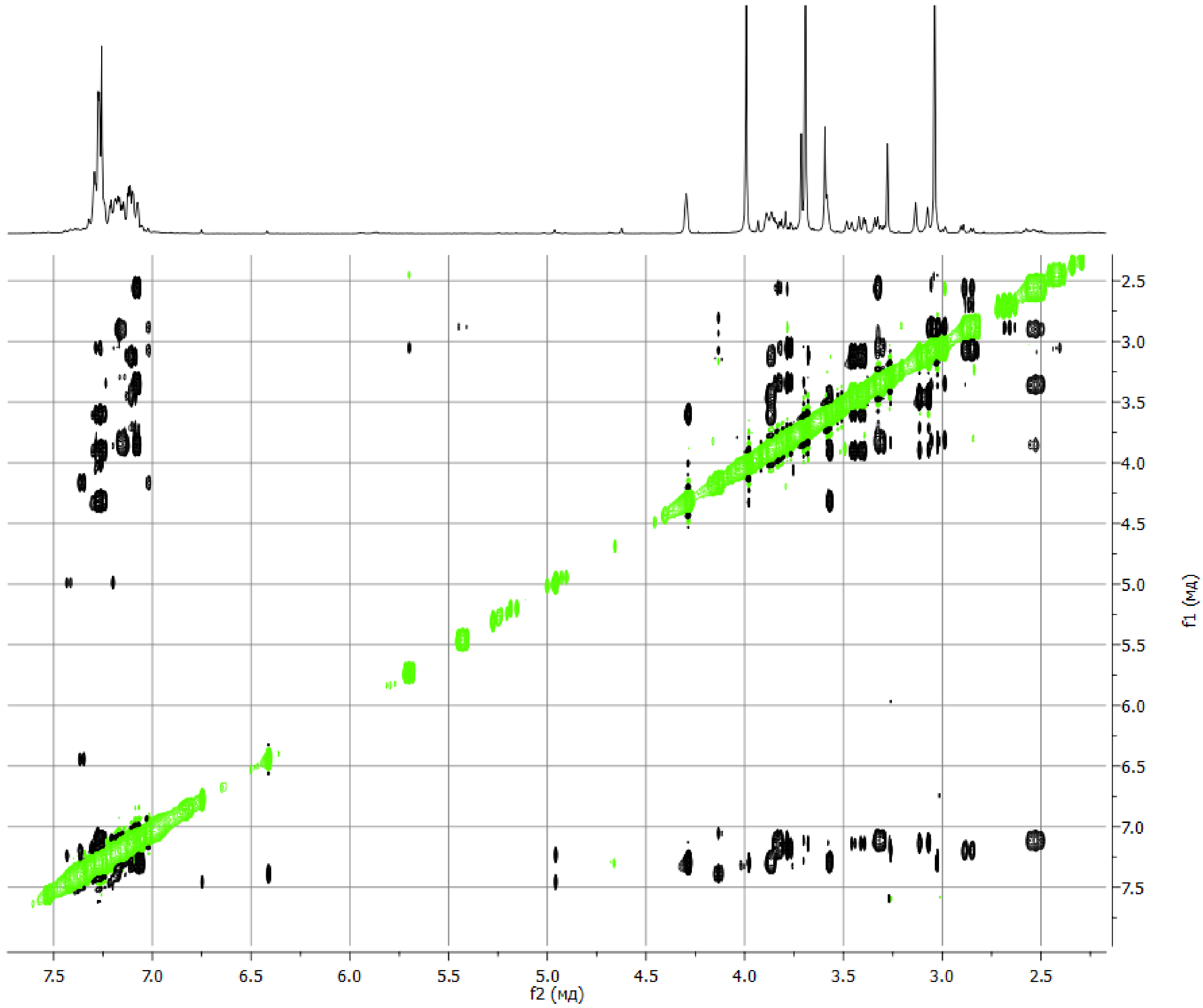

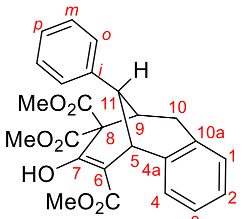

3a

$^{1}H,^{13}C$-HSQC
(^{1}H: 300.1 MHz,
^{13}C: 75.5 MHz
$CDCl_3$)

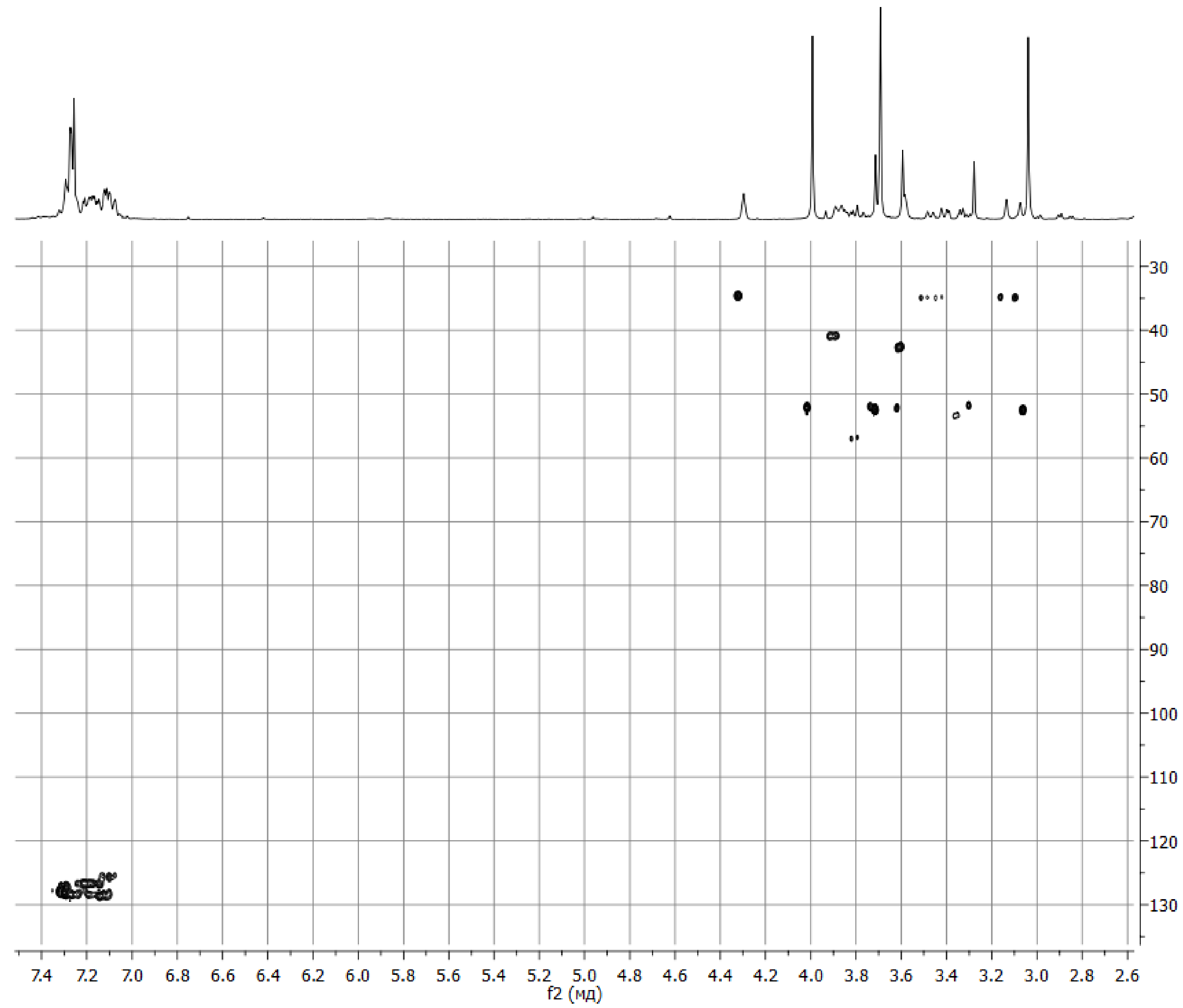

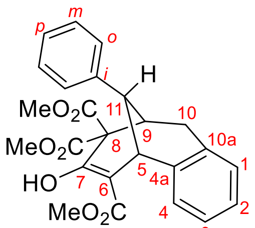

3a

$^1H,^{13}C$-HMBC
(^1H: 300.1 MHz,
^{13}C: 75.5 MHz
$CDCl_3$)

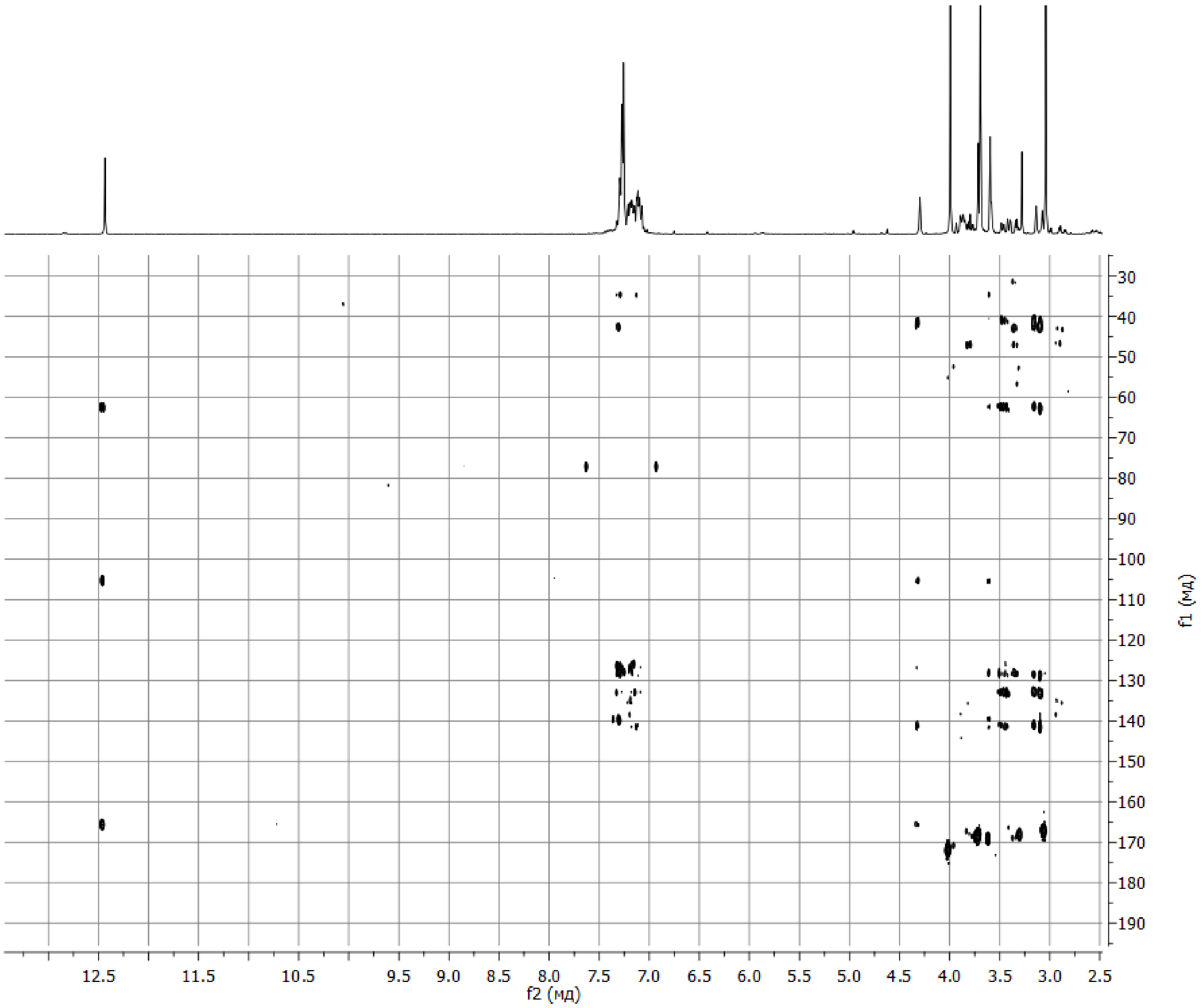

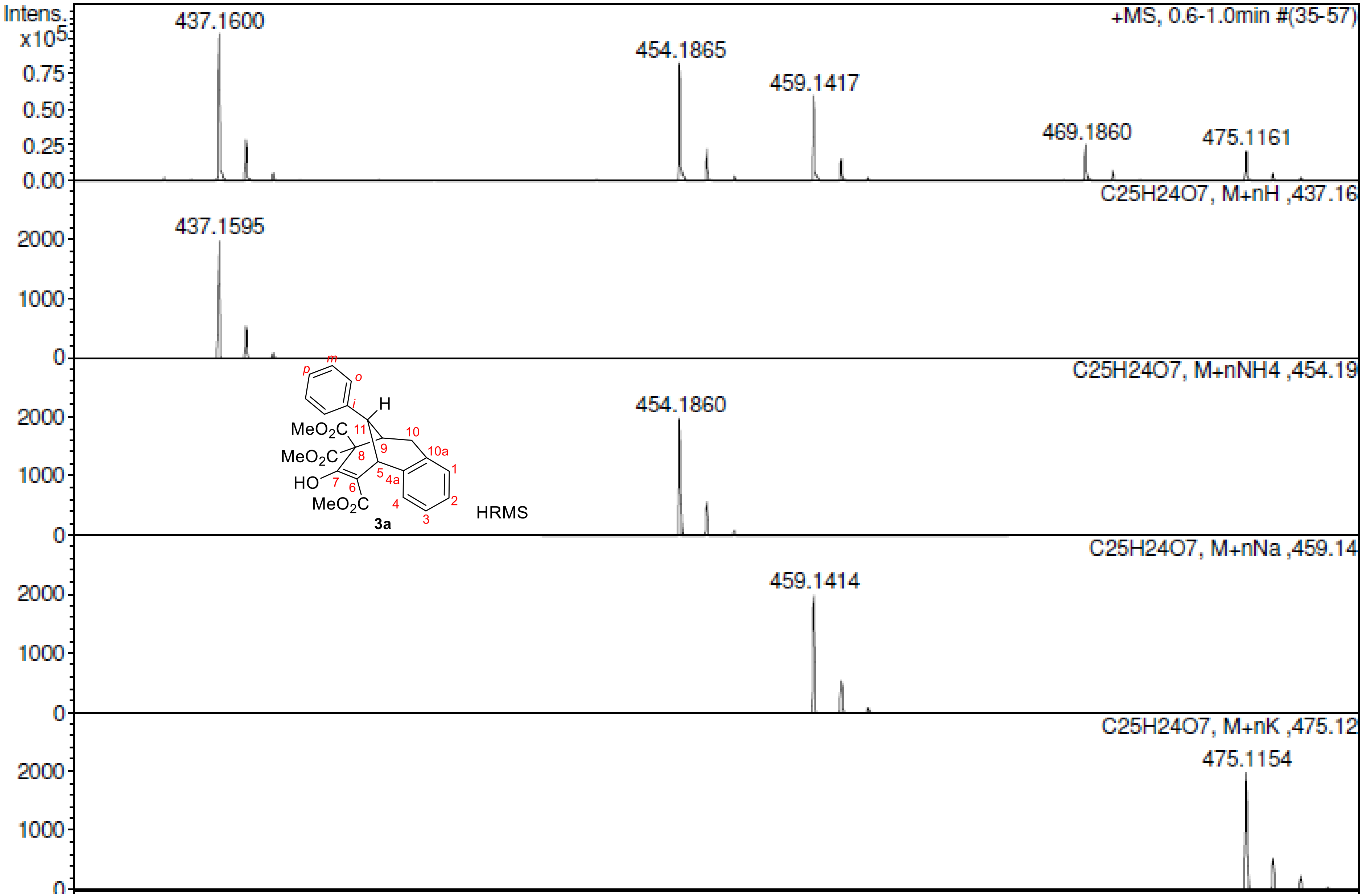
Intens.
x10^5
0.75
0.50
0.25
0.00
437.1600
454.1865
459.1417
469.1860
475.1161
+MS, 0.6-1.0min #(35-57)
C25H24O7, M+nH ,437.16
437.1595
2000
1000
0
C25H24O7, M+nNH4 ,454.19
454.1860
C25H24O7, M+nNa ,459.14
459.1414
C25H24O7, M+nK ,475.12
475.1154
MeO2C
MeO2C
HO
MeO2C
H
3a
HRMS

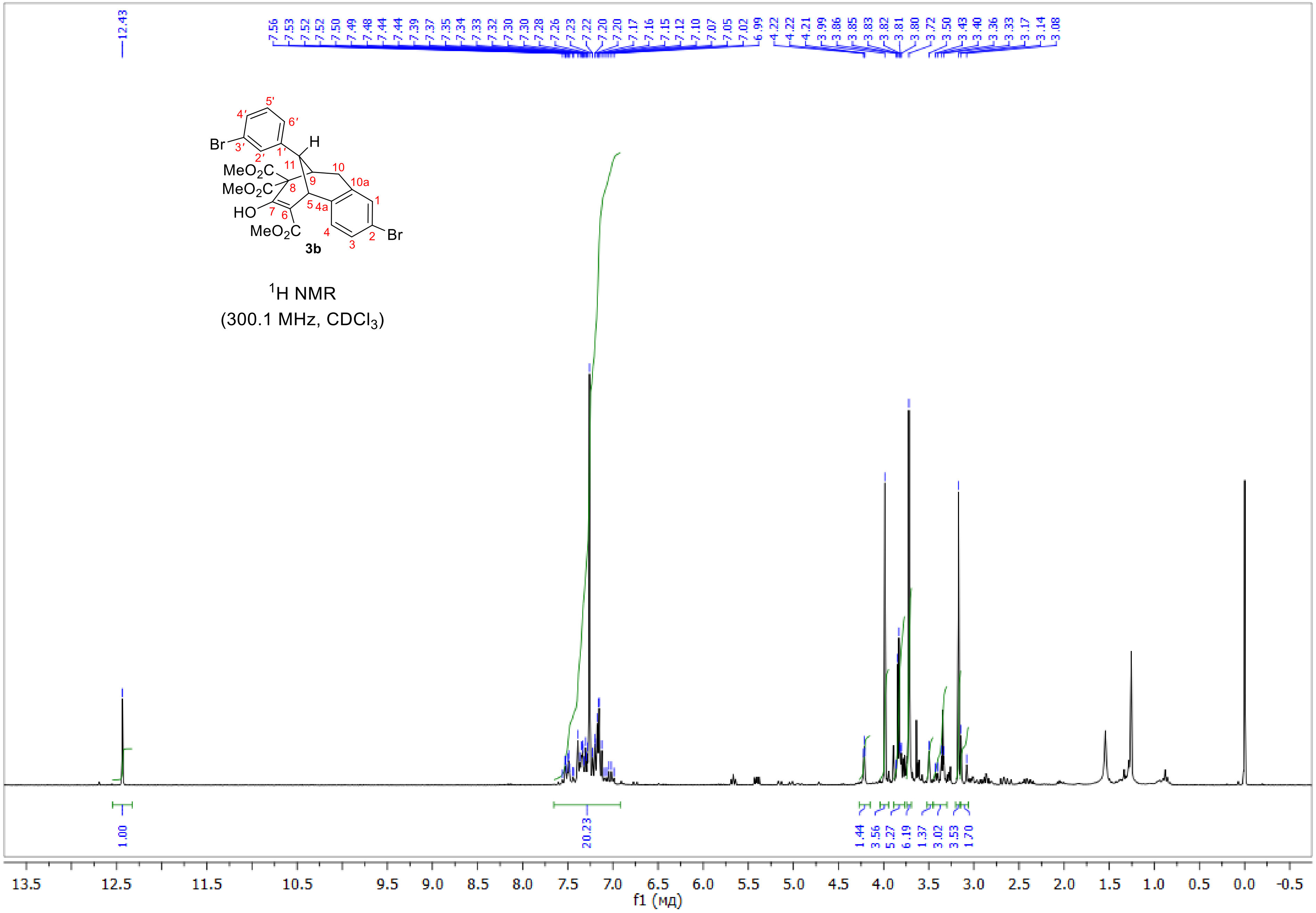

3b
^{1}H NMR
(300.1 MHz, CDCl$_3$)
f1 (мд)

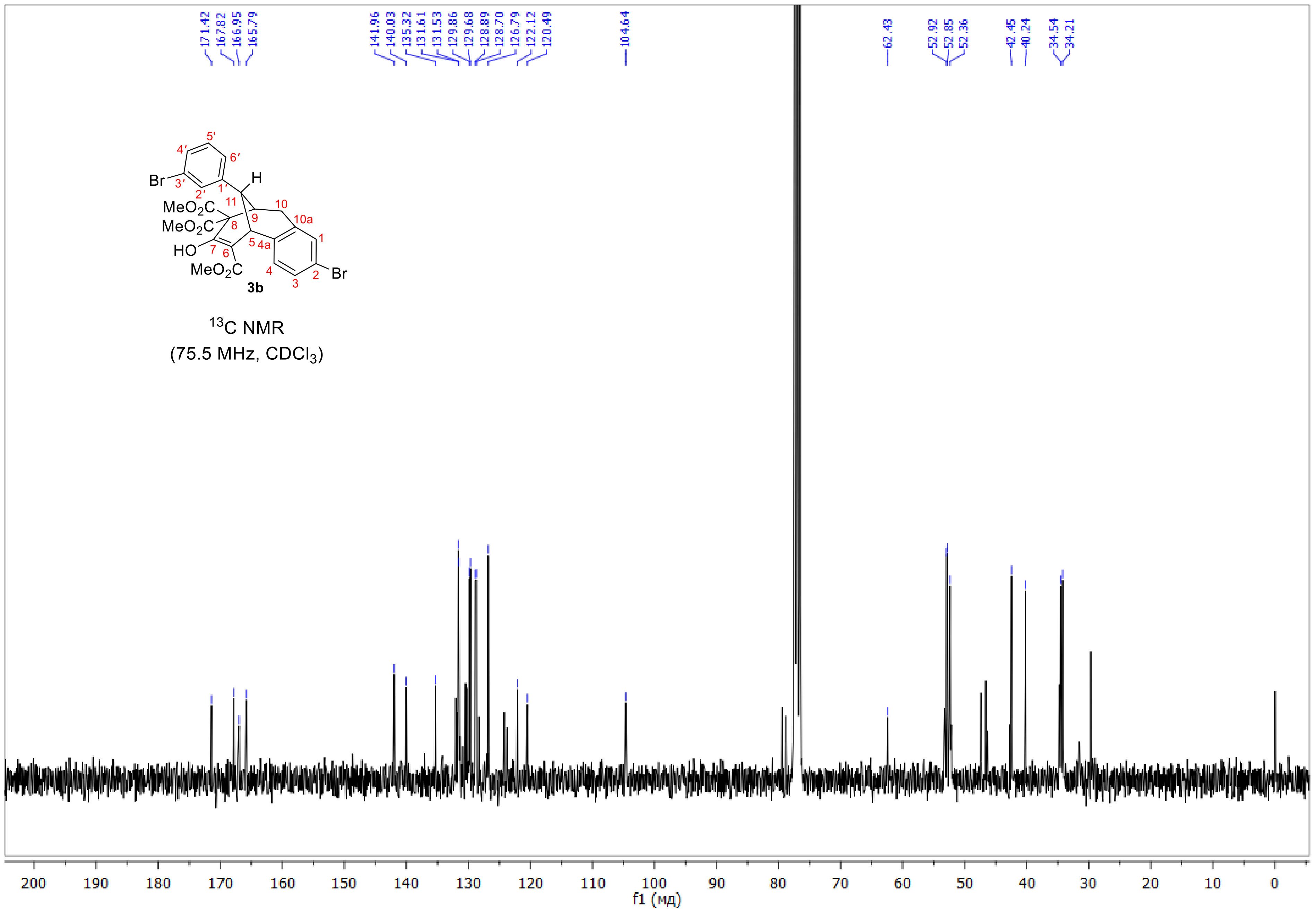
171.42
167.82
166.95
165.79
141.96
140.03
135.32
131.61
131.53
129.86
129.68
128.89
128.70
126.79
122.12
120.49
104.64
62.43
52.92
52.85
52.36
42.45
40.24
34.54
34.21
3b
13C NMR
(75.5 MHz, CDCl3)
f1 (мд)

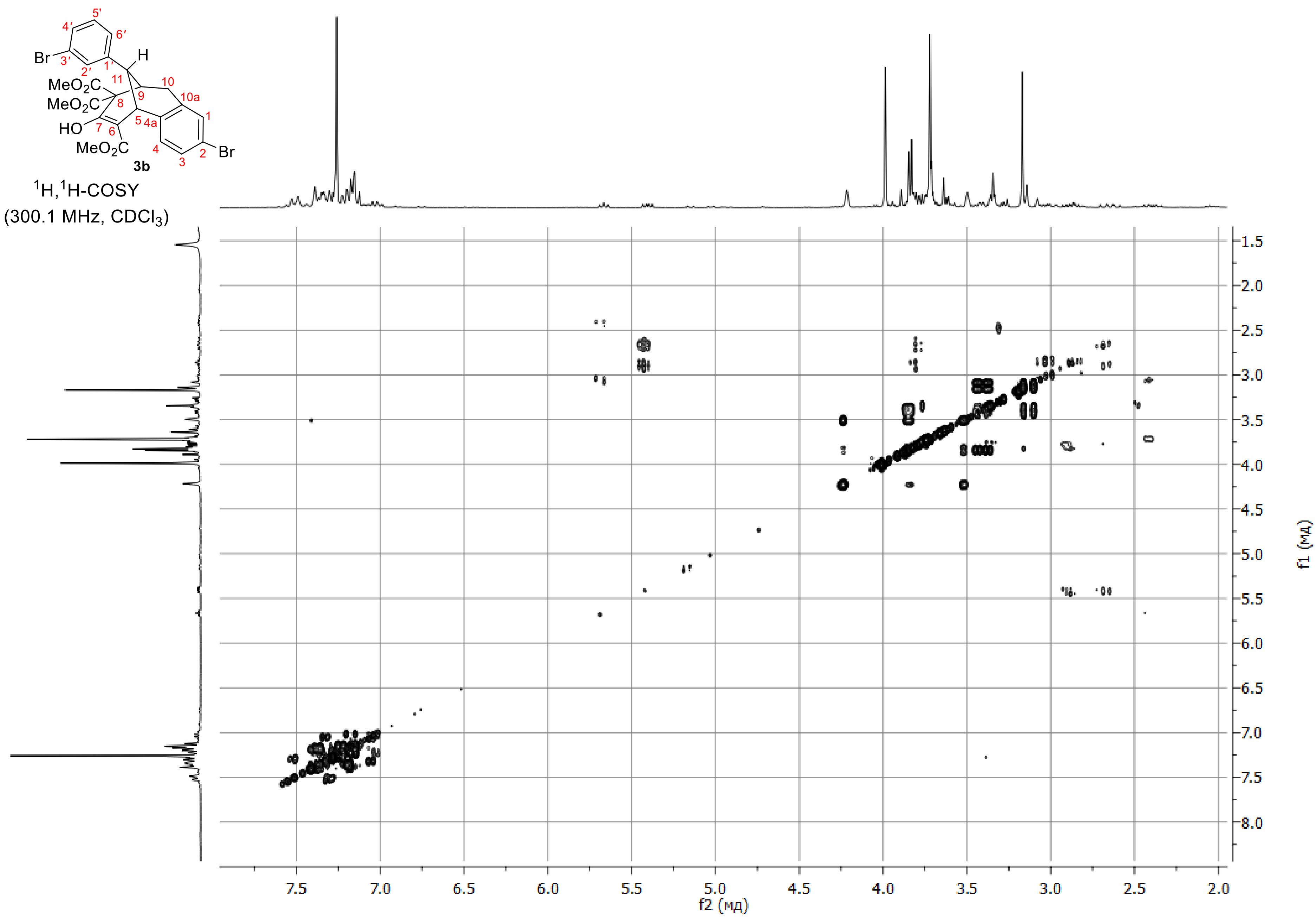

$^1H,^1H$-COSY

(300.1 MHz, $CDCl_3$)

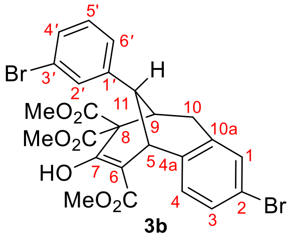

3b

$^{1}H,^{13}C$-HSQC
(^{1}H: 300.1 MHz,
^{13}C: 75.5 MHz
$CDCl_3$)

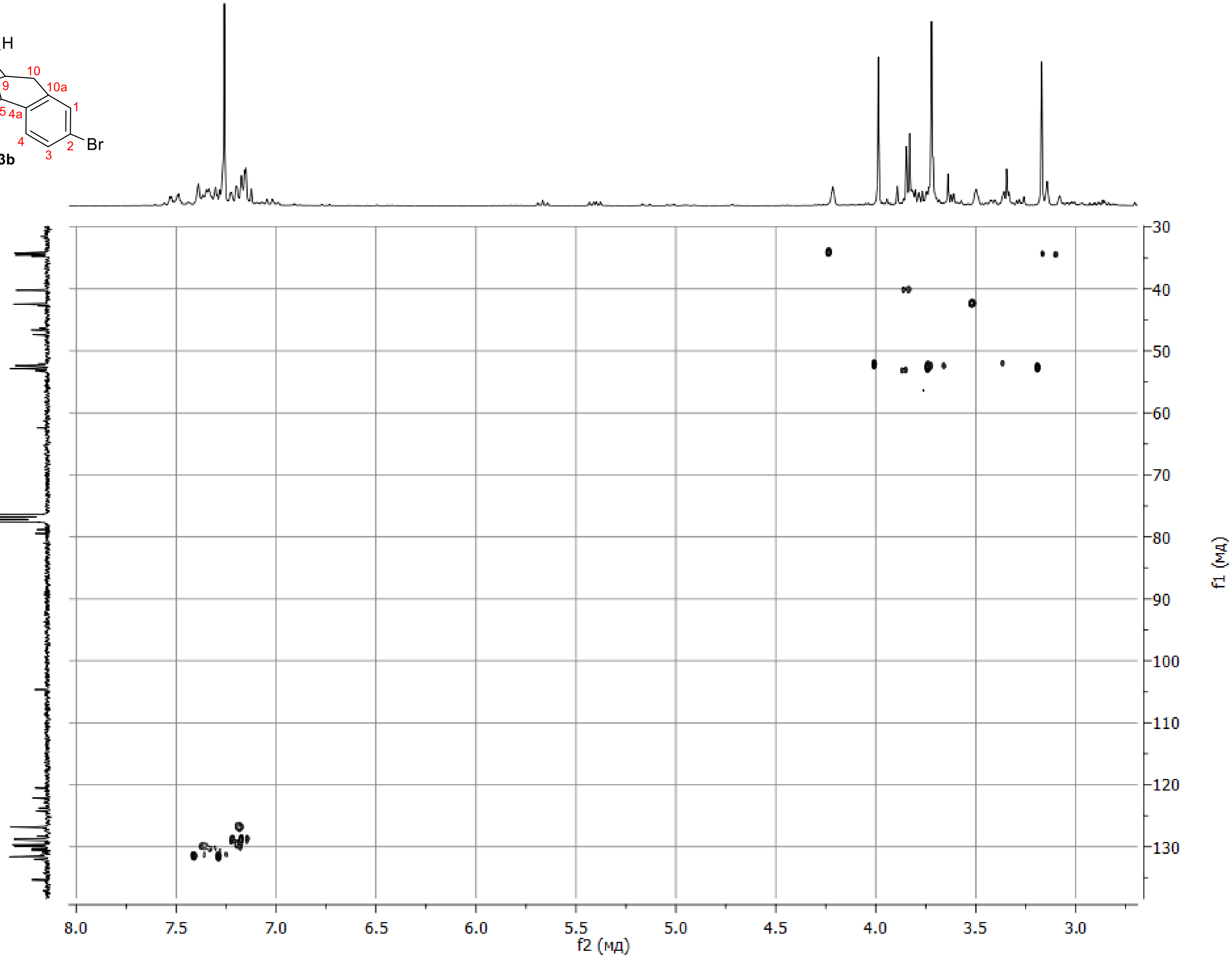

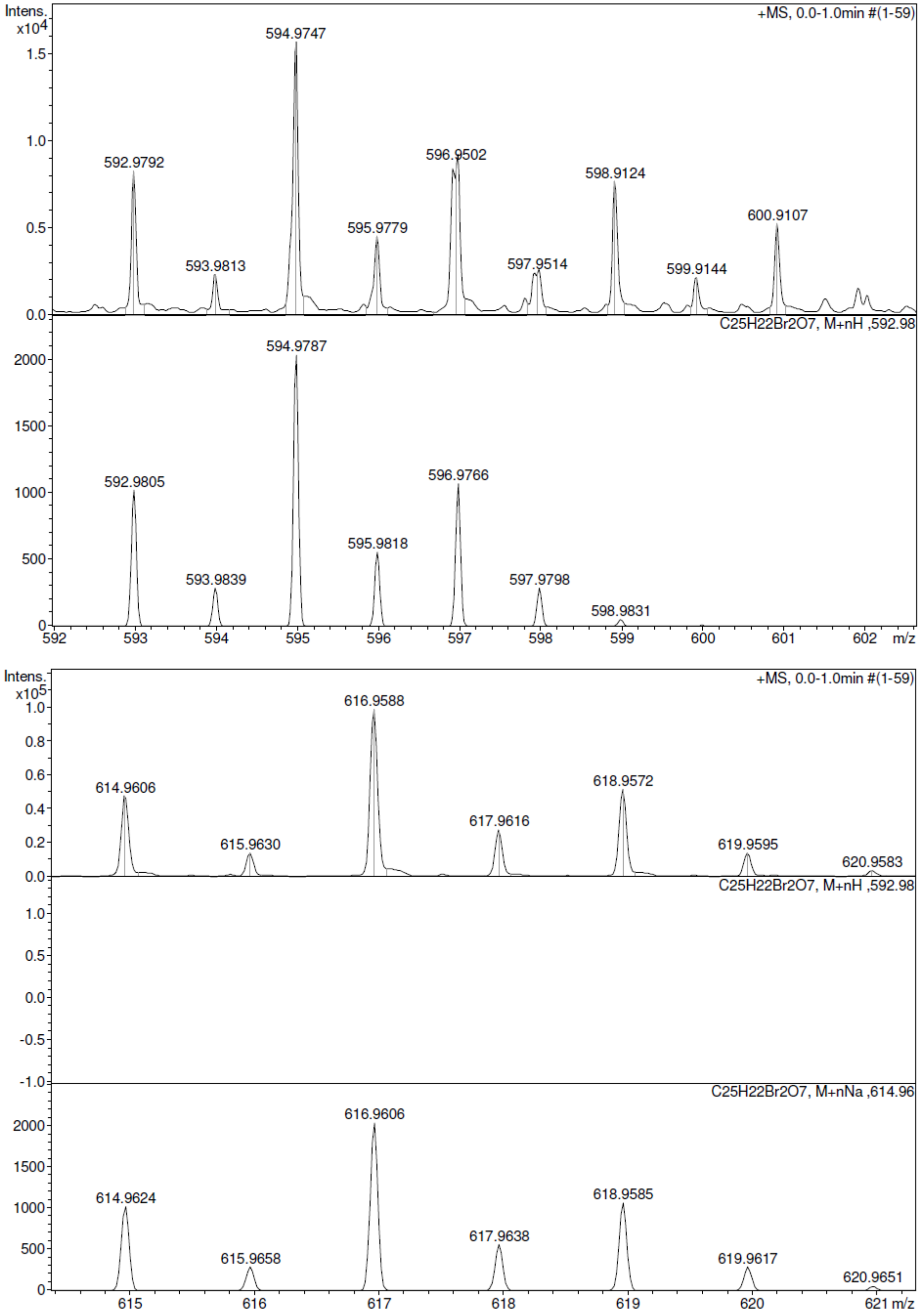
Intens.
x10^4
+MS, 0.0-1.0min #(1-59)
594.9747
592.9792
593.9813
595.9779
596.9502
597.9514
598.9124
599.9144
600.9107
C25H22Br2O7, M+nH ,592.98
594.9787
592.9805
593.9839
595.9818
596.9766
597.9798
598.9831
m/z
Intens.
x10^5
+MS, 0.0-1.0min #(1-59)
616.9588
614.9606
615.9630
617.9616
618.9572
619.9595
620.9583
C25H22Br2O7, M+nH ,592.98
C25H22Br2O7, M+nNa ,614.96
616.9606
614.9624
615.9658
617.9638
618.9585
619.9617
620.9651
m/z

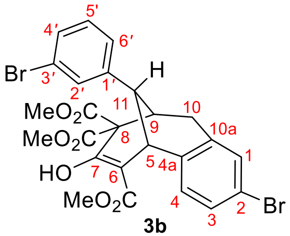
Br
H
MeO2C
MeO2C
HO
MeO2C
Br

3b

HRMS

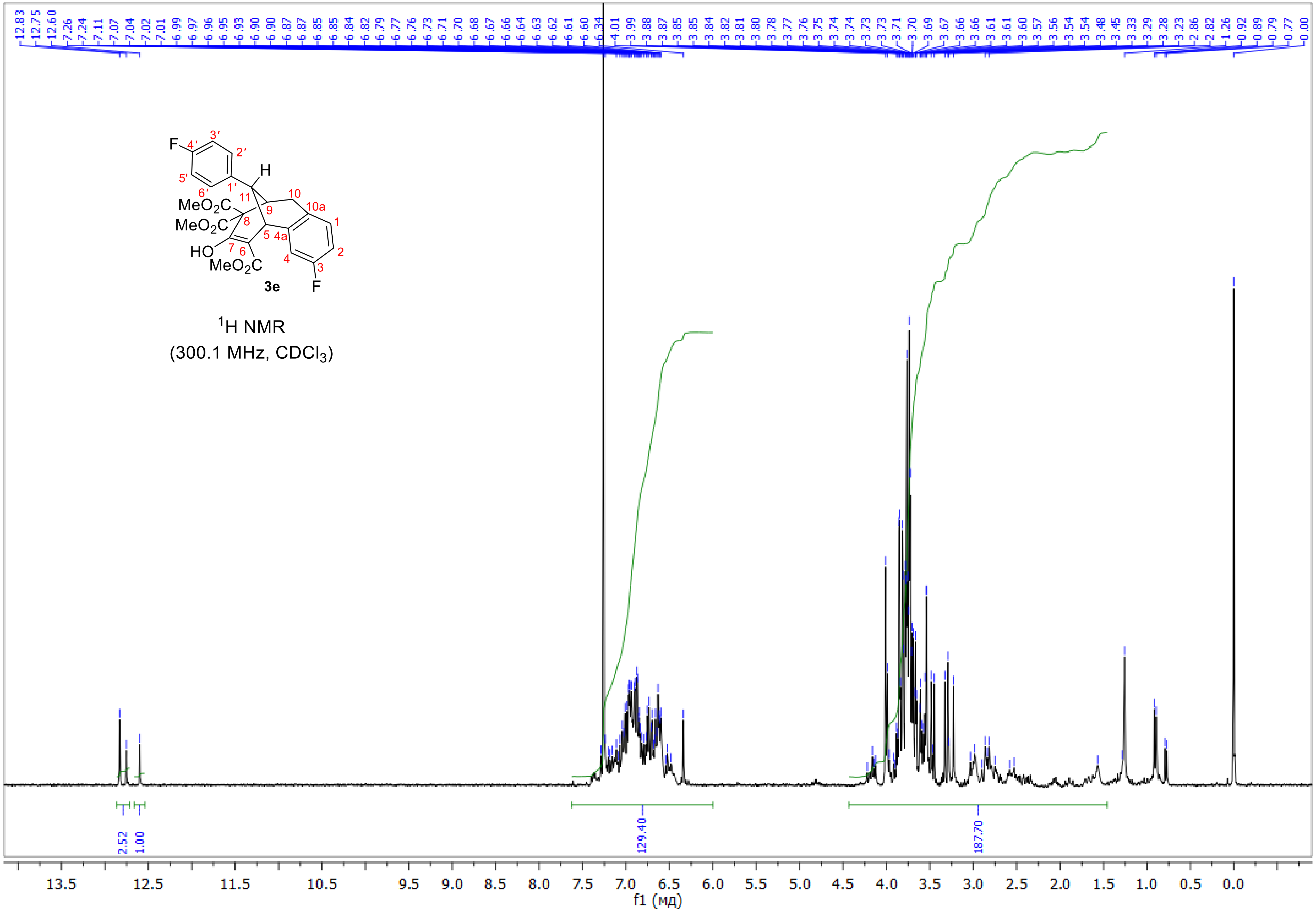

3e
^{1}H NMR
(300.1 MHz, $CDCl_3$)
f1 (мд)

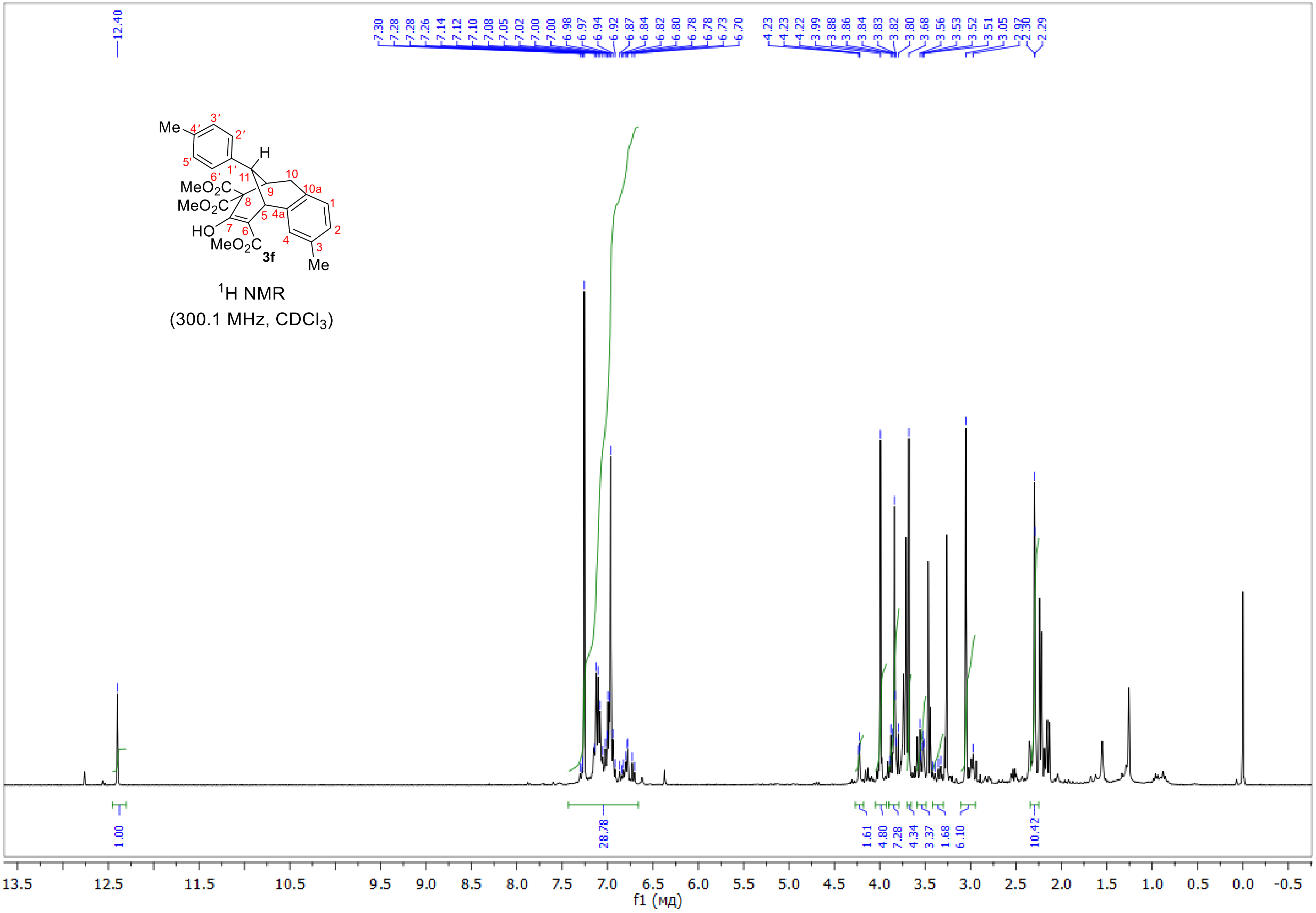
3f
1H NMR
(300.1 MHz, CDCl3)
f1 (мд)

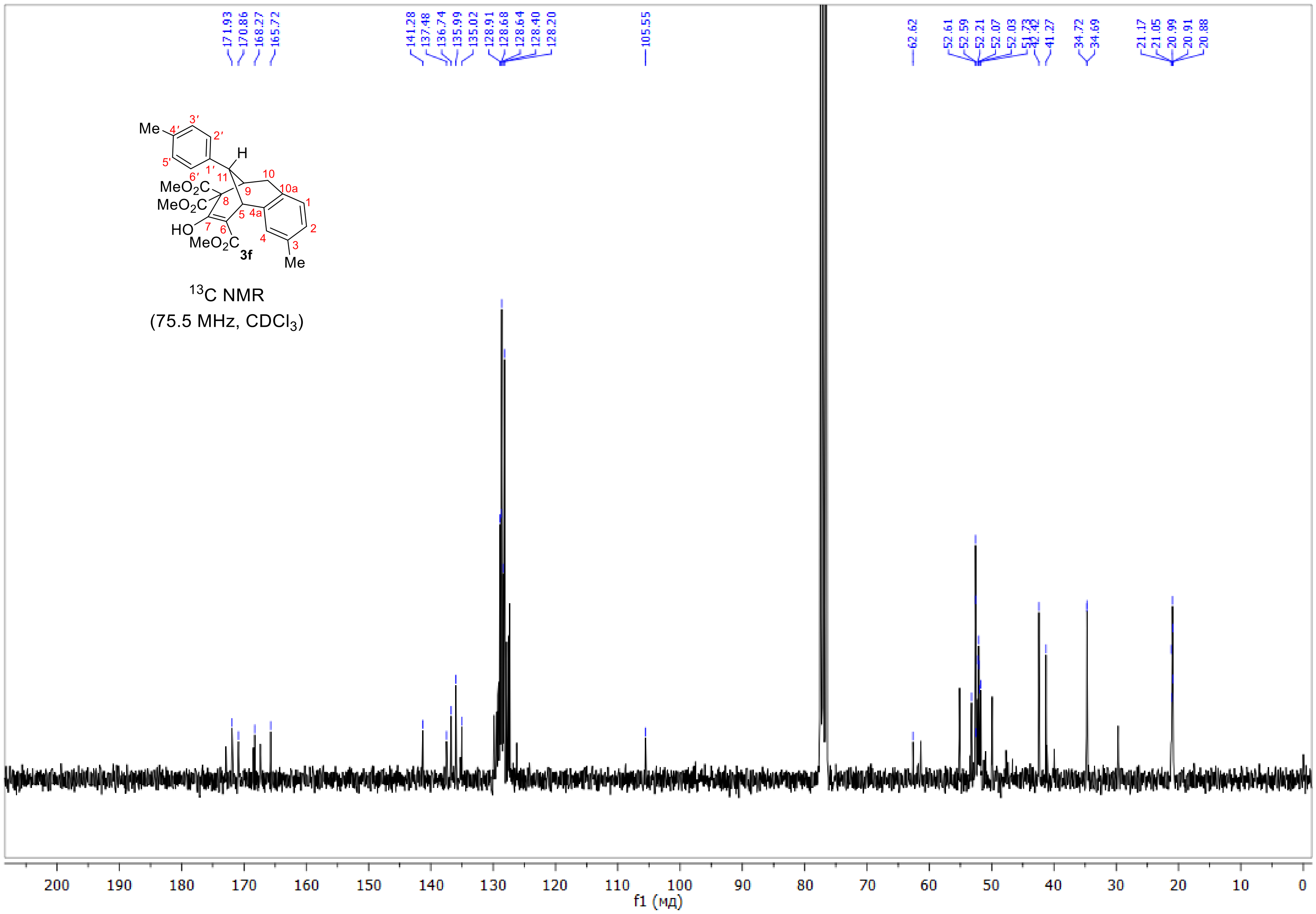

171.93
170.86
168.27
165.72
141.28
137.48
136.74
135.99
135.02
128.91
128.68
128.64
128.40
128.20
105.55
62.62
52.61
52.59
52.21
52.07
52.03
41.27
34.72
34.69
21.17
21.05
20.99
20.91
20.88
Me
4′
3′
2′
H
5′
6′
1′
11
10
MeO2C
9
10a
1
MeO2C
8
5
4a
HO
7
6
2
MeO2C
4
3
3f
Me
13C NMR
(75.5 MHz, CDCl3)
200
190
180
170
160
150
140
130
120
110
100
90
80
70
60
50
40
30
20
10
0
f1 (мд)

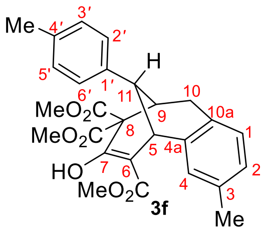
3f

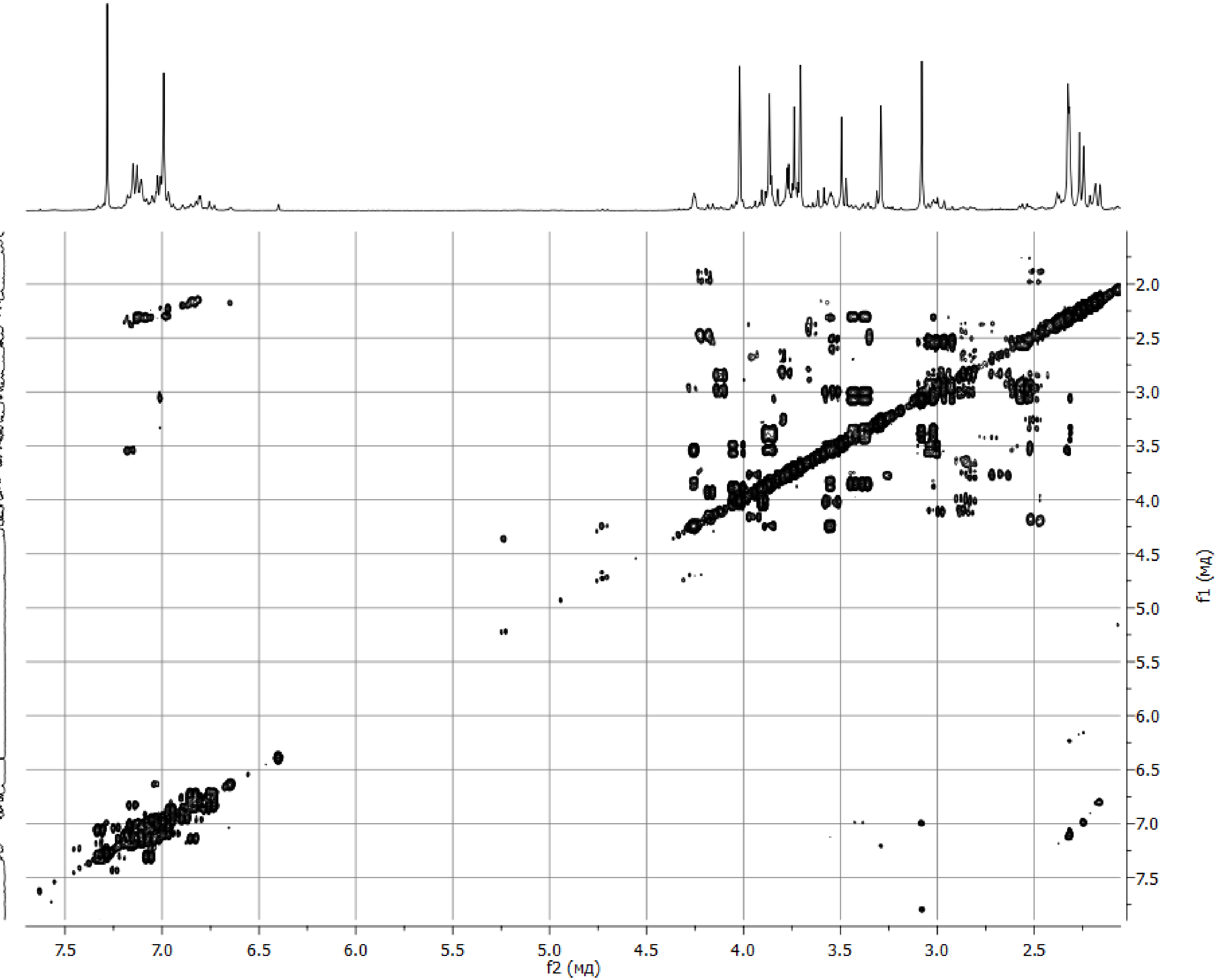
^{1}H,^{1}H-COSY
(300.1 MHz, CDCl$_3$)
f2 (мд)
f1 (мд)

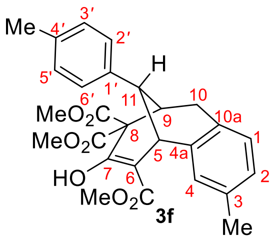

$^{1}H,^{13}C$-HSQC
(^{1}H: 300.1 MHz,
^{13}C: 75.5 MHz
$CDCl_3$)

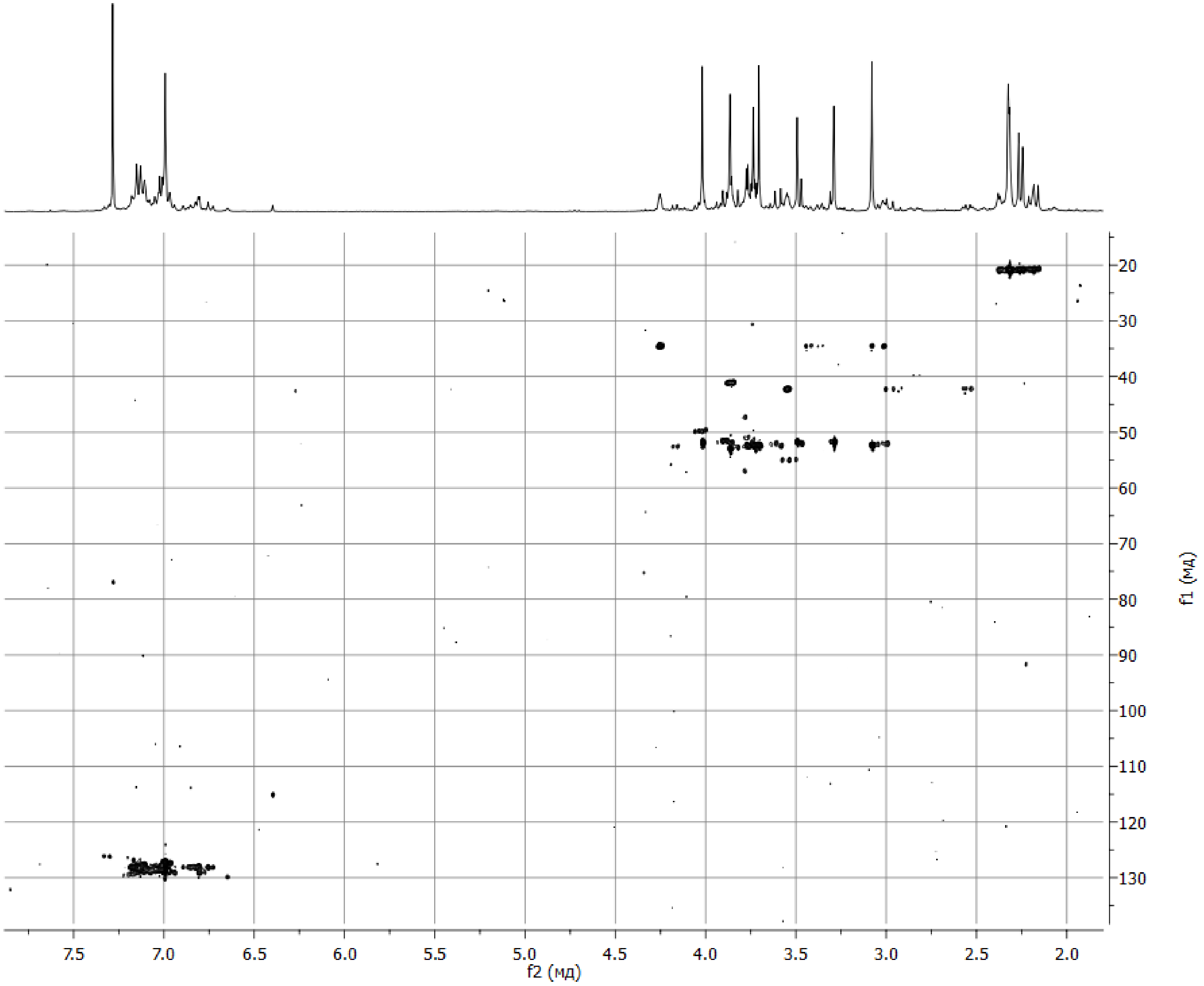

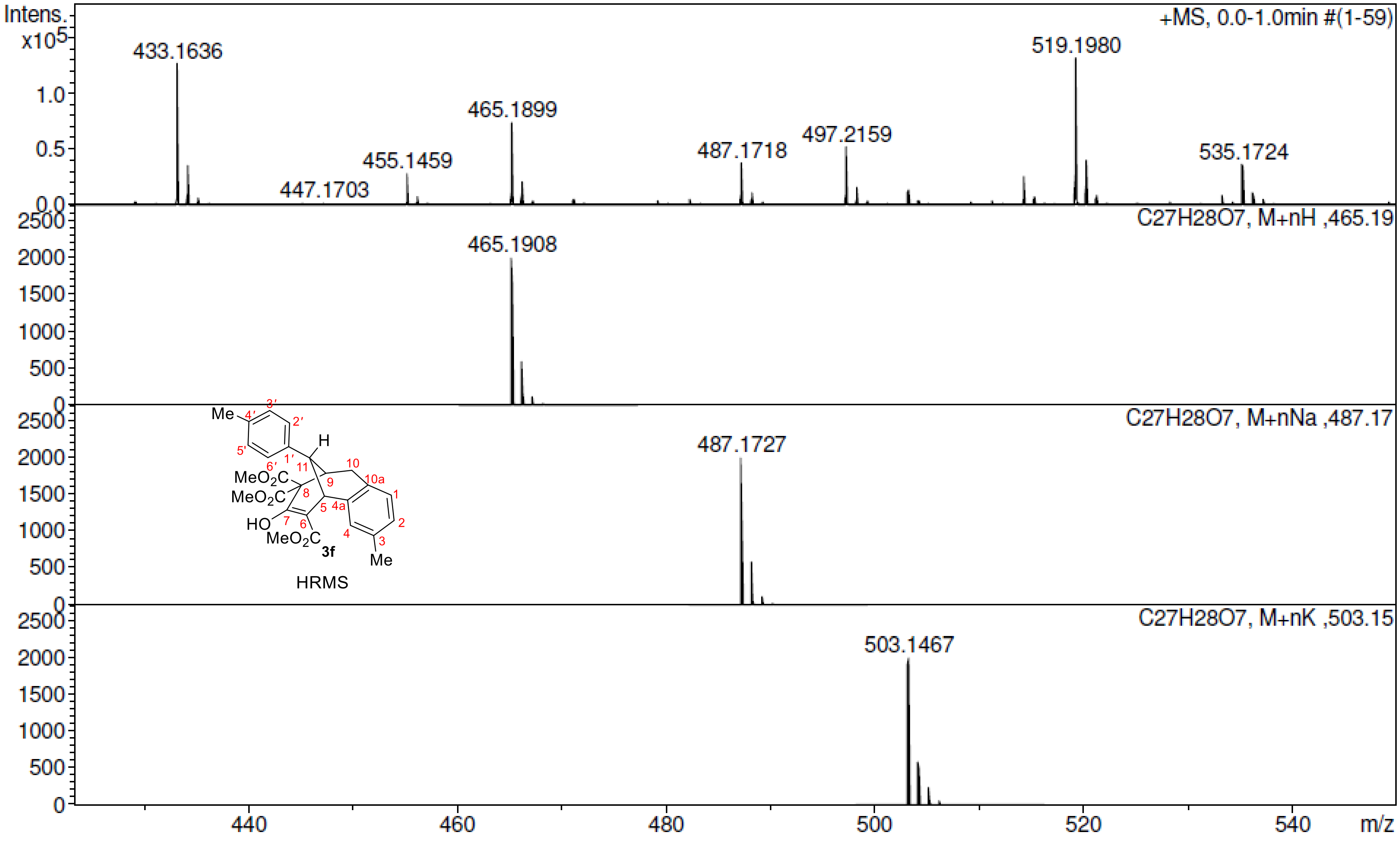

Intens.
x10^5
+MS, 0.0-1.0min #(1-59)
433.1636
447.1703
455.1459
465.1899
487.1718
497.2159
519.1980
535.1724
C27H28O7, M+nH ,465.19
465.1908
C27H28O7, M+nNa ,487.17
487.1727
C27H28O7, M+nK ,503.15
503.1467
3f
HRMS
m/z

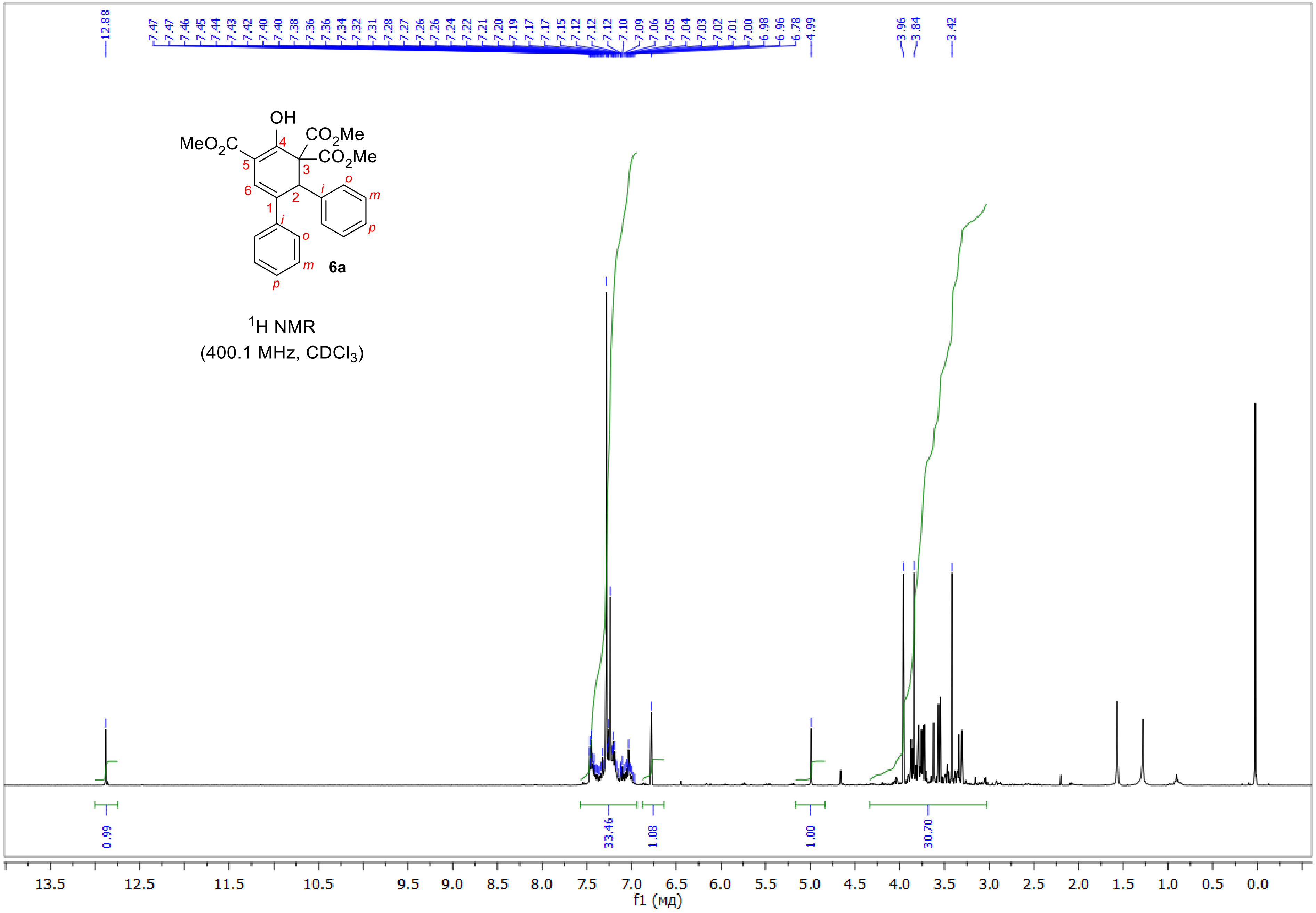

OH
MeO2C
CO2Me
CO2Me
6a
1H NMR
(400.1 MHz, CDCl3)
12.88
4.99
3.96
3.84
3.42
0.99
33.46
1.08
1.00
30.70
f1 (мд)

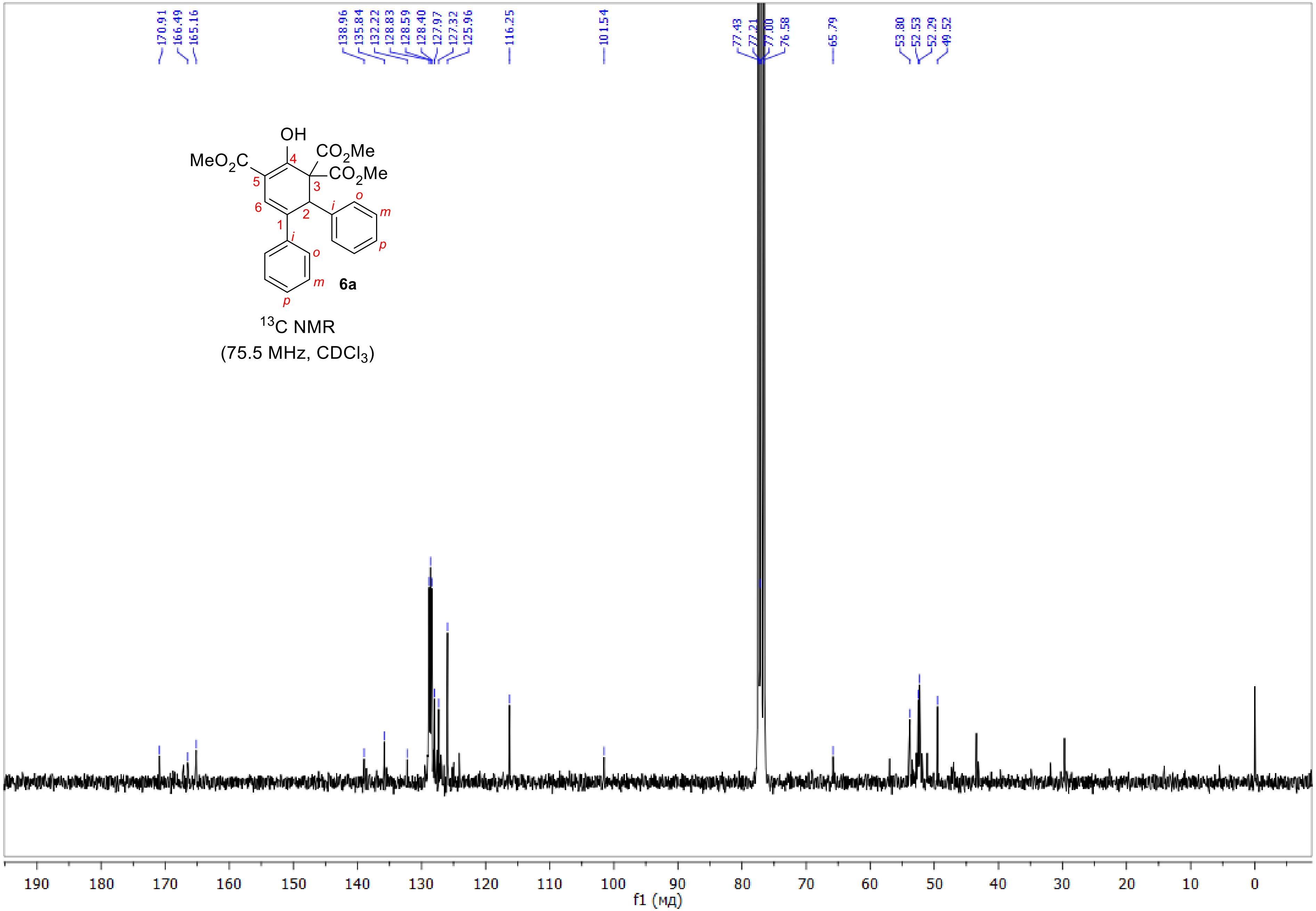
170.91
166.49
165.16
138.96
135.84
132.22
128.83
128.59
128.40
127.97
127.32
125.96
116.25
101.54
77.43
77.21
77.00
76.58
65.79
53.80
52.53
52.29
49.52
OH
CO_2Me
MeO_2C
CO_2Me
6a
^{13}C NMR
(75.5 MHz, $CDCl_3$)
190
180
170
160
150
140
130
120
110
100
90
80
70
60
50
40
30
20
10
0
f1 (мд)

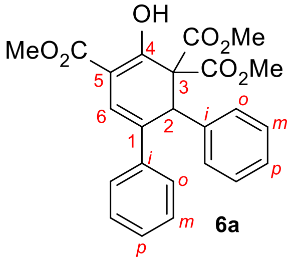

6a

$^{1}H,^{13}C$-HSQC
(^{1}H: 300.1 MHz,
^{13}C: 75.5 MHz
$CDCl_3$)

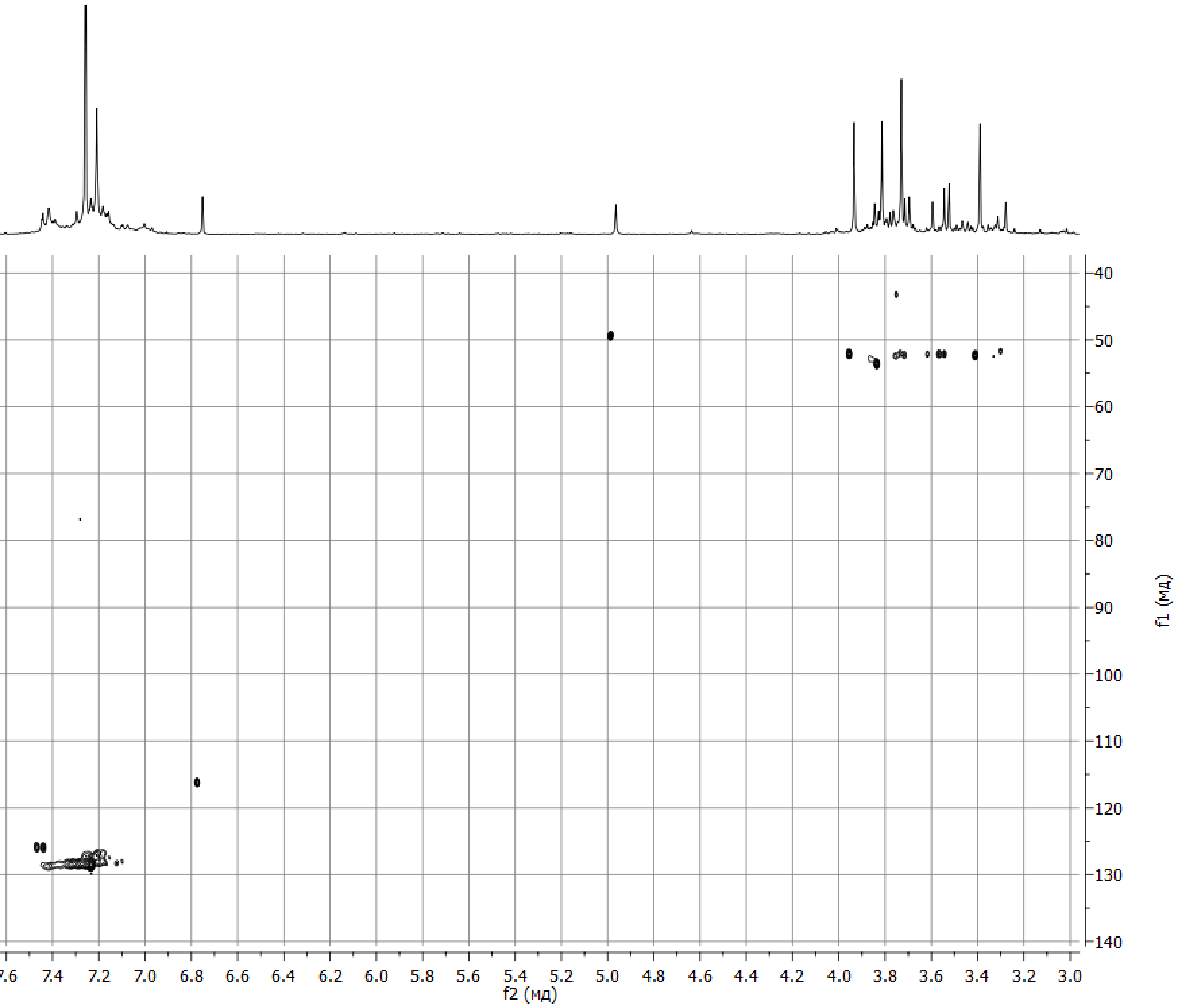

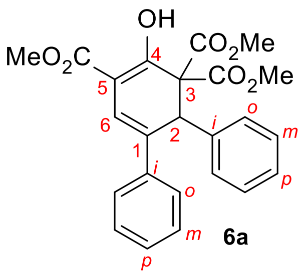

^{1}H,^{13}C-HMBC
(^{1}H: 300.1 MHz,
^{13}C: 75.5 MHz
$CDCl_3$)

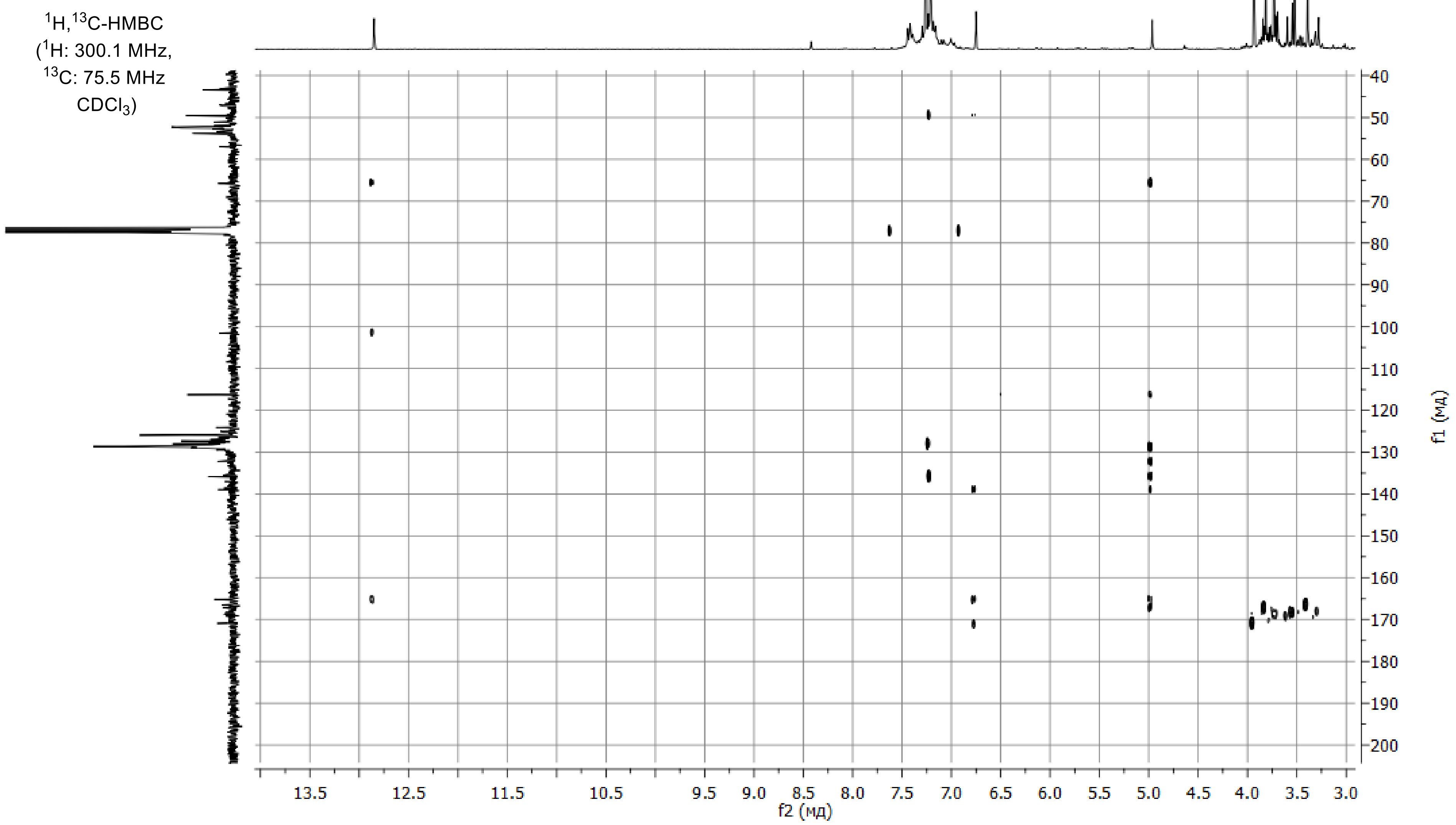

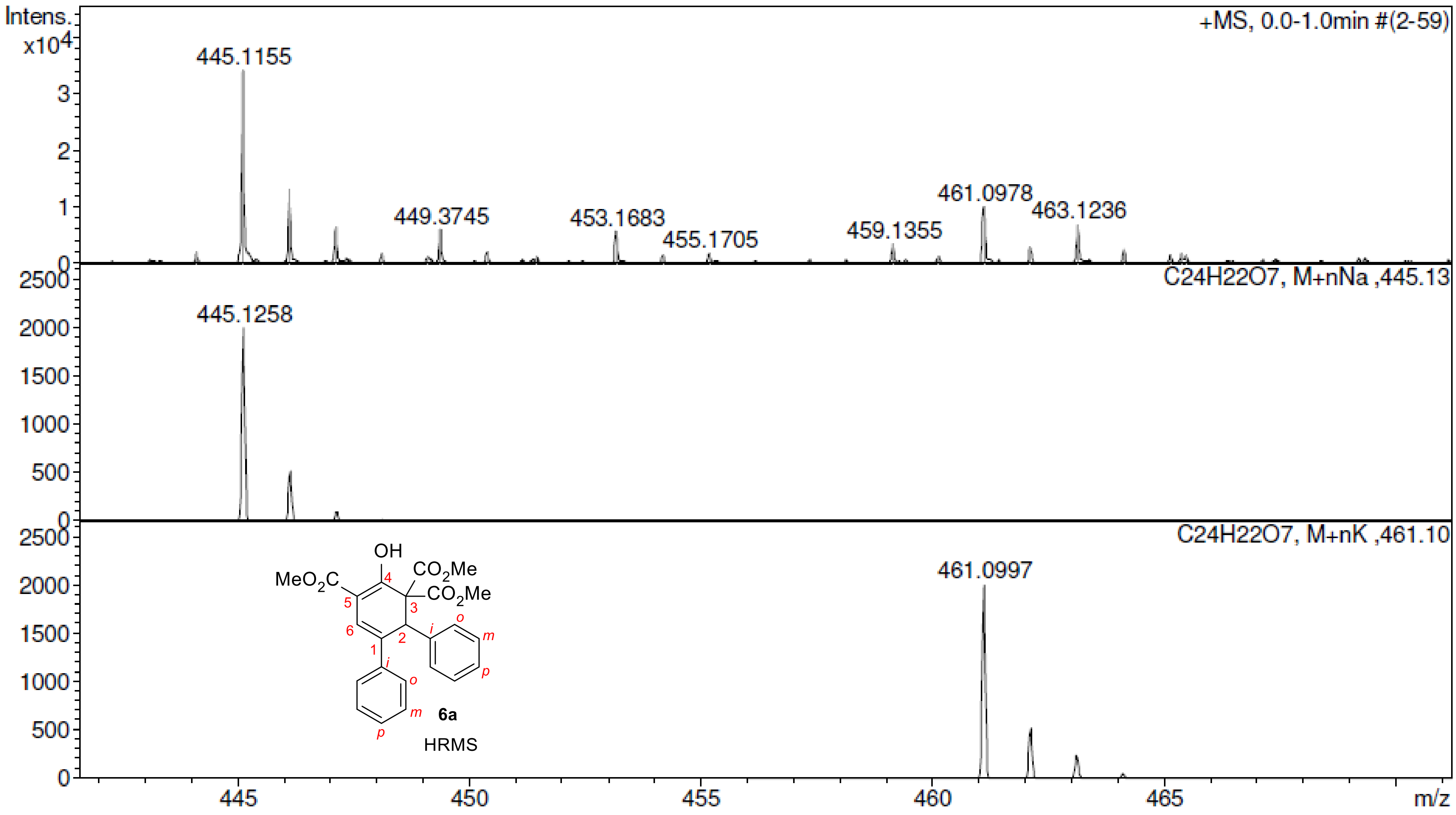

Intens.
x10^4
+MS, 0.0-1.0min #(2-59)
445.1155
449.3745
453.1683
455.1705
459.1355
461.0978
463.1236
C24H22O7, M+nNa ,445.13
445.1258
C24H22O7, M+nK ,461.10
461.0997
OH
CO2Me
MeO2C
CO2Me
6a
HRMS
m/z

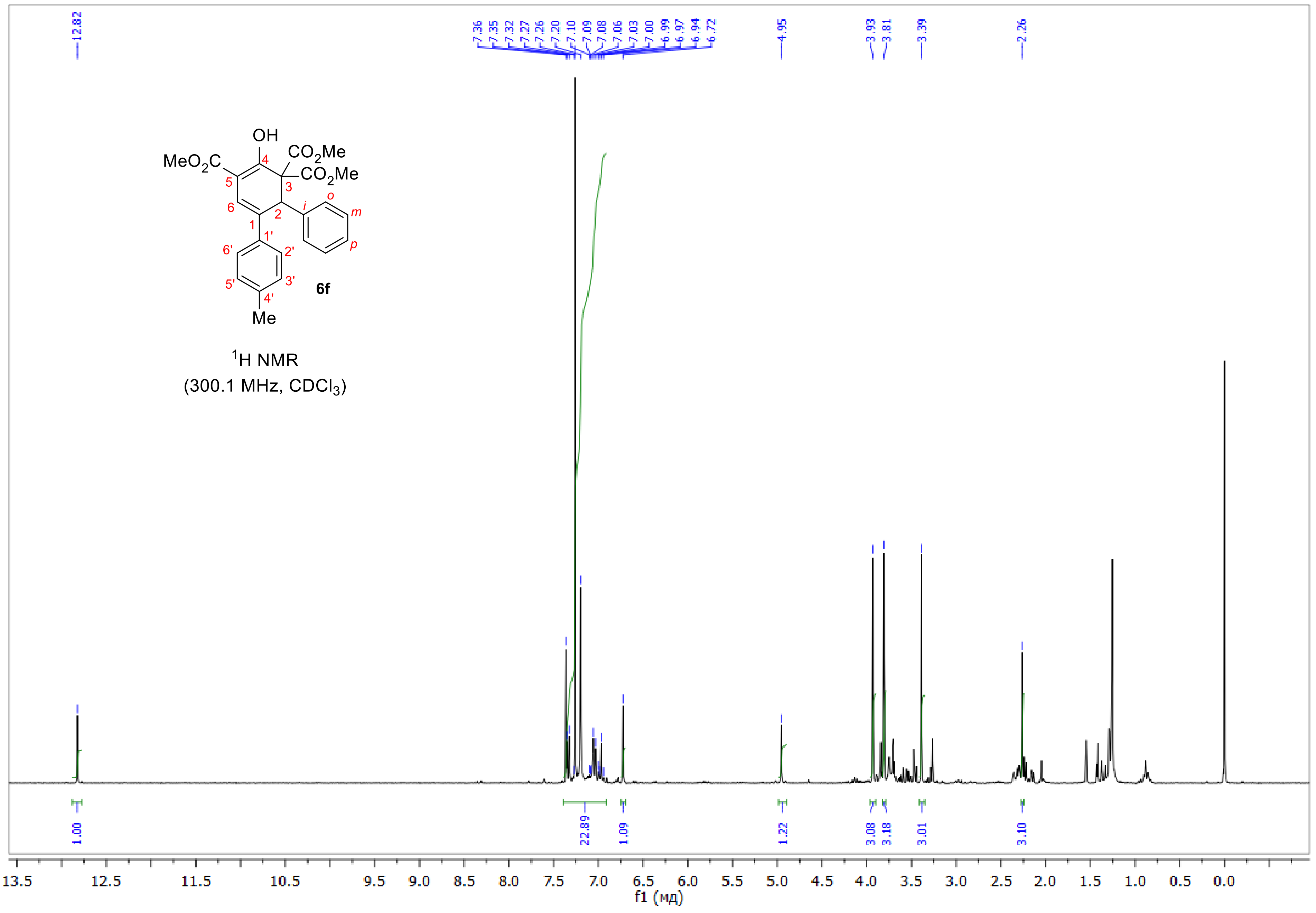

12.82
7.36 7.35 7.32 7.27 7.26 7.20 7.10 7.09 7.08 7.06 7.03 7.00 6.99 6.97 6.94 6.72
4.95
3.93 3.81
3.39
2.26
OH
MeO_2C
CO_2Me
CO_2Me
6f
Me
1H NMR
(300.1 MHz, $CDCl_3$)
1.00
22.89
1.09
1.22
3.08
3.18
3.01
3.10
f1 (мд)

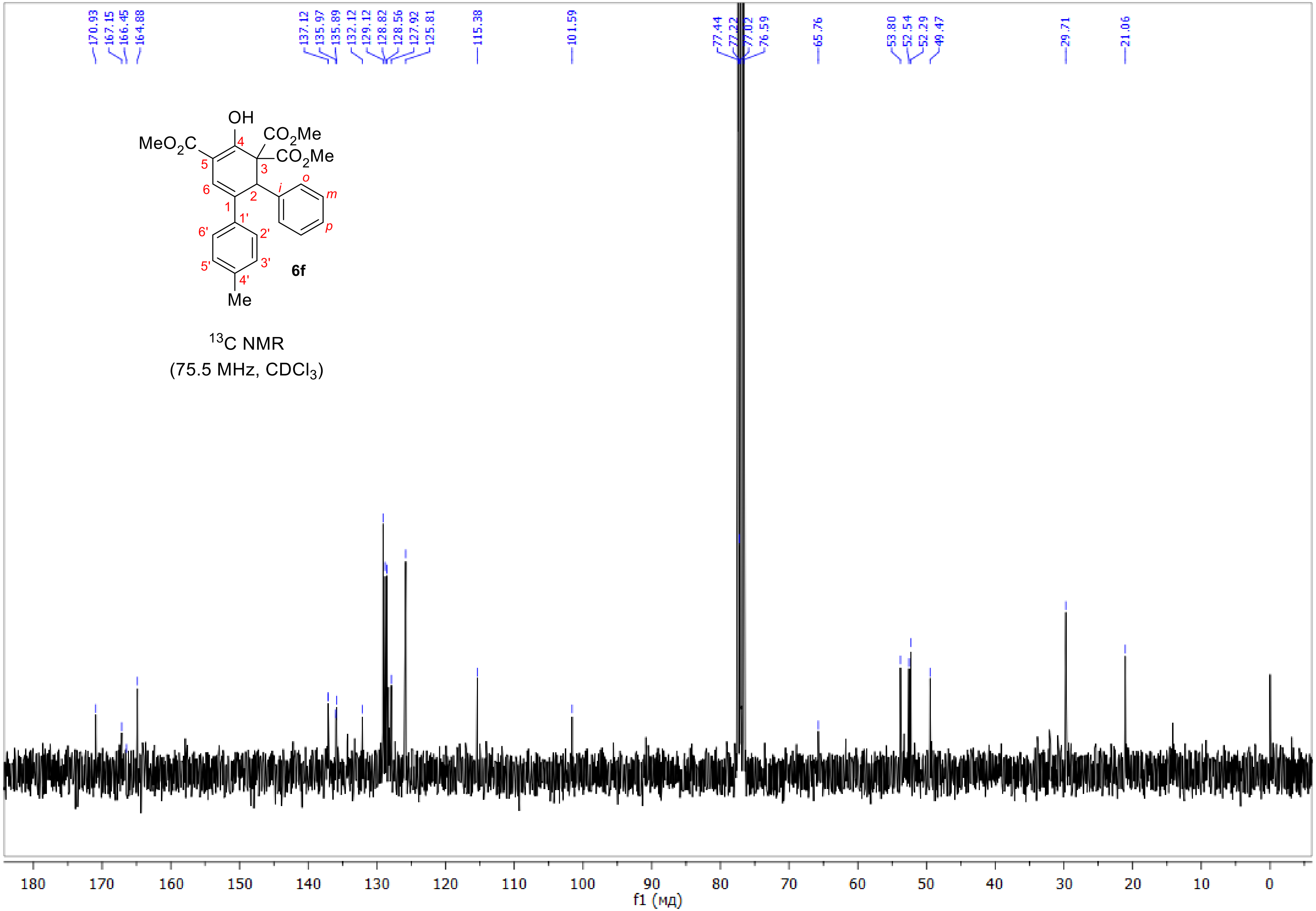
170.93
167.15
166.45
164.88
137.12
135.97
135.89
132.12
129.12
128.82
128.56
127.92
125.81
115.38
101.59
77.44
77.22
77.02
76.59
65.76
53.80
52.54
52.29
49.47
29.71
21.06
OH
MeO2C
CO2Me
CO2Me
Me
6f
13C NMR
(75.5 MHz, CDCl3)
f1 (мд)

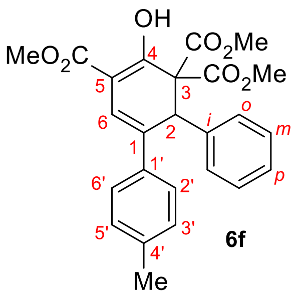

6f

$^{1}H,^{13}C$-HSQC
(^{1}H: 300.1 MHz,
^{13}C: 75.5 MHz
$CDCl_3$)

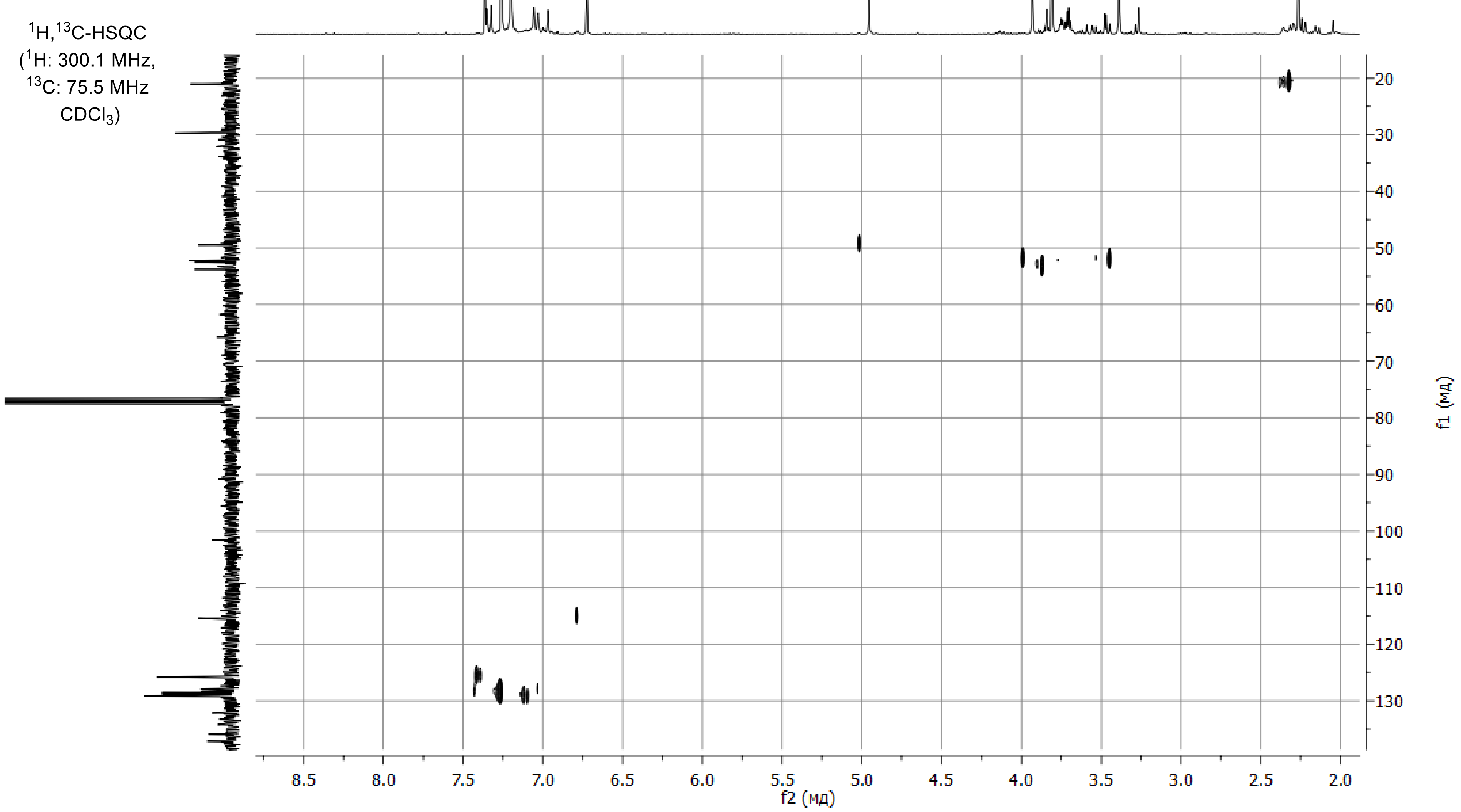

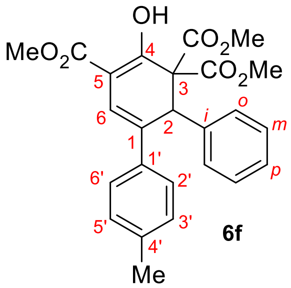

6f

$^{1}H,^{13}C$-HMBC
(^{1}H: 300.1 MHz,
^{13}C: 75.5 MHz
$CDCl_3$)

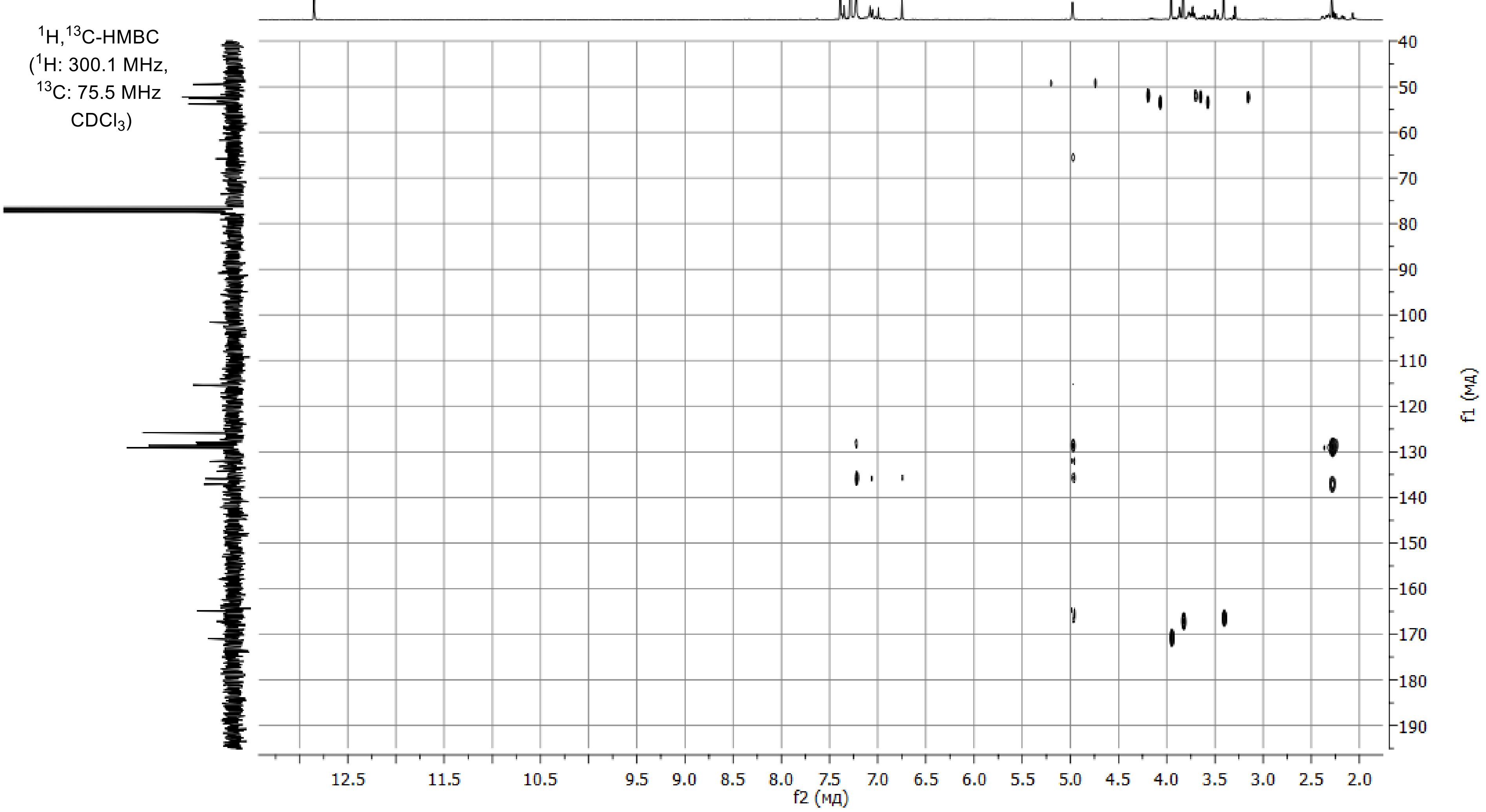

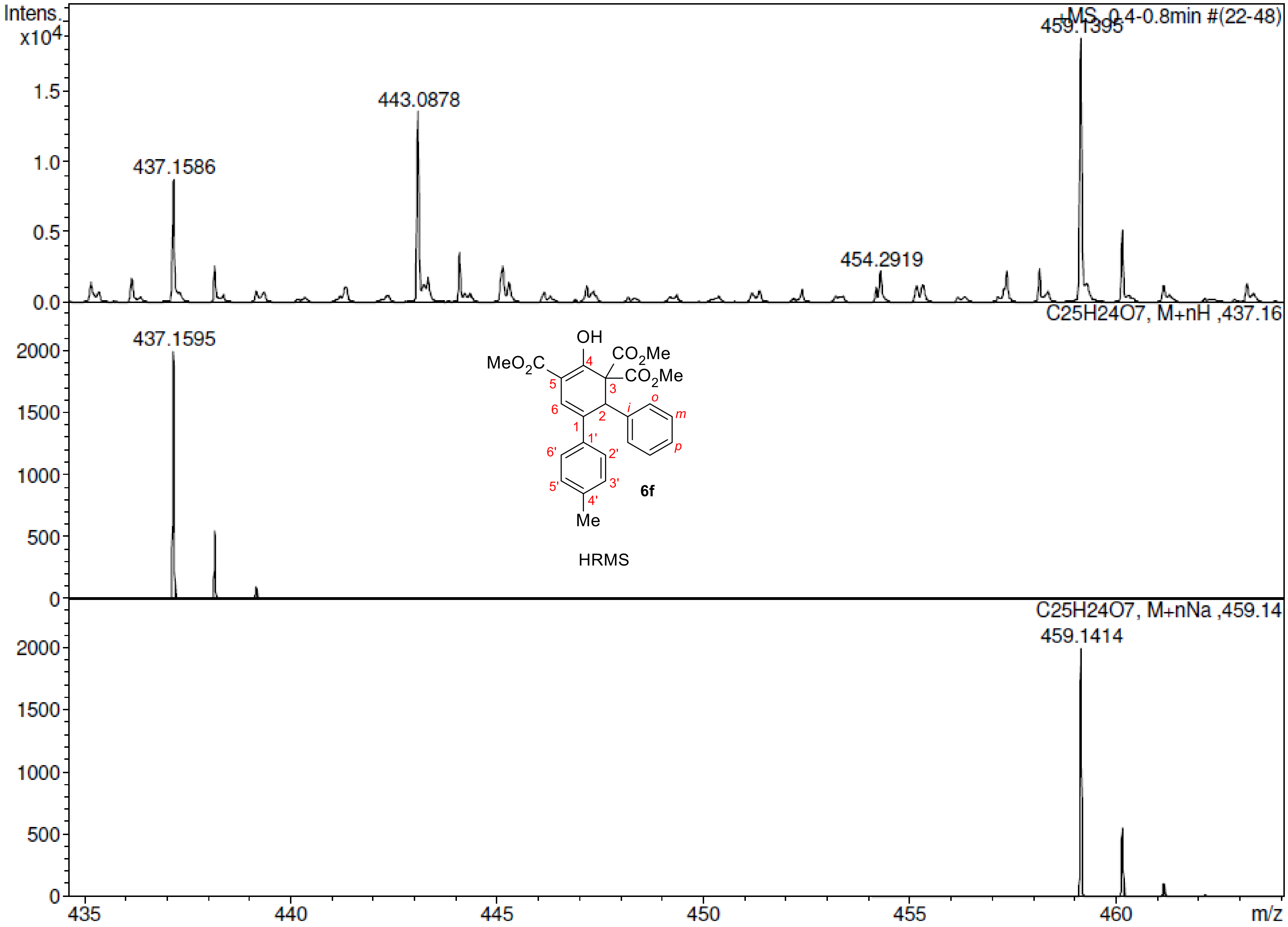
Intens.
x10^4
+MS, 0.4-0.8min #(22-48)
459.1395
443.0878
437.1586
454.2919
C25H24O7, M+nH ,437.16
437.1595
C25H24O7, M+nNa ,459.14
459.1414
6f
HRMS
m/z
